# Sorafenib as first-line therapy for metastatic uveal melanoma: A multicenter, placebo-controlled randomized discontinuation study (STREAM)

**DOI:** 10.1016/j.isci.2025.114045

**Published:** 2025-11-14

**Authors:** Halime Kalkavan, Max E. Scheulen, Eckhart Kämpgen, Ulrich Keilholz, Lucie Heinzerling, Smiths S. Lueong, Annalena Hlinka, Tanja Gromke, Sebastian Ochsenreither, Ralf-Axel Hilger, Matthias Grubert, Axel Wetter, Nika Guberina, Michael Zeschnigk, Peter Ferency, Swantje Held, Axel Hinke, Gerold Schuler, Karim Al-Ghazzawi, Norbert Bornfeld, Nikolaos E. Bechrakis, Martin Schuler, Sebastian Bauer, Heike Richly, Jens T. Siveke

**Affiliations:** 1 Medical Faculty, University Duisburg-Essen, 45147 Essen, Germany; 2 Department of Medical Oncology, West German Cancer Center, University Medicine Essen, 45147 Essen, Germany; 3 Department of Dermatology, University Hospital Erlangen, 91012 Erlangen, Germany; 4 Dermatologikum Berlin, 10117 Berlin, Germany; 5 Charité, Comprehensive Cancer Center, 10117 Berlin, Germany; 6 Department of Dermatology and Allergy, University Hospital Munich (LMU), 80802 Munich, Germany; 7 Division of Solid Tumor Translational Oncology, German Cancer Consortium (DKTK, Partner Site Essen) and German Cancer Research Center, DKFZ, 45147 Heidelberg, Germany; 8 Department of Radiology, West German Cancer Center, University Medicine Essen, 45147 Essen, Germany; 9 Department of Radiotherapy, University Hospital Essen, University of Duisburg-Essen, 45147 Essen, Germany; 10 Department of Human Genetics, University Medicine Essen, 45147 Essen, Germany; 11 ClinAssess, 51379 Leverkusen, Germany; 12 CCRC - Cancer Clinical Research Consulting, 45147 Düsseldorf, Germany; 13 Department of Ophthalmology, West German Cancer Center, University Medicine Essen, 45147 Essen, Germany; 14 Bridge Institute of Experimental Tumor Therapy, West German Cancer Center, University Medicine Essen, 45147 Essen, Germany; 15 National Centre for Tumour Diseases (NCT) West, Campus Essen, 45147 Essen, Germany

**Keywords:** Health sciences, Medicine, medical specialty, oncology

## Abstract

Almost every second patient with uveal melanoma develops metastatic disease, with a usually fatal outcome within one year. The scarcity of broadly applicable systemic therapies further limits patient survival. In this multicenter, placebo-controlled randomized discontinuation phase 2 trial, we evaluated the efficacy of the multi-kinase inhibitor sorafenib in treatment-naïve patients with metastatic uveal melanoma (mUM). After a 56-day *run-in* period with 400 mg bid sorafenib in a total of 147 patients, randomization was performed in 78 patients with stable disease assigned to blinded sorafenib or placebo in a 1:1 ratio. The primary endpoint of the study was met with a significantly higher median progression-free survival (PFS) in the sorafenib group compared to placebo (5.5 vs. 1.9 months, HR = 0.53, *p* = 0.0083). First-line treatment with sorafenib was well tolerated and showed promising efficacy in patients with mUM. The detection of *GNAQ*/*GNA11* mutations in ctDNA at baseline was associated with an inferior outcome. (ClinicalTrials.gov: NCT01377025).

## Highlights

• Sorafenib was well-tolerated and showed clinical efficacy in metastatic uveal melanoma
• Sorafenib improved progression-free survival (5.5 vs. 1.9 months, p = 0.0083)
• CtDNA and GGT >50 U/L identified as potential negative predictive biomarkers
• Randomized discontinuation trial design suited mUM’s aggressive characteristics

## Graphical abstract

**Figure:**
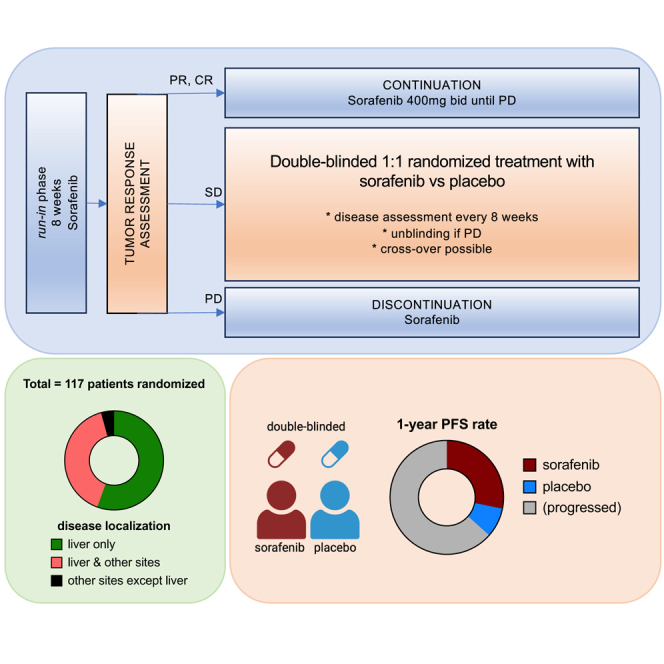

## Introduction

Uveal melanoma is a rare cancer entity with an age-adjusted incidence of approximately 5 per million people.^1^ About half of these patients develop metastatic disease, which is primarily observed in the liver (over 90% of cases), rarely metastases occur in the lung (16% of patients) or less than 10% of patients in bone, brain, and soft tissues.^2^ Despite minor advancements in metastatic uveal melanoma (mUM) treatment, survival rates of patients have remained nearly unchanged in the past 40 years, with a median overall survival of 6–12 months regardless of the therapeutic approach.^3^ To date, the gp100-targeting bispecific fusion protein tebentafusp is the only FDA and EMA approved systemic treatment for mUM.^4^^,^^5^ Due to its HLA-A∗02:01-restrictive application and the fact that approximately 45% of American and European people are HLA-A∗02:01-positive, this treatment is only applicable in less than every second patient with mUM. Within the limitations of a phase 3 clinical trial, tebentafusp has shown an increase in estimated median overall survival (OS) duration of patients with uveal melanoma, from 16 months (control group, investigator’s choice) to 21.7 months.^6^

However, various other studies in patients with metastatic UM have demonstrated disappointing response rates with various chemotherapeutic, targeted, and immune-based approaches.^7^ Molecularly, mutually exclusive gain-of-function mutations in *GNAQ* and *GNA11* genes, which encode for Gα subunits of G-proteins, are found in 85–95% of uveal melanomas and drive tumor initiation.^8^^,^^9^ These mutations lead to loss of guanosine triphosphate hydrolase (GTPase) activity and constitutive activation of the mutated Gα proteins with the downstream activation of multiple signaling pathways, including the phosphoinositide-3-kinase, the mitogen-activated protein kinase (MAPK), and the protein kinase C pathway.^10^ Yet, efforts to target MAPK signaling either alone or in combination with chemotherapy have remained disappointing.^11^^,^^12^ Promising new agents such as the antibody-drug conjugate DYP688 (NCT05415072) targeting GNAQ/GNA11 and PMEL17 or protein kinase C (PKC) inhibitors (darovasertib, NCT05987332) are still in (early) clinical trials and thus are mostly inaccessible for patients.

**Figure 1:**
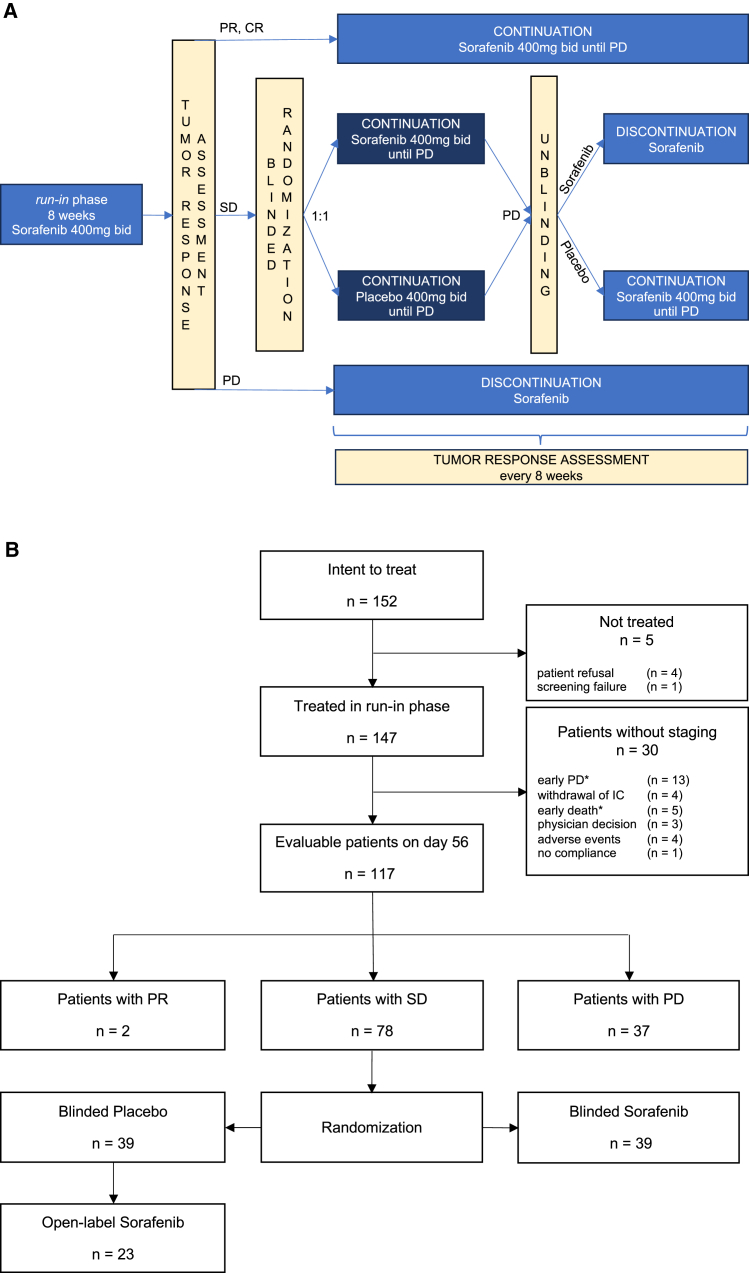
Clinical trial design and patient recruitment (A) Scheme visualizes the design of the randomized discontinuation trial (RDT). (B) Flow diagram of patient recruitment in screening, enrollment, randomization, and intervention phases. PR, partial remission; CR, complete remission; SD, stable disease; PD, progressive disease; bid, *bis in die* (twice daily), ∗ included in the prognostic analysis (*n* = 135).

Sorafenib is a multi-kinase inhibitor that targets MAPK signaling at the level of Raf-kinase (Raf1, B-Raf), VEGF-1-3, PDGFR-b, c-Kit, FLT-3, and RET, with the inhibition of tumor cell proliferation and angiogenesis.^12^ It has been approved by the FDA and EMA for the treatment of renal and hepatocellular cancer.^13^^,^^14^ Importantly, sorafenib has shown activity in a human uveal melanoma xenograft model.^15^ We previously observed good drug tolerability and clinical activity in a Good Clinical Practice (GCP)-adapted register trial,^16^ which served as a rationale for the STREAM investigator-initiated trial investigating sorafenib as first-line treatment in chemonaïve patients with mUM (Figure 1A).

## Results

### Patient and treatment characteristics

Out of 152 consecutive eligible patients (intent-to-treat population), 147 patients (96.7%) were treated with sorafenib (Bayer AG, Leverkusen, Germany) in an open-label run-in period at a dose of 400 mg bid. Of these, 117 patients (79.6%) were evaluated at day 56 (Figure 1). Tumor response assessment and classification according to RECIST revealed two patients with partial remission (PR; 1.7%), who were subsequently continued on sorafenib. A total of 37 patients had progressive disease (PD; 31.6%) and were taken off the study. The remaining 78 patients had stable disease (SD; 66.7%) and were randomly assigned in a 1:1 ratio to either sorafenib or placebo in a double-blinded fashion without stratification. Patients were evaluated every 8 weeks for efficacy and safety by alternate visits and telephone interviews, or in case of clinical signs of PD or sequelae. Patients with PD after randomization were unblinded. A total of 23 patients who had been on placebo were crossed over to sorafenib.

Patient and disease characteristics are shown in Table 1 and Figure S1. Overall, a sex imbalance among 29 female (37.2%) and 49 male (62.8%) patients was conceivable. The median lead time between primary intraocular tumor and detection of metastasis was 26.9 months (2.2 years) for all evaluable patients. There were no statistically significant differences in the lead time between patients excluded during the run-in phase due to PD and the randomized groups (Figure S2). Almost all patients had liver metastases, either with (40%) or without (56%) metastasis to other organs. Most frequently (co-)metastasized organs were the lungs (19%), bones (14%), and lymph nodes (10%). Only one patient had isolated metastasis to the lungs.

**Table 1:** Characteristics of Evaluable Patients on day 56 (run-in Phase) and randomized patients

	Overall evaluable patients on day 56 (n = 117)		Randomized Set					
			Overall (*n* = 78)		Placebo (*n* = 39)		Sorafenib (*n* = 39)	
Gender								
Male	69	59%	49	63%	26	67%	23	59%
Female	48	41%	29	37%	13	33%	16	41%
Age (years, median, range)	62	23–88	63.5	23–88	66	47–88	58	23–79
ECOG (evaluable patients)	106		71		35		36	
ECOG 0 (n)	85	80%	56	79%	27	77%	29	81%
ECOG 1 (n)	20	19%	14	20%	7	20%	7	19%
ECOG 2 (n)	1	1%	1	1%	1	3%	0	0%
Time since first diagnosis (months, median, range)	34.0	1.3–431	35.0	1.7–431	37.9	2.2–431	33.1	1.7–405
Localization of metastases								
Liver only	65	56%	38	49%	18	46%	20	51%
Liver and other site(s)	47	40%	37	47%	19	49%	18	46%
Other site(s) except liver	5	4%	3	4%	2	5%	1	3%
Number of organ sites								
1 site	66	56%	39	50%	19	49%	20	51%
2 sites	31	27%	24	31%	14	36%	10	26%
≥ 3 sites	20	17%	15	19%	6	15%	9	23%
GGT (U/L, mean ± SD)	103 ± 125		80 ± 81		100 ± 88		60 ± 70	60 ± 70
LDH (U/L, mean ± SD)	364 ± 289		321 ± 231		364 ± 279		279 ± 162	279 ± 162
S100 (μg/L, mean ± SD)	0.2 ± 0.5		0.2 ± 0.6		0.3 ± 0.8		0.1 ± 0.1	0.1 ± 0.1

Data are shown as number (%) unless otherwise specified. SD = standard deviation.

### Safety and tolerability

**Figure 2:**
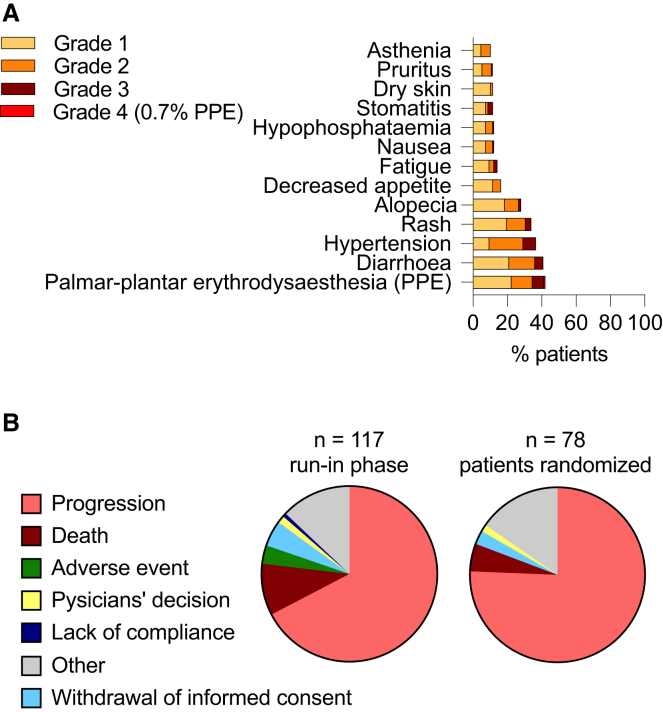
Very common adverse events and reasons for therapy cessation (A) Percentage of patients experiences indicated common adverse events. Only 1 patient had a grade 4 palmar-plantar erythrodysaesthesia syndrome (PPE). Total *n* = 117. (B) Parts of whole graphs show the percentage of patients with indicated reasons for ending therapy.

The most common treatment-related adverse events reported in the safety set of 147 patients were skin disorders, such as palmar-plantar erythrodysaesthesia (PPE; 44%), rash (34%), alopecia (29%), dry skin (12%), and pruritus (12%), or gastrointestinal symptoms, such as diarrhea (41%), decreased appetite (16%), nausea (12%), and stomatitis (12%), or concerning the general condition of patients as fatigue (14%) and asthenia (10%), or were of vascular origin such as arterial hypertension (37%) or concerned laboratory changes, such as hypophosphatemia (12%) (Figure 2A). Most of such adverse events were limited to grade 1 or 2. However, 42 patients experienced grade 3 toxicities with PPE (*n* = 10), hypertension (*n* = 11), diarrhea (*n* = 7), rash (*n* = 5), stomatitis (*n* = 4), nausea (*n* = 3), and decreased appetite (*n* = 2). There was only 1 case of a grade 4 adverse event with PPE and no grade 5 toxicities. Of all 147 patients, 69 (46.9%) needed a dose reduction at least once. Five patients had to end therapy due to adverse events in the run-in phase, but none of the randomized patients had to stop treatment because of drug toxicity (Figure 2B). No patient died from toxicity.

### Efficacy

**Figure 3:**
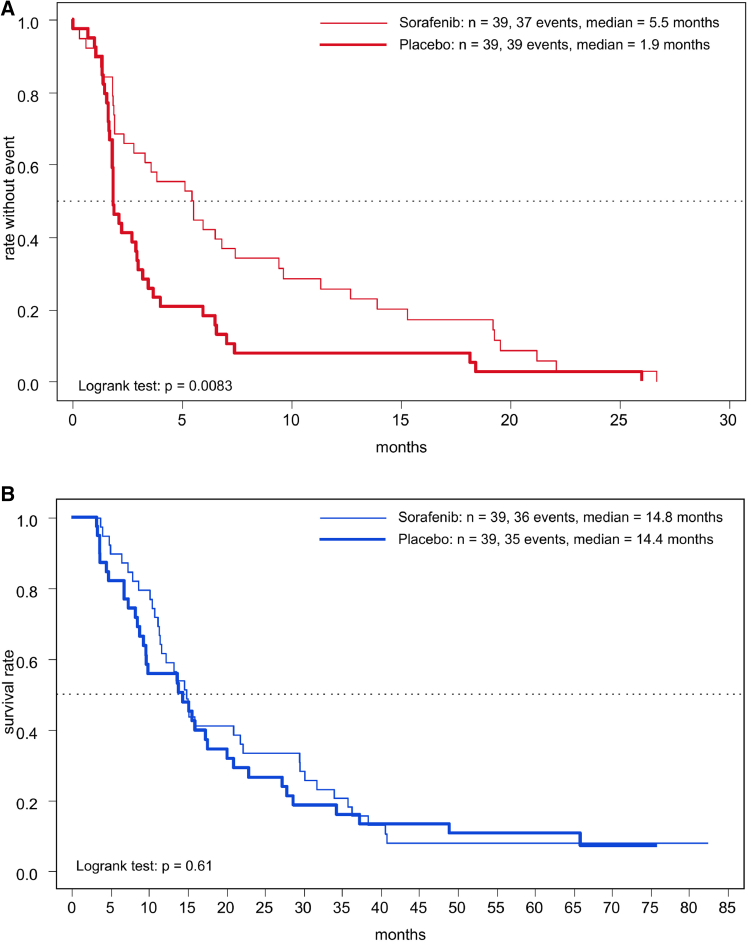
Kaplan-Meier estimates of survival Kaplan-Meier plot (A) of investigator-assessed progression-free survival (PFS) from day 56 randomization for patients randomized to placebo (*n* = 39) or to sorafenib (*n* = 39) (blinded phase) and (B) of overall survival (OS) run-in period included.

Investigator-assessed median progression-free survival (PFS) from day 56 of randomization was significantly longer (5.5 months) for the 39 patients randomized to sorafenib than for the 39 patients randomized to placebo (1.9 months; HR = 0.53, *p* = 0.0083) (Figure 3A). The 12-month PFS rate was 28.2% for sorafenib vs. 8.4% for placebo. Post randomization, the best responses in the placebo group included 24 stable diseases, one patient with spontaneous partial remission, and 14 with disease progression. In the sorafenib randomized group best responses were 28 stable diseases, 3 partial remissions, and 8 progressive diseases. Median OS, including the run-in period, was not different with 14.8 months for 39 patients randomized to sorafenib vs. with 14.4 months for 39 patients randomized to placebo (HR = 0.85, *p* = 0.51) (Figure 3B). Notably, the cross-over option, which was provided for the placebo group, led to a switch from placebo to sorafenib in 23 patients (59.0%) after unblinding because of progressive disease (Figure 1) with a median PFS of 10 weeks. These patients continued treatment with sorafenib for a mean of approximately 3.5 months (Figure S3). Two patients died within 1 day after unblinding. The longest time to progression in the open-label phase (after crossover from placebo to sorafenib) was 17 months.

### Prognostic factors and subgroup analyses

Neither age nor gender of the patients showed any relevant prognostic impact on long-term endpoints. Table 2 presents an overview of the disease-related baseline characteristics, which proved to be prognostic for either PFS or OS at a level of *p* ≤ 0.1. With respect to PFS, clinical parameters such as a short relapse-free interval, metastatic dissemination beyond the liver, elevated LDH, and elevated GGT showed negative prognostic value (Table 2; Figure S4).

**Table 2:** Univariable and multivariable analyses of baseline characteristics on PFS and OS

Prognostic factor^a^	Statistical parameter	PFS			OS		
		univariable (*n* = 135–124)	Multi-variable full model (*n* = 102)	Multi-variable reduced model^b^ (*n* = 103)	univariable (*n* = 135–124)	Multi-variable full model (*n* = 97)	Multi-variable reduced model^b^ (*n* = 98)
Relapse-free interval≤ 2 years	Hazard ratio	1.43	1.68	1.69	1.25	–	–
	95% CI	1.01–2.03	1.11–2.56	1.11–2.56	0.87–1.79	–	–
	P	0.042	0.015	0.014	0.22	–	–
Metastasis extrahepatic	Hazard ratio	1.63	1.52	1.50	1.20	–	–
	95% CI	1.15–2.31	1.00–2.33	0.99–2.27	0.84–1.71	–	–
	P	0.0058	0.048	0.055	0.32	–	–
LDH >240 U/L	Hazard ratio	1.55	1.22	–	2.01	1.22	–
	95% CI	1.09–2.21	0.74–2.00	–	1.39–2.89	0.72–2.05	–
	P	0.014	0.43	–	0.00015	0.46	–
GGT >50 U/L	Hazard ratio	1.43	1.47	1.59	3.51	2.84	3.04
	95% CI	1.19–1.72	0.87–2.48	1.00–2.54	2.35–5.23	1.68–4.79	1.88–4.90
	P	<0.0001	0.16	0.049	<0.0001	0.00010	<0.0001
ctDNA	Hazard ratio	2.38	2.22	2.22	2.33	1.54	1.52
	95% CI	1.56–3.7	1.35–3.7	1.35–3.57	1.52–3.45	0.99–2.38	0.98–2.38
	P	(<0.0001)	0.0015	0.0017	<0.0001	0.054	0.060
S100 >0.105 μg/L	Hazard ratio	1.30	–	–	1.92	1.56	1.66
	95% CO	0.90–1.87	–	–	1.31–2.80	0.99–2.44	1.09–2.54
	P	(0.17)	–	–	0.00066	0.053	0.019

Only variables with a univariable *p* ≤ 0.1 in either endpoint are included.

CI, confidence interval; –, not included in model.

a The provided category denotes the group for which the relative risk is calculated relative to the complementary reference group. HR > 1.0 corresponds to a higher risk.

b After stepwise elimination of parameters with *p* > 0.1.

Median OS of the total study population was 11.2 months. The clinical disease parameters, such as gender, age, relapse-free interval, and metastasis beyond the liver did not show a significant prognostic impact. However, shorter OS was associated with laboratory markers in patients with GGT >50 U/L (medians: 7.2 vs. 17.2 months; HR = 3.51, *p* < 0.0001) and S100 > 0.105 μg/L (6.5 vs. 13.7 months; HR = 1.92, *p* = 0.00066) (Table 2; Figure S5). These parameters showed independent relevance in multivariable analysis.

Treatment group comparisons for PFS were performed in all prognostic subgroups defined by the relevant factors described above (data not shown). Although the Kaplan-Meier curves uniformly suggest a somewhat larger relative effect size in the “high-risk” subgroups, there is no clear indication of a distinctly predictive factor for the efficiency of the kinase inhibitor. Formal statistical confirmation of any interaction is precluded due to the small sample sizes in the subgroups.

### Predictive value of baseline plasma-based circulating tumor DNA analysis

**Figure 4:**
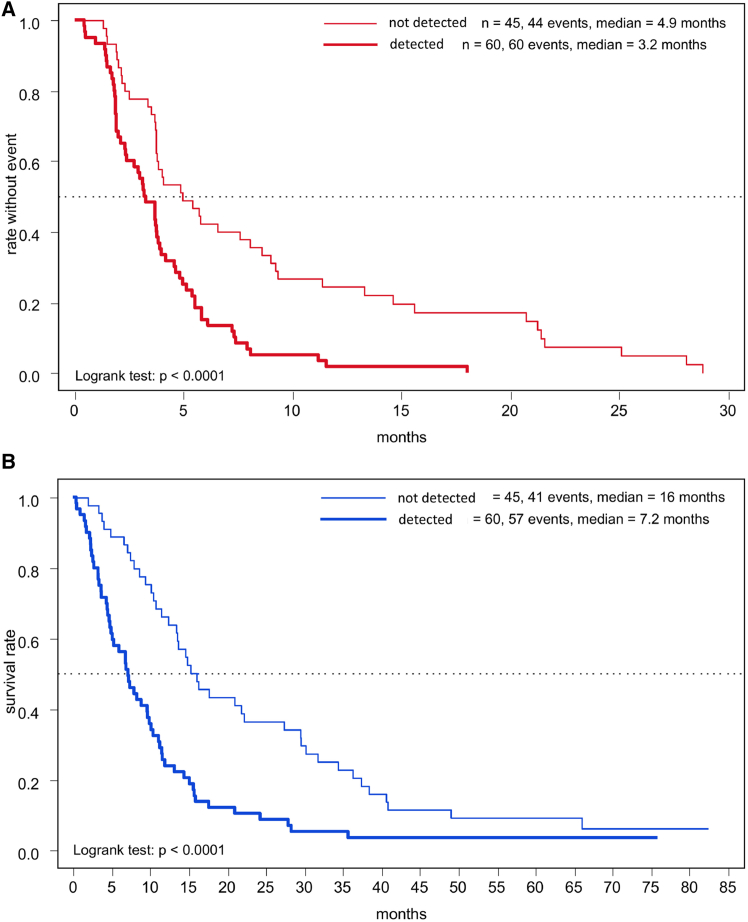
Patient survival based on ctDNA detection Kaplan-Meier plots of (A) progression-free survival (PFS) and (B) overall survival (OS) dependent on ctDNA-detection based on GNAQ/GNA11 mutation.

Mutations at the *GNAQ* locus were found in 35/121 (28.9%) plasma samples at baseline, and an equal number (35/121) with a mutation at the GNA11 locus. All GNAQ and GNA11 mutations at baseline were mutually exclusive. In total, ^*mut*^*GNAQ*/*GNA11* could be detected in 70/121 patients (57.9%). *GNAQ* mutation load varied from about 8 to 8.000 (median 88) copies/mL. For GNA11, the mutation load varied from about 9 to more than 20.000 (median 193) copies/mL of plasma. Patients with circulating tumor DNA (ctDNA), defined by ^*mut*^*GNAQ* or ^*mut*^*GNA11* detection in cell free DNA (cfDNA), lived markedly shorter (medians: 7.2 vs. 16.0 months; HR = 2.33, *p* < 0.0001) (Table 2; Figure 4). Moreover, ctDNA detection was significantly associated with unfavorable PFS at both univariable and multivariable levels, respectively (HR = 2.38, 95% CI = 1.56–3.7, *p* < 0.0001 and HR = 2.22, 95% CI = 1.35–3.57, *p* = 0.0017) (Table 2; Figure 4).

## Discussion

Patients with mUM are particularly insensitive to systemic chemotherapy and immune checkpoint inhibitors.^5^ Although gain-of-function mutations in *GNAQ* and *GNA11* genes have been identified as driver mutations in approximately 95% of UM,^8^^,^^9^ there are no clinically approved therapies that target the associated pathways in mUM. The very promising antibody-drug conjugate DYP688 targeting GNAQ/GNA11 and PMEL17 is still in the phase I/II clinical trial (NCT05415072). Yet another mechanistically relevant drug, the protein kinase C (PKC) inhibitor darovasertib, is also still in a phase 2b clinical trial for HLA-A2 negative patients as a combinational therapy with the multi-kinase inhibitor crizotinib (NCT05987332). Consequently, there is a substantial need for accessible, efficient therapeutics for HLA-A∗02:01-negative patients with mUM and HLA-A∗02:01-positive patients who are resistant to tebentafusp or discontinuing treatment because of severe side effects.^6^^,^^17^

Sorafenib is a multi-kinase inhibitor that predominantly targets MAPK signaling and thus acts downstream of the driver mutations in *GNAQ/GNA11*.^12^ Since sorafenib is clinically approved for the treatment of renal and hepatocellular cancer, its safety profile is well-defined, and side effects such as palmar-plantar erythrodysaesthesia, diarrhea, hypertension, and nausea are well-known and manageable.^13^^,^^14^ Accordingly, our study medication was tolerable, and adverse events were consistent with the established safety profile of sorafenib. Thus, overall sorafenib-based kinase inhibition was feasible in this population.

In line with previous preclinical^15^ and preliminary clinical studies,^16^^,^^18^ sorafenib showed promising efficacy in patients with mUM in this investigator-initiated trial investigating sorafenib as first-line treatment in chemonaïve patients with mUM. Sorafenib significantly reduced the risk of progression (HR = 0.53) compared to placebo with an increase in median progression-free survival (PFS) to 5.5 months during the randomization phase of the trial (control group 1.9 months, *p* = 0.0083). This benefit was further reflected in a significantly increased 12-month PFS rate of 28.2% for sorafenib vs. 8.4% for placebo. Taking differences in trial design and patient populations into account, similar results had been observed in clinical trials with ICB or tebentafusp. For instance, a single arm clinical trial with nivolumab and ipilimumab showed median PFS was 5.5 months, but with very frequent grade 3 and 4 toxicities, namely 20%.^19^ Treatment with tebentafusp revealed an increase in median PFS of 3.4 months compared to 2.9 months of the control group.^17^

Considering that mUM is a rare disease with yet ill-defined prognostic factors, the randomized discontinuation trial (RDT) design was reasonable for the objective evaluation of therapy response. It considers that a bimodal survival pattern is strongly evident in the natural course of the disease, with patient survival lasting from a few months to many years following the primary diagnosis of mUM.^20^^,^^21^^,^^22^^,^^23^^,^^24^^,^^25^ Importantly, the RDT design is a feasible phase II study design for evaluating possible activity of cytostatic anticancer agents as opposed to cytotoxic drug effects,^26^ such as the multi-kinase inhibitor sorafenib. Conclusively, its primary clinical benefit is expected to be disease stabilization rather than tumor shrinkage. Here, the RDT study design appeared especially useful when distinguishing treatment responders from slow-growing disease during SD.^26^^,^^27^^,^^28^ Yet, for the same reason, this trial design has been criticized for potentially introducing selection bias toward more indolent disease.

In this RDT, all patients received the study drug for an initial run-in period, followed by the random assignment of potential responders either to the study drug or placebo, to be able to distinguish the anticancer activity of the drug and the natural course of the underlying disease.^26^^,^^27^^,^^28^^,^^29^ This RDT design allowed the continuation of sorafenib treatment beyond the run-in phase in two patients with partial remission. Vice versa, a subset of highly aggressive, unresponsive mUM cases of this rare disease, which accounted for 31.6% of recruited patients in this clinical trial, had disease progression during the run-in phase and were taken off study. This subpopulation was smaller than expected, since, as per study protocol, an estimate of 50% was given by educated guess.^20^^,^^21^^,^^22^^,^^23^^,^^24^^,^^25^ Consequently, the remaining 78 patients who had stable disease (66.7%) were randomly assigned to blinded sorafenib or placebo in a 1:1 ratio. While this design further solidifies that the observed increase in PFS in sorafenib-receiving patients was most likely a direct consequence of treatment, it limits the evaluation of OS. The cross-over option, which was provided for the placebo group, led to a switch from placebo to sorafenib in 23 patients (59.0%) after unblinding because of progressive disease (PD), which further limits conclusions on OS. Using a rank-preserving structural failure time model (RPSFTM) to reveal an occult *true* overall survival (OS) rate, which is commonly used in clinical studies with a crossover design, was not appropriate due to limited patient numbers and a high crossover rate.^30^

In line with earlier studies in mUM, our work further emphasizes the need for prognostic markers and patient stratification to identify subpopulations that would mostly benefit from potentially effective therapeutics. Earlier studies identified lactate dehydrogenase (LDH), thrombocyte counts, aspartate transaminase, and the metastasis-free interval at first diagnosis of metastatic disease as predictors for overall survival, independent of treatments conducted during the course of disease.^20^^,^^25^^,^^31^ In a previous study, we reported that ctDNA could be detected in patients with uveal melanoma via ultradeep sequencing of plasma-based *GNAQ* or *GNA11* mutation.^32^ In STREAM, we prospectively evaluated the presence of ctDNA by analyzing for ^*mut*^*GNAQ* or ^*mut*^*GNA11* in the plasma of 121 patients at the baseline time point using a standardized digital droplet PCR approach. As expected, *GNAQ* and *GNA11* mutations were mutually exclusive. Notably, ctDNA detection was associated with an unfavorable response to sorafenib therapy and shortened PFS as well as OS. In the multivariate analysis, the presence of ctDNA-based *GNAQ/GNA11* mutation was the most significant factor associated with PFS, showing a higher hazard ratio than, for example, LDH, a well-established clinical prognostic parameter. This finding was further confirmed in the following clinical trials of tebentafusp and darovasertib, where ctDNA clearance during treatment was associated with increased OS and thus indicates its potential as a predictive marker for therapy response and resistance, respectively.^17^^,^^33^ Conclusively, the utilization of liquid biopsy by the detection of ctDNA can provide a useful tool both for early metastasis detection, as well as an additional predictive marker in patients with mUM.^34^^,^^35^^,^^36^ Yet, further research with longitudinal sample collection is necessary to solidify such a role for sorafenib using mutant GNAQ/GNA11 ctDNA. Moreover, we identified increased GGT >50 U/L (medians: 7.2 vs. 17.2 months; HR = 3.51, *p* < 0.0001) and S100 > 0.105 μg/L (6.5 vs. 13.7 months; HR = 1.92, *p* = 0.00066) as negative prognostic markers for OS.

Taken together, the phase II STREAM study showed that sorafenib provided a significant PFS benefit for chemonaïve patients with metastatic uveal melanoma. A subsequent realization of a phase III trial of sorafenib versus placebo is, in our view, not advisable in this orphan disease. Instead, future studies may include multi-kinase inhibition in combination with other promising and rational therapeutic strategies to improve patient outcomes in this fatal disease. Notably, the combination of anti-angiogenic targeting with immune checkpoint blockade is a promising concept in various cancer entities.^37^ In metastatic uveal melanoma with its current limited treatment options, sorafenib appears to be an effective therapeutic alternative with low and manageable side effects and easy administration due to oral intake, thereby providing an option to prolong PFS.

### Limitations of the study

The RDT design with the randomization of patients with SD upon sorafenib treatment was key to drawing any conclusions on the cytostatic, thus the disease stabilizing effects of sorafenib. However, one might argue that the RDT design selects a more indolent, homogeneous group than the real-world patient population. Nevertheless, using a run-in phase and the option that patients who progress during the randomized phase might be crossed over to the investigational drug after unblinding enabled an ethically justifiable use of placebo as a comparator arm within an oncology trial. For instance, it allowed the (re-)crossover of a patient with PD after 199 days of placebo to sorafenib, which resulted in an additional 478 days of SD in our STREAM-trial. Yet, the cross-over design generally complicates or even disables the interpretation of OS since it may reintroduce imbalance.

## Resource availability

### Lead contact

Further information and reasonable requests for resources should be directed to and will be fulfilled by the lead contact, Halime Kalkavan (halime.kalkavan@uk-essen.de).

### Materials availability

This study did not generate new unique reagents.

### Data and code availability

• Reasonable requests for data resources should be directed to and will be fulfilled by the lead contact.
• This study did not generate any original code.
• Any additional information required to reanalyze the data reported in this article is available from the lead contact upon request.

## Acknowledgments

We thank all patients and their families for participation in the trial and the study teams at the centers for their dedicated work. We thank 10.13039/100004326Bayer Pharmaceuticals, Leverkusen, Germany, for their support. We thank Nicole Bielefeld for technical assistance. This Investigator Initiated Trial (IIT) was supported by a grant from 10.13039/100015739Bayer Vital GmbH, Leverkusen, Germany. Medical monitoring and evaluation were performed by ClinAssess GmbH, Leverkusen, Germany. H.K. was supported by grants from the Advanced Clinician Scientist Program UMEA^2^ (Medical Faculty, University Duisburg-Essen), the Federal Ministry of Research, Technology and Space 01EO2104 (BMFTR), and the 10.13039/501100005972German Cancer Aid (Max-Eder Junior Research Group Program, DKH 70115382). We acknowledge the support of the Open Access Publication Fund of the 10.13039/501100008349University of Duisburg-Essen. The preliminary clinical results of this study have been presented at the Annual Meeting of the American Society for Clinical Oncology, Chicago, 2017 (J Clin Oncol 2017;35:9511. abstract). The graphical abstract was generated with BioRender.

In memoriam of Reinhard Becher, MD/PhD, who was one of the first to describe chromosomal abnormalities and the prognostic implications of monosomy 3 in uveal melanoma.^38^

## Author contributions

G.S., M.E.S., and E.K. conceived and designed the study. H.K., A.H., S.B., M.G., N.G., L.H., R.H., E.K., T.K., S.L., H.R., S.O., J.S., M.E.S., A.W., M.Z., and K.A. were involved in the data acquisition and collection and/or recruited patients. E.K., S.O., H.R., and G.S. were principal investigators at contributing sites. G.S., J.S., M.S., and M.E.S. were involved in the provision of resources and funding acquisition. H.K., S.B., P.F., A.H., L.H., R.H., S.H., S.L., H.R., J.S., M.S., M.E.S., A.W., K.A., and N.E.B. were involved in data analysis and interpretation. H.K., A.H., H.R., J.S., and M.E.S. chnigk and Dr. Al wrote the report. All authors were involved in the critical review of the article and approved the final version.

## Declaration of interests

Dr. Hlinka, Dr. Bornfeld, Mr. Ferenczy, Dr. Gromke, Mr. Grubert, Dr. Held, Dr. Hilger, Dr. Hinke, Dr. Lueong, Dr. Guberina, Dr. Ochsenreither, Dr. Richly, Dr. Scheulen, Dr. Wetter, Dr. Zeschnigk, and Dr. Al-Ghazzawi have nothing to disclose.

Dr. Kalkavan reported grants from AMGEN.

Dr. Bauer reported personal fees from Deciphera, grants from Incyte, grants and personal fees from Blueprint Medicines, personal fees from Lilly, grants and personal fees from Novartis, personal fees from Daichii-Sankyo, personal fees from Plexxikon, personal fees from Exelixis, personal fees from Bayer, and other from Pfizer during the conduct of the study; personal fees from Pharmamar, personal fees from Lilly, personal fees from Roche, and personal fees from GSK outside the submitted work.

Dr. Heinzerling reported grants from Bayer, from null, during the conduct of the study; others from BMS, MSD, Curevac, Roche, Novartis, Amgen, 4SC, Regeneron, grants from Novartis, outside the submitted work.

Dr. Kaempgen reported grants from the BAYER company during the conduct of the study.

Dr. Keilholz reported grants and personal fees from AstraZeneca, grants and personal fees from Boehringer Ingelheim, grants and personal fees from Merck KGaA, personal fees from MSD, personal fees from Novartis, all outside the submitted work.

Dr. G. Schuler reported grants from the BAYER company during the conduct of the study.

Dr. M. Schuler reported personal fees from Amgen, grants and personal fees from AstraZeneca, grants and personal fees from Boehringer Ingelheim, grants and personal fees from Bristol-Myers Squibb, personal fees from Janssen, grants and personal fees from Novartis, personal fees from Roche, personal fees from Takeda, and outside the submitted work.

Dr. Siveke reported personal fees from AstraZeneca, grants and personal fees from Bristol-Myers Squibb, grants and personal fees from Celgene, personal fees from Immunocore, personal fees from Baxalta, personal fees from Aurikamed, personal fees from Falk Foundation, personal fees from iomedico, personal fees from Novartis, grants and personal fees from Roche, personal fees from Shire, other from FAPI holding, other from Pharma15, outside the submitted work.

## STAR★Methods

### Key resources table

**Table:** 

REAGENT or RESOURCE	SOURCE	IDENTIFIER
**Biological samples**		

Human Plasma	This study	N/A
Human tumor tissue	This study	N/A

**Critical commercial assays**		

Maxwell RSC® ccfDNA plasma kit	Promega Corporation, Madison, USA	Cat# AS1480
Droplet Digital PCR Assays for GNAQ p.Q209P c.626A>C GNAQ p.Q209L c.626A>T GNA11 p.Q209L c.626A>T	BIORAD	dHsaCP2506794 dHsaCP2506795 dHsaMDM5827925071 dHsaMDW5827925073 dHsaCP2000049 dHsaMDW8418189543

**Software and algorithms**		

R language		N/A
TESTIMATE (V.6.4)		N/A
NSURV (V. 2.2)		N/A
Graph Pad Prism (V10.3.0)		N/A

### Experimental model and study participant details

#### Patients

Chemonaïve, patients (aged ≥18 years) with metastatic uveal melanoma with at least one measurable metastasis by means of whole-body contrast-enhanced magnetic resonance imaging (MRI) or contrast-enhanced spiral computed tomography (CT) according to RECIST guidelines version 1.1^39^ were eligible. Additional inclusion criteria included an Eastern Cooperative Oncology Group (ECOG) performance status of 0, 1 or 2, a life expectancy of at least 12 weeks, and adequate hematologic, hepatic, and renal function. Patients with a history of another malignancy or other serious medical problems, such as cardiovascular disease including any QT prolongation, thromboembolic events within the last 6 months, symptomatic brain tumors, seizure disorder requiring medication, or active infection requiring systemic treatment were excluded.

#### Study design

This randomized placebo-controlled discontinuation study was conducted at three centers from June 2011 until December 2019 in accordance with the standards of each site’s independent ethics committees, the Declaration of Helsinki, and GCP guidelines. Prior to inclusion, all patients provided written informed consent.

The study was performed analogous to the study of sorafenib in patients with metastatic renal cell carcinoma.^40^

Sorafenib (Bayer AG, Leverkusen, Germany) was administered to all patients in a 56-day open-label run-in period in a dose of 400 mg bid. In patients who completed the run-in period, tumor response was classified according to RECIST. Patients with partial response (PR) were continued on sorafenib, patients with progression (PD) were taken off study and patients with stable disease (SD) were randomly assigned to sorafenib (2 × 200 mg bid) or matching placebo in a double-blind fashion without stratification. Patients were evaluated every 8 weeks for efficacy and for safety by alternate visits and telephone interviews or in case of clinical signs of PD or sequelae. Patients with PD after randomization were unblinded, those on placebo were offered cross-over to sorafenib and those on sorafenib discontinued treatment. Treatment was stopped in case of unacceptable toxicities.

### Method details

#### Endpoints and assessments

The primary endpoint was progression-free survival (PFS) defined as the time from randomization until objective radiologic disease progression or death from any cause.

Secondary endpoints included safety, overall survival (OS), PFS of patients crossing over from placebo and the predictive value of plasma-based biomarkers.

Tumor response was assessed by MRI or CT every 8 weeks and independently assessed by radiologists according to RECIST. Adverse events were graded according to the NCI CTCAE, version 4.0.

#### Safety aspects

In case of significant adverse events possibly related to the study medication, dose was reduced to 400 mg once daily (dose level −1) and 400 mg once every other day (dose level −2) for a minimum of 7 days. If toxicity resolved to grade 0 or 1 after dose reduction, patients could return to the original dose level after 28 days. If not, medication had to be interrupted for a minimum of 7 days until toxicity resolved to grade 0 or 1 with dose reduction thereafter.

#### Circulating tumor DNA quantification

Cell-free DNA (cfDNA) was extracted from 1 mL of plasma using the Maxwell RSC ccfDNA plasma kit (Promega Corporation, Madison, USA) according to the manufacturer’s instructions. When the plasma volume was less than 1 mL, the volume used was noted for later calculations. cfDNA was quantified using droplet digital PCR (ddPCR) using the gene-specific mutant and wild type assays for the ^mut^GNAQ and ^mut^GNA11 locus (Bio-Rad, California, USA) as described in the supplemental information. All plasma samples with either the Q209P or Q209L mutations ≥1 copy/mL in the GNAQ gene locus were considered positive for ^mut^GNAQ and all samples with ≥1 Q209L ^mut^GNA11 copy/mL of plasma were considered positive for ^mut^GNA11.

### Quantification and statistical analysis

In order to detect an improvement from an assumed median post-randomization PFS of 2 months under placebo to 3.5 months in the sorafenib arm (HR = 0.57), with a type I error of 0.1 (one sided) and a power of 85%, a total of 69 events were to be observed. Based on a recruitment period of 36 months and a minimum follow-up of 12 months, 70 patients were required, leading to a target enrollment of 80 patients to allow for a drop-out rate of 10% for the per-protocol population.

Statistical calculations were performed using the R language, TESTIMATE (V.6.4), and NSURV (V. 2.2). All time-to-event analyses were performed using the Kaplan-Meier method and the log rank test, with event times counted from randomization in case of treatment group comparisons, while counted from start of induction in case of prognostic factor analyses, the latter being performed on all patients completing the run-in phase or experiencing progression or early deaths during run-in. For hazard ratios and multivariable analysis, Cox models were applied. The latter included all parameters with a univariable *p* ≤ 0.1, and were reduced in a stepwise backward way with the same cut-off. All *p* values presented are two sided.

The results are expressed as medians and single values are illustrated as scatterplots (Figures S2 and S3). N describes the number of patients within a group. Comparisons between groups for lead time analysis has been applied by two-way ANOVA, followed by Šidák’s multiple comparisons test (Figure S2). The data were analyzed using GraphPad Prism software (GraphPad Software, Inc. CA, US). A *p* value <0.05 was considered significant. ns depicts not significant comparisons.

### Additional resources

This study has been registered on ‘‘https://clinicaltrials.gov/,’’ ID: NCT01377025.
