# Supplementary material for: Sorafenib as first-line therapy for metastatic uveal melanoma: A multicenter, placebo-controlled randomized discontinuation study (STREAM)
Source: iScience. 2025 Nov 14;28(12):114045. doi: 10.1016/j.isci.2025.114045 (PMC12682041; doi:10.1016/j.isci.2025.114045)
Supplement: Document S1. Figures S1–S5 and Methods S1 [file mmc1.pdf]

iScience, Volume *28*

Supplemental information

Sorafenib as first-line therapy for metastatic uveal melanoma: A multicenter, placebo-controlled randomized discontinuation study (STREAM)

Halime Kalkavan, Max E. Scheulen, Eckhart Kämpgen, Ulrich Keilholz, Lucie Heinzerling, Smiths S. Lueong, Annalena Hlinka, Tanja Gromke, Sebastian Ochsenreither, Ralf-Axel Hilger, Matthias Grubert, Axel Wetter, Nika Guberina, Michael Zeschnigk, Peter Ferency, Swantje Held, Axel Hinke, Gerold Schuler, Karim Al-Ghazzawi, Norbert Bornfeld, Nikolaos E. Bechrakis, Martin Schuler, Sebastian Bauer, Heike Richly, and Jens T. Siveke

Supplementary Figure 1, related to Figure 1. Pie charts showing localization, size (T stage) and treatment modality of primary tumors from trial patients. Ciliar body (n=36), choroidal (n=52) or circumferential choroidal (n=2) uveal melanoma has been described. Across patients n=7 had T1, n=12 had T2, n=23 had T3 and n=18 had T4 sized tumors according to AJCC 8th edition. Treatment modalities of primary tumors included brachytherapy (n=47), enucleation (n=31), proton therapy (n=9) and irradiation (n=2). Data not shown, if data on primary tumors n.a.

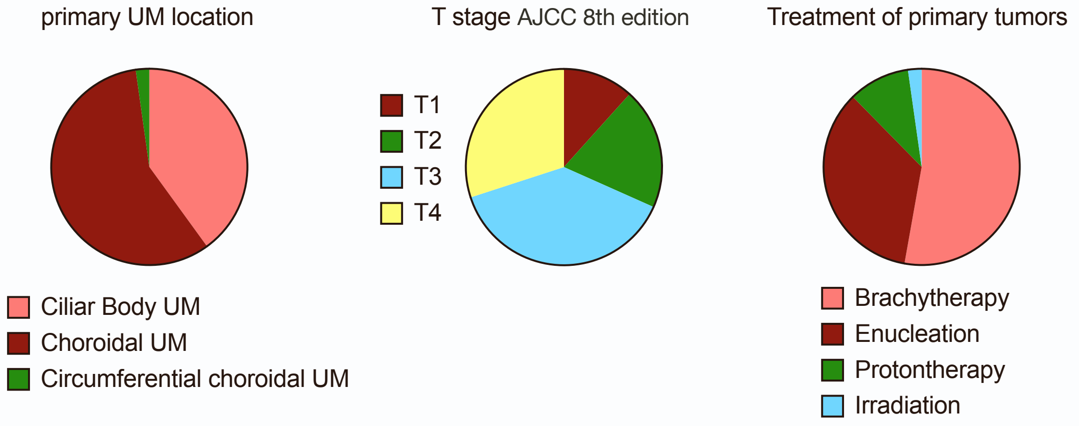

Supplementary Figure 2, related to Figure 1. Lead time between primary uveal melanoma and metastasis.

(A) Lead time between primary uveal melanoma and metastasis in patients randomized into placebo group (n=39), randomized into sorafenib group (n=39), patients that had progressive disease (PD, n=37) during run-in phase or had partial remission (PR, n=2). Data presented as scatter plot of single values with lines at median. 2-way-ANOVA was applied to determine significance, followed by Šidák’s multiple comparisons test. ns, not significant.

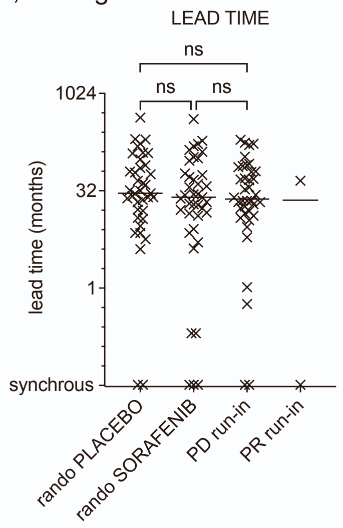

Supplementary Figure 3, related to Figure 1. Time to progression (TTP) of cross-over patients.

Time to progression (TTP) of unblinded, cross-over patients receiving sorafenib. Data presented as scatter plot of single values with lines at median. Statistics are shown in the right panel (values in days).

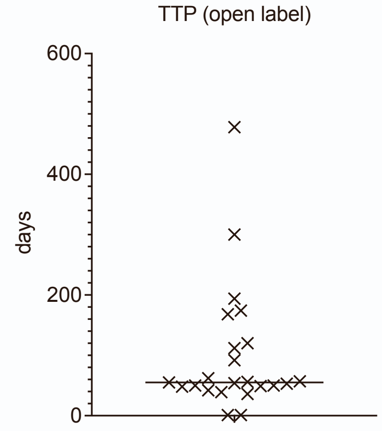

	TTP (open label)
Total number of values	23
Number of excluded values	0
Number of binned values	23
Minimum	1
25% Percentile	48
Median	55
75% Percentile	120
Maximum	478
Mean	99.6086956521739
Std. Deviation	107.680393729202
Std. Error of Mean	22.4529142038796
Lower 95% CI of mean	53.0442015887883
Upper 95% CI of mean	146.17318971556

Supplementary Figure 4, related to Figure 3. Progression-free survival based on labaratory markers. Kaplan-Meier plots of progression-free survival (PFS) stratified by (A) baseline GGT, (B) baseline S100 (C) baseline MIA .

A

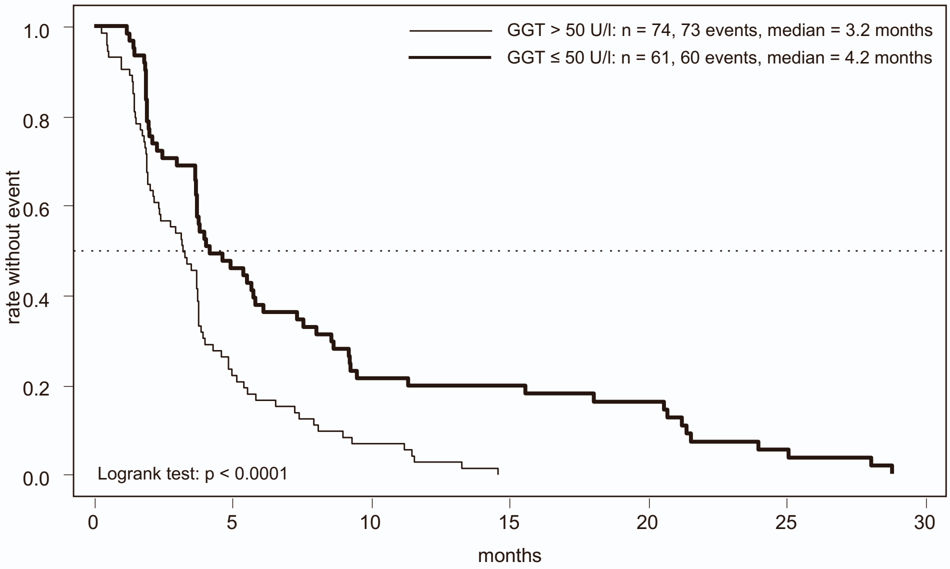

B

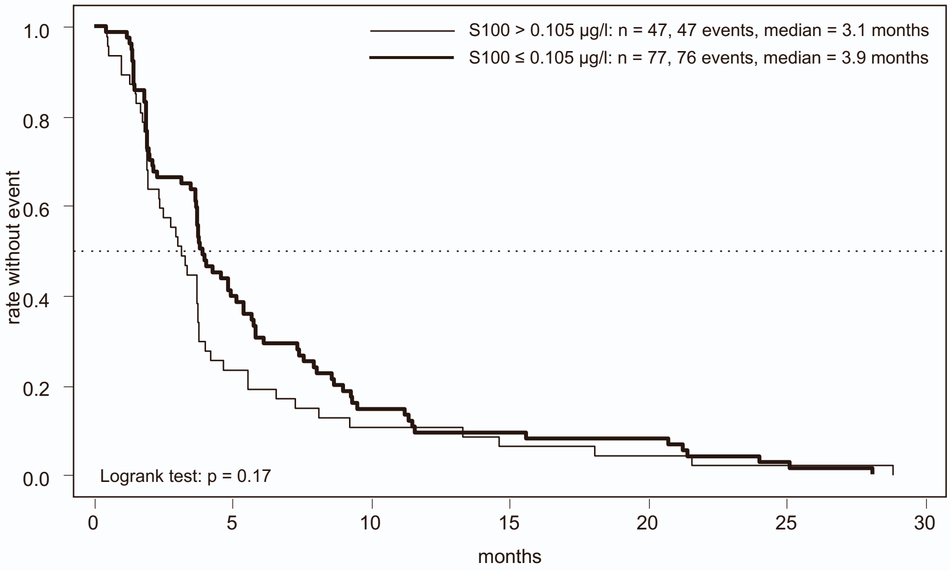

Supplementary Figure 5, related to Figure 3. Overall survival based on laboratory markers.
Kaplan-Meier plots of overall survival rate (OS) stratified by (A) baseline GGT, (B) baseline S100 and (C) baseline MIA.

A

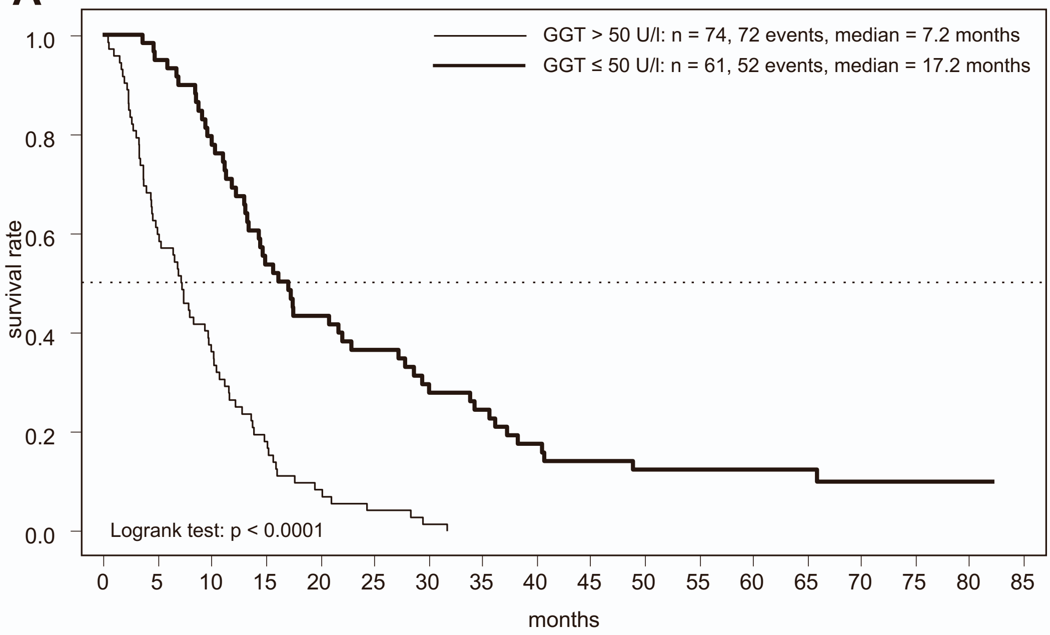

B

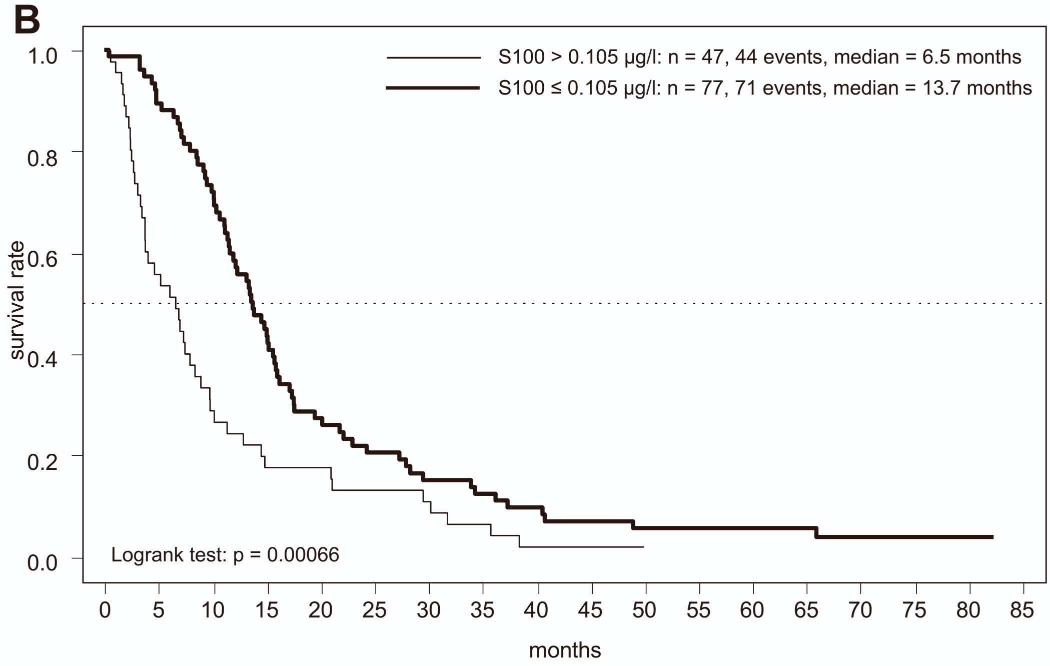

Methods S1: Circulating tumor DNA Quantification

Blood samples were collected in EDTA containing tubes and plasma was prepared by centrifugation at 2500 x g for 10 minutes. The supernatant was then collected and immediately stored at -80°C.

For cfDNA Isolation, plasma samples were allowed to thaw for 1 hour at room temperature and then cleared by centrifugation at 2000 x g for 5 minutes. One milliliter of plasma was loaded on a cartridge of the Maxwell RSC® ccfDNA plasma kit (Promega Corporation, Madison, USA) and cfDNA isolation was automated following pre-installed software on the Maxwell and eluted in 50 µl of elution buffer. When the plasma volume was less than 1 ml, the volume used was noted for later calculations.

Circulating levels of ^{mut}GNAQ and ^{mut}GNA11 were measured by dd-PCR using the gene-specific mutant and wild type assays for each locus (Bio-Rad, California, USA). Using 5 µl of cfDNA, from each sample, the reaction mixtures was assembled in a total volume of 22 µl in a semi-skirted clear wall 96-well plate. The reaction mixture was briefly vortexed and centrifuged to collect liquid at the bottom. All reactions were performed in duplicates and 20 µl of the reaction mixture was used for droplet generation in a QX100™/QX200™ droplet generator (Bio-Rad). All PCR reactions were performed in a C1000 Touch™ thermocycler (Bio-rad) and droplet were read in a QX100™/QX200™ droplet reader (Bio-Rad).

Samples were included only if they had more than 10.000 droplets, otherwise they were repeated. For graphical representation and statistical analysis, a value of 0.5 copies / 20 µl reaction was added to all samples. To make sure that the absolute ctDNA concentration reflected the DNA concentration in the samples, we performed a correlation analysis between DNA sample concentration and copies of ^{mut}GNAQ and ^{mut}GNA11 in tumor tissue-derived DNA samples with known GNAQ and GNA11 mutations respectively. The absolute copy number for each mutant allele was calculated using the formula:

^{mut}GNAQ/GNA11 copies/ml = C+0.5*EV/TV/PV

where C = ^{mut}GNAQ/GNA11 concentration in 20 µl reaction

EV = Total volume in which the cfDNA was eluted

TV = total volume of cfDNA used in the ddPCR reaction

and PV = Total plasma volume used for isolation of cfDNA

A Randomized Discontinuation, Blinded, Placebo-Controlled, Phase II Study of Sorafenib in Patients with Chemonaïve Metastatic Uveal Melanoma

(Sorafenib Treatment of Metastatic Uveal Melanoma)

STREAM

**Study drugs:**	**Sorafenib**
**Protocol Code:**	**STREAM**
**EudraCT-No.:**	**2010-022687-12**
**Final Version 5.0**	**31-July-2013**
**Amendment:**	**Amendment 1 of 31-July-2013**

CONFIDENTIAL

The information contained in this document is regarded as confidential and, except to the extent necessary to obtain informed consent, may not be disclosed to another party unless law or regulations require such disclosure. Persons to whom the information is disclosed must be informed that the information is confidential and may not be further disclosed by them.

**Responsibilities**	
**Sponsor**	Universitätsklinikum Essen<br>Hufelandstr. 55<br>45122 Essen<br>Germany<br>Phone: +49 201 723-0
**Co-ordinating Investigator**	Dr. med. Heike Richly<br>Universitätsklinikum Essen<br>Innere Klinik und Poliklinik (Tumorforschung)<br>Hufelandstr. 55<br>45122 Essen<br>Phone: +49 201 723-85070<br>Fax: +49 201 723-5518<br>E-mail:heike.richly@uk-essen.de
**Data Management**	Clin*Assess* GmbH<br>Birkenbergstr. 82<br>51379 Leverkusen, Germany<br>Phone: +49 2171 36 336 -0<br>Fax: +49 2171 36 336-55<br>E-mail: info@clinassess.de
**Monitoring**	Clin*Assess* GmbH (details see above)
**Statistical analysis**	Clin*Assess* GmbH (details see above)
**Central pharmacy**	Universitätsklinikum Carl Gustav Carus an der Technischen Universität Dresden<br>Klinik-Apotheke<br>Fetscherstr. 74<br>01307 Dresden<br>Contact person: Dr. rer. nat. Ina-Maria Klut<br>Phone: +49 351 458-4625<br>Fax: + 49 351 458-6385
**Central laboratory for determination of S100**	Zentrallabor<br>Universitätsklinikum Essen<br>Hufelandstr. 55<br>45122 Essen

**Central laboratory for determination of MIA**	Dr. Uwe Koch<br>Labor der Hautklinik<br>Universitätsklinikum Erlangen<br>Hartmannstr. 14<br>91052 Erlangen
**Translational research: Pharmacokinetic, ERK-phosphorylation in peripheral blood lymphocytes,<br>tumor marker sVEGF-R2**	Dr. rer. nat. Ralf Axel Hilger<br>Pharmakologie neoplastischer Substanzen<br>Universitätsklinikum Essen<br>Innere Klinik & Poliklinik (Tumorforschung)<br>GBK-Haus, 1 Etage<br>Hufelandstr. 55<br>45122 Essen
**Translational research: Proteomics (MALDI-MS)**	Biodesix Inc.<br>Heinrich Roder<br>520 Zhang Street<br>Suite 213<br>Broomfield<br>Colorado 80021<br>USA<br>Phone: 001-9708709041<br>E-mail: hroder@biodesix.com
**Translational research: GNAQ-mutations**	Dr. med. Claudia Metz<br>Abteilung für Erkrankungen des hinteren Augenabschnitts<br>Universitätsklinikum Essen<br>Hufelandstr. 55<br>45122 Essen
**Translational Research in tumor tissue:<br>p-ERK and p-p70S6k, proteoglycans decorin and versican (immunohisto-chemical staining); gene expression of VEGF-receptor 2**	Prof. Dr. med. Hideo Andreas Baba<br>Institut für Pathologie und Neuropathologie<br>Hufelandstr. 55<br>45122 Essen

**Translational Research in tumor tissue:**<br><br>**MELC (multi-epitope ligand cartography) for in situ tumor fingerprinting**	Prof. Dr. Andreas Baur<br>Toponome Research Facility<br>Universitätsklinikum Erlangen<br>Hartmannstr. 14<br>91052 Erlangen

Signature Page

By my signature, I agree to supervise the conduct of this study and to ensure its conduct in compliance with the protocol, informed consent, the Declaration of Helsinki, ICH Good Clinical Practices guidelines, AMG and GCP-V and other applicable German and European regulations governing the conduct of clinical studies.

Sponsor:

Universitätsklinikum Essen

Represented by Frau Dr. Heike Richly

Hufelandstr. 55

45122 Essen

Germany

Signature of sponsor representative	Place and date
Printed name of sponsor representative	

Signature Page

By my signature, I agree to personally supervise the conduct of this study and to ensure its conduct in compliance with the protocol, informed consent, the Declaration of Helsinki, ICH Good Clinical Practices guidelines, AMG and GCP-V and other applicable German and European regulations governing the conduct of clinical studies.

Co-ordinating investigator:

Dr. med. Heike Richly
Universitätsklinikum Essen
Innere Klinik und Poliklinik (Tumorforschung)
Hufelandstr. 55
45122 Essen
Germany

Signature of co-ordinating investigator	Place and date

Signature Page

By my signature, I agree to personally supervise the conduct of this study and to ensure its conduct in compliance with the protocol and informed consent, the Declaration of Helsinki, ICH Good Clinical Practices guidelines, AMG, GCP-V and other applicable German and European regulations governing the conduct of clinical studies.

Principal investigator:

Printed name of principal investigator and trial site or stamp	
Signature of principal investigator	Place and date

Table of Contents

List of abbreviations 11
1.0 Synopsis 14
2.0 Schedule of study assessments 22
3.0 Background and rationale 24
3.1 Introduction: uveal melanoma 24
3.2 Treatment of metastatic uveal melanoma 25
3.2 Sorafenib 26
3.2.1 Praeclinical and clinical properties 26
3.2.2 Safety experience 27
3.3 Rationale for treatment of uveal melanoma with sorafenib 29
3.4 Rationale for selection of a randomized discontinuation trial design 30
3.5 Risk-benefit assessment of the treatment of patients with metastatic uveal melanoma with sorafenib 31
4.0 Study objectives and endpoints 32
4.1 Study objectives 32
4.1.1 Primary objective 32
4.1.2 Secondary objectives 32
4.2 Study endpoints 33
4.2.1 Primary endpoint 33
4.2.2 Secondary endpoints 33
5.0 Experimental plan 34
5.1 Overall study design 34
5.2 Time schedule 36
5.3 Number of patients and centers 36
5.4 Patient screening and eligibility 37
5.4.1 Patient recruitment 37
5.4.2 Patient randomization 38
5.4.3 Inclusion criteria 38
5.4.4 Exclusion criteria 40
5.5 Study procedures 42
5.5.1 Screening procedures 42
5.5.2 Study visit Day 14 ± 2 days 44
5.5.3 Telephone contact Day 28 ± 2 days 45
5.5.4 Study visit after run-in phase 45
5.5.5 Study visits during continued open label treatment with sorafenib and during randomization phase 47
5.5.6 End of study treatment visit 48
5.5.7 Follow-up 50
5.6 Treatment plan 50
5.6.1 Run-in phase (8 weeks) 50
5.6.2 Continued open-label treatment 50
5.6.3 Randomization phase 51
6.0 Efficacy assessments 52
6.1 Magnetic resonance imaging 52
6.2 Dynamic contrast enhanced liver ultrasound 53
6.3 Tumor markers 53
7.0 Translational research 55
7.1 Tumor marker VEGF-R2 55
7.2 Pharmacokinetic and ERK-phosphorylation in blood lymphocytes 55

7.3 Proteomics 56
7.4 Identification of biomarker in circulating tumor cells 56
7.5 Tumor-specific GNAQ-mutations in serum 58
7.6 Translational research investigations in tumor tissue 58
8.0 Study drug 60
8.1 General information 60
8.2 Formulation 61
8.3 Processing, packaging and labeling 61
8.4 Randomization and distribution of study medication 62
8.5 Storage of study medication 63
9.0 Dose modifications of study medication 63
9.1 Dose reduction levels 63
9.2 Management of treatment-associated skin toxicity 64
9.3 Management of treatment-associated hypertension 67
9.4 Management of treatment-associated diarrhea 68
9.5 Management of treatment-associated hematological toxicities 69
9.6 Management of treatment-associated non-hematological toxicities 69
10.0 Concomitant medications 70
11.0 Study discontinuation 72
11.1 Premature discontinuation of a single patient from study therapy 72
11.2 Premature discontinuation of a single trial centre 73
11.3 Premature discontinuation of the whole trial 73
12.0 Biostatistical analysis 74
12.1 Overview 74
12.2 Patient populations to be analyzed 74
12.3 Statistical methodology 75
12.4 Safety evaluation 77
12.5 Sample size and power considerations 78
13.0 Safety data collection, recording and reporting 78
13.1 Definition adverse event 78
13.2 Definition adverse drug reaction 78
13.3 Definition serious adverse event 79
13.4 Definition suspected unexpected serious adverse reaction 80
13.5 Reporting procedures for all adverse events 80
13.6 Reporting procedures serious adverse events 81
13.7 Sponsor's reporting responsibilities 83
13.8 Pregnancies 83
14.0 Data handling and quality control 84
14.1 Data recording and reporting 84
14.2 Data collection 84
14.3 Study monitoring 84
15.0 Regulatory considerations 84
15.1 Subject insurance 84
15.2 Approval by the competent authorities and ethics committee 84
15.3 Informed consent 85
16.0 Subject confidentiality and data protection 86
17.0 References 86
18.0 Appendices 92
Appendix I: ECOG Performance Status 92
Appendix II: New York Heart Association Functional Classification 93
Appendix III: Pharmacokinetic and pharmacodynamic sample collection and handling 94

Appendix IV: Identification of biomarkers in circulating tumor cells 96
Appendix V: Specifications for whole body MRI in combination with whole body DWI 98
Appendix VI: Tumor fingerprinting by MELC 99

List of abbreviations

5-FU	5-Fluorouracil
AR	Adverse drug reaction
AE	Adverse event
ALT/GPT	Alanine aminotransferase/serum glutamic pyruvic transaminase
AMG	Arzneimittelgesetz (German Drug Law)
ANC	Acute neutrophil count
ASS	Acetylsalicylic acid
AST/GOT	Aspartate aminotransferase/ serum glutamic oxaloacetic transaminase
BID	Twice (two times) a day, “bis in die”
BP	Blood pressure
CAD	Coronary artery disease
CBC	Complete blood count
CR	Complete response
CRA	Clinical research associate
CRF	Case report form
CRO	Contract research organization
CRP	C-reactive protein
CT	Computed tomography
CTC	Circulating tumor cells
DCE-US	Dynamic contrast-enhanced ultrasound
DCR	Disease control rate
DWI	Diffusion weighted imaging
ECG	Electrocardiogram
ECOG	Eastern Cooperative Oncology Group

EMEA; EMA	European Medicines Agency
ERK	Extracellular Signal-Regulated Kinase
FGF-2	Basic fibroblast growth factor
FPFV	First Patient First Visit
GCP	Good clinical practice
GMP	Good manufacturing practice
HFSR	Hand-foot skin reaction
HIV	Human immunodeficiency virus
ICH	International Conference on Harmonization of Technical Requirements for Registration of Pharmaceuticals for Human Use
TLR	Toll -like receptor
LPLV	Last patient last visit
MALDI-MS	Matrix-assisted laser desorption/ionization mass spectrometry
MAP	Mitogen-activated protein
MELC	Multi-epitope ligand cartography
MIA	Melanoma inhibitory activity
MLPA	Multiplex ligation-dependent probe amplification
MRI	Magnetic resonance imaging
NCI-CTCAE	National Cancer Institute Common Terminology Criteria for Adverse Events
NYHA	New York Heart Association
OS	Overall survival
PCR	Polymerase chain reaction
PDGFR	Platelet-derived growth factor receptor
p-ERK	Phosphorylated extracellular signal-regulated kinase
PFS	Progression free survival
PI3K	Phosphatidylinositol 3-kinase

PO	Oral use, “per os”
PR	Partial response
PT/INR	Prothrombin time/international normalized ratio
PTT	Partial thromboplastin time
RDT	Randomized discontinuation trial
RECIST	Response Evaluation Criteria in Solid Tumors
RR	Response rate
RTKs	Receptor tyrosine kinases
RT-PCR	Reverse transcriptase-polymerase chain reaction
SAE	Serious Adverse Event
SAP	Statistical analysis plan
SD	Stable disease
SGOT	Serum glutamic oxaloacetic transaminase
SGPT	Serum glutamic pyruvate transaminase
SmPC	Summary of Product Characteristics
SNP	Single-nucleotide polymorphism
SUSAR	Suspected unexpected serious adverse event
S100	Protein S100
TEAE	Treatment-emergent adverse event
TKI	Tyrosine kinase inhibitor
TNF	Tumor necrosis factor
TTP	Time to progression
ULN	Upper limit of normal
sVEGF-R2	Soluble vascular endothelial growth factor receptor 2

1.0 Synopsis

**Title of Study**	A randomized discontinuation, blinded, placebo-controlled, phase II study of sorafenib in patients with chemonaïve metastatic uveal melanoma (Sorafenib Treatment of Metastatc Uveal Melanoma) **STREAM**
**Indication**	Metastatic uveal melanoma
**Co-ordinating investigator**	Dr. med. Heike Richly
**-Background and Rationale**	Uveal melanoma is the most common primary intra-ocular malignancy in adults with an incidence of 0.6 - 0.7 per 100,000 per year. Uveal melanoma is significantly different from cutaneous melanoma in terms of clinical manifestation and course of disease, pathohistology, molecular profiling and genetics. Thus, in contrast to cutaneous melanoma the liver is the predominant metastatic site in uveal melanoma.<br>Prognosis of metastatic uveal melanoma is poor. In retrospective analyses a median survival time after detection of metastases of 5 months (Flaherty et al, 1998) and 7 months (Kath et al, 1993) was reported. For patients receiving no treatment reported median survival was 2.0 months compared with 5.2 months for those receiving treatment for metastases (Gragoudas et al, 1991).<br>Up to now there is no established treatment of metastatic uveal melanoma. Some therapeutic approaches with locoregional treatment or systemic chemotherapy have been undertaken:<br>In case of metastatic disease which is confined to the liver in about 85% of patients with uveal melanoma surgical resection led to a median survival of 14 months (Mariani et al, 2009) or 19 months and a 5-year survival rate of 22% in a selected patient population (Adam et al, 2006).<br>As locoregional treatment option treatment with fotemustine via direct intra-arterial hepatic infusion was investigated and led to a median survival of 15 months (Peters et al, 2006). This was not a randomized trial, but a report on 101 consecutive treated patients. Additional debulking surgery was performed whenever feasible.<br>Several approaches with systemic chemotherapy in patients with metastatic uveal melanoma were undertaken, mostly with treosulfan, gemcitabine, cisplatin and fotemustine, but did not produce a significant increase in overall survival.<br>A randomized phase III trial comparing intra-arterial hepatic fotemustine administration with intravenous systemic fotemustine and overall survival as primary endpoint is still ongoing (EORTC 18021).<br>Thus, no systemic chemotherapy is approved for metastatic uveal melanoma.<br>Improved understanding of the molecular pathogenesis of cancers has led to a new generation of therapeutic agents that interfere with a specific pathway critical in tumor development or progression. Although no specific genes have been linked to the pathogenesis of uveal melanoma, preclinical studies suggest potential benefit of

	inhibitors of Bcl-2, ubiquitin-proteasome, histone deactylase, mitogen-activated protein kinase and phosphatidylinositol-3-kinase-AKT pathways, and receptor tyrosine kinases. Modifiers of adhesion molecules, matrix metalloproteinase, and angiogenic factors also have demonstrated potential benefit. (Triozzi et al, 2008).<br>Thus, sorafenib as inhibitor of b-Raf and Raf-1 (c-Raf or c-Raf-1), pro-angiogenic vascular endothelial growth factor receptor (VEGFR), and platelet-derived growth factor receptor (PDGFR) may potentially lead to a benefit for patients with metastatic uveal melanoma in terms of disease control and prolongation of survival.<br>In a GCP-adapted register trial 62 patients with metastatic uveal melanoma received treatment with sorafenib on a compassionate use basis. Median overall survival was 10.8 months in patients receiving 200 mg bid sorafenib and 7.1 months in patients receiving 400 mg bid (Scheulen et al, 2011). These treatment results are encouraging for further investigation of treatment with sorafenib in patients with metastatic uveal melanoma in a randomized trial, a potential benefit of this systemic treatment is anticipated.
**Primary objective**	To determine progression free survival (PFS) of sorafenib versus placebo after random assignment (randomized subset only).
**Secondary objectives**	• To compare safety and tolerability in randomization phase<br>The following secondary objectives refer to all patients enrolled:<br>• To determine median overall survival<br>• To determine disease control rate (DCR)<br>• To determine overall PFS and time to progression (TTP)<br>• To determine response rate<br>• To determine whether tumor markers correlate with clinical benefit and whether tumor markers in run-in phase predict clinical benefit<br>• To determine PFS and TTP after unblinding and retreatment with sorafenib (only in subjects randomized to placebo and retreated with sorafenib)<br>• Safety and tolerability
**Primary endpoint**	PFS under treatment with sorafenib versus treatment with placebo after random assignment to blinded study medication in the randomized subset (tumor assessment according to RECIST version 1.1 criteria)
**Secondary endpoints**	**Safety variables:**<br>• Type, incidence and severity of adverse events in the verum and the placebo group in the randomization phase<br>• Type, incidence and severity of adverse events in all patients<br>**Efficacy variables:**<br>• Overall PFS (tumor assessment according to RECIST version 1.1 criteria)

	• Overall survival<br>• Overall response rate and DCR (tumor assessment according to RECIST version 1.1 criteria)<br>• PFS and TTP after unblinding and retreatment with sorafenib (only in subjects randomized to placebo and retreated with sorafenib evaluation of progression, referring to the last tumor assessment before unblinding as 'baseline' value)
**Study design**	Prospective, multicenter, discontinuation phase II trial, open label in the run-in phase and randomized, double-blind, placebo-controlled in the randomization phase
**Study population**	Approximately 200 eligible patients with histologically or cytologically proven and metastatic uveal melanoma will enter the 2 months run-in-phase to randomize 78 stable patients. About 10% of all eligible patients will be expected to have CR/PR and continue sorafenib in the non-randomized part of this trial. Approximately 50 % of all patients who enter the 2 months run-in-phase will be expected to be progressive and stop sorafenib treatment.<br><br>The planned number of randomized patients will be 35 per randomization arm. With an estimated drop-out rate of about 10% the number to be included will be 39 patients per randomization arm (78 patients in total).<br>Enrollment will be continued until a minimum of 78 patients will have been randomized after the run-in phase.
**Inclusion criteria**	1. Signed and dated written informed consent before the start of specific protocol procedures<br>2. Metastatic uveal melanoma with histological or cytological confirmation of liver metastasis (histological or cytological confirmation in case of only extrahepatic metastasis not required for inclusion)<br>3. By means of whole-body MRI documented disease according to RECIST version 1.1 with at least one unidimensional measurable lesion ≥ 10 mm<br>4. Eastern Cooperative Oncology Group (ECOG) performance status of 0, 1, 2<br>5. Male or female patients ≥ 18 years of age<br>6. Estimated life-expectancy more than 5 months<br>7. Hematologic function, as follows:<br>– Absolute neutrophil count (ANC) ≥ 1.5 x 10^9/L<br>– Platelet count ≥ 100 x 10^9/L<br>– Hemoglobin ≥ 9 g/dL<br>8. Renal function, as follows

	– Creatinine ≤ 1.5 x upper limit of normal (ULN)<br>9. Hepatic function, as follows<br>– Aspartate aminotransferase (AST) ≤ 2.5 x ULN (if liver metastases ≤ 5 x ULN)<br>– Alanine aminotransferase (ALT) ≤ 2.5 x ULN (if liver metastases ≤ 5 x ULN)<br>– Total bilirubin ≤ 3 mg/dl<br>– Alkaline phosphatase ≤ 4.0 x ULN<br>10. PT-INR/PT < 1.5 x ULN<br>11. Females of childbearing potential (FCBP) must have a negative pregnancy test within 7 days of the first application of study treatment<br><br>**and**<br><br>must agree to use effective contraceptive birth control measures (combined oral contraceptives, hormone-releasing intrauterine contraceptive device, hormonal contraceptive implants, hormonal contraceptive injectables) in combination with barrier birth control measures during the course of the trial<br>**or** be surgically sterile<br><br>A female subject is considered to be of childbearing potential unless she is age ≥ 50 years and naturally amenorrhoeic for ≥ 2 year, or unless she is surgically sterile.<br>12. Males must agree to use barrier birth control measures (condomes) during the course of the trial. In addition males must agree to continue to use these barrier birth control measures for at least 3 months after last administration of study medication.
**Exclusion criteria**	1. Previous or concurrent tumor other than uveal melanoma with the exception of cervical cancer in situ, adaequately treated basal cell carcinoma, superficial bladder tumors (Ta, Tis, and T1) or any curatively treated tumors > 3 years prior to enrollment<br>2. History of cardiac disease: congestive heart failure ≥ New York Heart Association (NYHA) class 2; active coronary artery disease ([CAD], myocardial infarction more than 6 months prior to study entry is allowed), cardiac arrhythmias requiring antiarrhythmic therapy (only beta blockers or digoxin are permitted)<br>3. QT/QTc-interval prolongation (QTc> 450 ms) on ECG, known Long QT syndrome or known Long QT syndrome in relatives<br>4. Known HIV infection

	5. Known chronic infection with hepatitis B or C<br>6. Hypokalemia, hypocalcemia, hypomagnesemia or patients under actual treatment against hypokalemia, hypocalcemia, hypomagnesemia<br>7. Active infection requiring systemic antibiotic/antiviral/antifungal treatment or any uncontrolled infection > Grade 2 NCI-CTCAE<br>8. Symptomatic brain or meningeal tumors (unless patient is > 6 months from definitive therapy, had a negative imaging study within 4 weeks of study entry and is clinically stable with respect to the tumor at the time of study enrollment)<br>9. Patients with seizure disorder requiring medication (such as steroids or antiepileptics)<br>10. History of organ allograft<br>11. Patients with evidence or history of bleeding diathesis<br>12. Thrombotic or embolic events within the last 6 months<br>13. Serious non-healing wound, ulcer or fracture<br>14. Uncontrolled arterial hypertension with systolic blood pressure >150 mm Hg and/ or diastolic blood pressure > 90 mg Hg despite optimal treatment, determined twice within one week<br>15. Pregnant or breast-feeding patients<br>16. Marked claustrophobia<br>17. Cardiac pacemaker, cochlea implants or other implanted metal devices, residual metal splinters<br>18. Known allergy to the used study drug sorafenib or to any of its excipients<br>19. Known hypersensitivity to gadolinium based contrast agents<br>20. Subject unwilling or unable to comply with study requirements<br>21. Substance abuse, medical, psychological or social conditions that may interfere with the patient´s participation in the study or evaluation of the study results<br>22. Participation in any clinical study or treatment with an experimental drug or experimental therapy within 28 days prior to study enrollment or during study participation<br>23. Patients receiving anticoagulation therapy with warfarin or phenprocoumon<br>24. Treatment with any of the following therapies or drugs<br>– Any prior palliative chemotherapy, tyrosine kinase inhibitors (TKI´s) or antiangiogenics (prior **adjuvant** treatment with vaccine or immunotherapy is allowed provided there is documentation of disease progression)<br>– Any chemotherapy, hormonal therapy, immunotherapy, targeted therapy or experimental or approved

	proteins/antibodies within four weeks prior to study enrollment or during study participation<br>– Radiotherapy or brachytherapy within four weeks prior to study enrollment or during study participation except to eye or bone<br>– Hepatic chemoembolization within four weeks prior to study enrollment or during study participation:<br>– Major surgery within 4 weeks of study enrollment<br>– Autologous bone marrow transplant or stem cell rescue within 4 months of study enrollment<br>– Use of biologic response modifiers, such as G-CSF, within 3 week of study enrollment. (G-CSF and other hematopoietic growth factors may be used in the management of acute toxicity such as febrile neutropenia when clinically indicated or at the discretion of the investigator; however, they may not be substituted for a required dose reduction.)<br>– Patients receiving newly initiated treatment with erythropoietin or dose adjusted treatment with erythropoietin within 8 weeks of study enrollment or during study participation (Patients receiving permanent erythropoietin treatment are permitted provided no dose adjustment was undertaken within 2 months prior to the study or during the study).<br>– Intake of any drug that could cause QT-interval prolongation during the four weeks prior to study enrollment and during study treatment<br>– Any St. John's wort containing remedy<br>– Prior exposure to study drug<br>– Any prior therapy with high cumulative dose of anthracyclines
**Treatment regimen**	In an initial 8-week open-label run-in period sorafenib will be administered to all patients at a dose of 400 mg bid orally each day.<br>After 8 weeks response status will be assessed according to RECIST version 1.1 criteria. Patients with complete response (CR) and partial response (PR) according to RECIST version 1.1 (≥ 30% decrease in the sum of diameters of target lesions, taking as reference the baseline sum diameters, no evidence of new metastases) will continue to receive sorafenib (400 mg bid or reduced dose level according to the dose modifications schedule in case of toxicity) until disease progression or unacceptable toxicity. Patients with progressive disease (PD) (≥ 20% increase in the sum of diameters of target lesions, taking as reference the smallest sum on study with an absolute increase of at least 5 mm or other evidence of progression such as one or more new lesion) will discontinue sorafenib treatment.

	Patients with stable disease ([SD]; less than 30% decrease in the sum of diameters of target lesions, taking as reference the baseline sum diameters and without progression) will be randomly assigned to either sorafenib 400 mg bid orally or matching placebo in a double-blind fashion. However, patients, who received a reduced dose level of sorafenib in the run-in phase because of any toxicity as required according to the dose modifications schedule in Section 9.0, will continue to receive this dose level after randomization to sorafenib or placebo (i.e instead of 400 mg bid sorafenib or 400 mg bid placebo the respective dose level will apply for the dose of sorafenib and placebo). Blinded treatment will be continued until PD (progression according to RECIST) or unacceptable toxicity occurs. For patients who experience a progression at any time during the randomization phase, the blind should be broken. Patients having received placebo during the randomization phase should be offered continuing treatment with sorafenib 400 mg bid.<br>Doses of sorafenib will be reduced or delayed according to dose reduction and dose modifications schedules in any treatment phase if clinically significant toxicities considered related to sorafenib occur.
**Statistical and analytical plan and methodology**	All patients who receive at least one dose of study medication (sorafenib as run-in medication, open label sorafenib, randomized sorafenib or placebo) will be included in the *Full analysis population* being identical with the *Safety population*. This patient set will be used for most secondary efficacy parameters and all safety parameters to be evaluated. Data will be summarized for the following treatment groups: “Open sorafenib continued after run-in”, “No study treatment after run-in”, “Blinded sorafenib after run-in”, “Blinded placebo after run-in”, and “All patients treated with sorafenib”.<br>The *Randomized population* will include only patients being randomized after the 8-week run-in sorafenib treatment. It is the primary population for the primary efficacy parameter. Data will be summarized for the following treatment groups: “Blinded sorafenib after run-in” and “Blinded placebo after run-in”. If regarded necessary prior to database closure, a per-protocol analysis for the *Per-protocol population* including only eligible patients may be performed. Criteria for eligibility will be set up in the Statistical Analysis Plan (SAP) detailing the statistical evaluation.<br>To evaluate the secondary efficacy parameter ‘PFS and TTP after unblinding and retreatment with sorafenib’ the *Placebo to sorafenib population* will be defined as subset of all patients of the Randomized population who are randomized to placebo and retreated with open sorafenib follow-up medication after unblinding. Appropriate subgroups may be defined in the SAP.<br>The primary endpoint is progression free survival (PFS) according to RECIST version 1.1 criteria in the randomized subset or death (for those who die prior to documented progression). Patients alive without documented progression at the time of analysis will be censored at their last date of tumor evaluation.<br>The analysis will not be done before 69 events are observed within the *Randomized population*. The two treatment groups will be compared using a one-sided log-rank test with an alpha of 0.1.

	Kaplan-Meier estimates and survival curves will be presented for each treatment group, as well as the hazard ratio with its confidence interval. Goal of the study is to show higher PFS for patients receiving sorafenib than for patients receiving placebo.<br>To prevent patient's unnecessary exposure to placebo an interim analysis will be performed after about half the patients are evaluable with regard to PFS after randomisation.<br><br>The Safety population will be the primary population for evaluating administration/compliance and safety. Descriptive summary tables will be presented on all safety parameters by treatment group (randomized patient population and non-randomized patients). Patients will be monitored for adverse events using the National Cancer Institute Common Terminology Criteria for Adverse Events (NCI-CTCAE) version 4.0. Treatment-emergent adverse events (TEAEs) and safety laboratory parameters will be summarized by treatment group and NCI-CTCAE v.4.0 grade.
**Sample size justification/Target number of valid cases**	The sample size for this study is determined based on the assumptions that the median PFS for patients receiving placebo is 2 months. Improvement by 75% to median PFS of 3.5 months in the sorafenib arm is clinically significant. A total of 69 events are required to detect a difference in median PFS of 2 vs 3.5 months (one-sided, $\alpha=0.1$, 85% power). Assuming a recruitment period of 36 months and a follow-up for at least 12 months a total sample size of 70 patients is required. To account for 10% drop-outs 78 patients will be randomized at a minimum. To prevent patient's unnecessary exposure to placebo an interim analysis will be performed after about half the patients are evaluable with regard to PFS after randomisation.
**Estimated number of sites**	3
**Study duration**	Estimated duration of the study will be 5 years (estimated 3 years duration of recruitment, estimated 1 year treatment phase, duration of follow-up in each patient until death or at least one year after last study drug application).<br>Begin of the Study: First patient First visit (FPFV): Date of the written informed consent by the first patient enrolled.<br>End of the study: Last Visit Last Patient (LPLV) will be the last follow-up visit of the last patient having received study drug.

2.0 Schedule of study assessments

PROCEDURES	SCREENING PERIOD	RUN-IN PHASE			RANDOMIZATION PHASE OR CONTINUED OPEN LABEL TREAT-MENT		END OF STUDY TREAT-MENT	FOL-LOW-UP
	**Within Day-7 to -1**	**Day 14 ± 2 days**	**Day 28 ± 2 days**	**Day 56 ± 2 days**	**4 -week interval between regular study visits**	**Every 8 weeks ± 3 days and if clinically indicated**	**Within 2 weeks after end of treat-ment**	**Every 8 weeks**
Medical history including demography, pre-existing conditions and previous medication	X							
Record medical history and prior treatment of uveal melanoma	X							
Vital signs including blood pressure /height and weight[1]	X	X		X		X	X	
Physical examination	X	X		X		X	X	
Verification of inclusion-/exclusion criteria	X							
ECOG	X	X		X		X	X	
Randomization to blinded study drug (sorafenib or placebo)				X[2]				
**Efficacy assessments**								
MRI including DWI[3]	X[3]			X[3]		X[3,6]	X[6]	
Tumor assessment (RECIST v.1.1)	X			X		X	X	
MRI liver with DWI liver[4]		X[4]						
Contrast enhanced liver ultrasound (DCE-US)[5]	X[5]	X[5]		X[5]		X[5,6]	X[5]	
Tumor marker (S100, MIA)	X	X		X		X[6]	X	
Record anti-tumor therapy								X
Survival status								X
**Optional translational research:**								
Sorafenib plasma concentration/ ERK-phosphorylation in blood lymphocytes	X[7]	X[7]		X[7]				
sVEGF-R2	X	X		X				
Circulating tumor cells-identification of biomarker	X	X		X				
GNAQ-mutations in serum	X	X		X				
Proteomics (MALDI-MS)	X	X		X				

Informed consent for optional translational research in tumor tissue specimen (only if tumor tissue of primary tumor or tumor metastasis is available):	X							
**Safety assessments**								
Information about health condition and possible adverse events via telephone contact investigator/patient			X		X			
Hematology[8]	X	X		X		X	X	
Coagulation (PT-INR, PTT)[9]	X	X		X		X	X	
Clinical serum chermistry[10]	X	X		X		X	X	
Urinalysis[9]	X			X		X	X	
Pregnancy test (only FCBP)[11]	X						X	
ECG	X	X		X		X	X	
Record adverse events		X		X		X	X	
Record concomitant medications and therapy		X		X		X	X	
**Study drug**								
Dispense of study medication[12]	X[12]			X		X[12]		
Return of unused study drug/drug account				X		X	X	

[1] Height has to be determined only once at screening. Vital signs will also be evaluated as safety assessments. Blood pressure has to be recorded at least three times at different times of the day on a single day at screening, at the study visits after 2 and after 8 weeks during the run-in phase, and at least once daily every 8 weeks during the study visits in randomization/ continued open label treatment phase. Patients have to perform self-measurements of the blood pressure at least three times a day during the the eight weeks of the run-in phase and record the blood pressure in a diary.

[2] Only patients with SD in the tumor assessment

[3] Whole body MRI in combination with DWI at screening and at the subsequent examinations in patients with extrahepatic metastasis or if clinically indicated (e.g. suspicion of new extrahepatic metastasis). MRI of the liver in combination with DWI of the liver in patients with exclusively hepatic metastasis at screening and no suspicion of extrahepatic metastasis is sufficient at the subsequent examinations (starting with the study visit at the end of the run-in phase [Day 56]). Patients with exclusively extrahepatic metastasis at screening will undergo further whole body MRI without DWI. If DWI cannot be performed in any trial center for logistical obstacles, MRI examinations without DWI.

[4] Only in patients with measurable liver metastases at the screening MRI-DWI evaluation and in trial centers where DWI is performed.

[5] Only if DCE-US will be performed at trial center and only if the patient had hepatic metastases in the whole body MRI examination at screening. DCE-US will not be utilized for evaluation of response.

[6] Has to be performed every 8 weeks ± 3 days and if clinically indicated, such as in case of suspected clinical or symptomatical progression (before unblinding of randomized subjects).

[7] Has to be performed at screening, on day 14 and day 56 between 11.00 a.m. and 1.00 p.m. The exact date and time of blood sample collection must be recorded. At screening only ERK-phosphorylation in blood lymphocytes will be determined.

[8] Hematology comprises complete blood count (including hematocrit, hemoglobin, reticulocytes, leucocytes, neutrophils and platelets). The complete blood count must be checked within 7 days prior to first application of study drug, after 2 and after 8 weeks during the run-in phase, every 8 weeks during the randomization/ continued open label treatment phase, and whenever clinically indicated as well as within two weeks after end of treatment.

[9] Additional assessments whenever clinically indicated.

[10] Serum chemistry comprises blood glucose, electrolytes (sodium, potassium, chloride, calcium, magnesium), phosphate, creatinine, blood urea, uric acid, ALT/GOT, AST/GPT, gamma- GT, total bilirubin, AP, LDH, CRP, total protein, albumin, amylase, and lipase. Serum chemistry must be checked within 7 days prior to first application of study drug, after 2 and after 8 weeks during the run-in phase, every 8 weeks during the randomization/ continued open label treatment phase, and whenever clinically indicated as well as within two weeks after end of treatment.

[11] Pregnancy testing has to be performed within 7 days of the first application of the study drug, within two weeks after end of treatment and whenever clinically indicated in FCBP. A female subject is considered to be of childbearing potential unless she is age ≥ 50 years and naturally amenorrhoeic for ≥ 2 year or unless she is surgically sterile.

[12] For patients who experience progression at any time during the randomization phase, the blind should be broken. Patients having received placebo should be offered continuing treatment with sorafenib. Patients randomized to sorafenib should be taken off study medication.

[13] Dispense of study drug only after review of in- and exclusion criteria and patient registration.

3.0 Background and rationale

3.1 Introduction: uveal melanoma

Uveal melanoma is the most common primary intra-ocular malignancy in adults. The incidence of uveal melanoma is very low with 0.6-0.7/100,000/year, which stands for about 500 new patients per year in Germany. Uveal melanoma is significantly different from cutaneous melanoma in terms of clinical manifestation and course of disease, pathohistology, molecular profiling and genetics. Thus, in contrast to cutaneous melanoma the liver is the predominant metastatic site in uveal melanoma, where monosomia 3 is significantly correlated with poor prognosis due to the development of liver metastases (Prescher et al, 1996) which is not seen in cutaneous melanoma. Other genetic alterations commonly observed in uveal melanoma are amplification of 8q, whereas deletions and translocations 1p are only infrequently seen, and TP53 mutations, BRAF mutations, CDKN1 mutations and PTEN mutations are rare (Triozzi et al, 2008). In the opposite, the v600e-mutation of BRAF has a frequency of about 60% in cutaneous melanoma and does not occur in uveal melanoma.

The significant impact of monosomia°3 on prognosis by development of metastatic disease predominantly to the liver in uveal melanoma is not yet sufficiently understood. Monosomia°3 may either be important in tumor progression because of the deletion of a specific gene or genes or may only indicate genomic instability. Inactivating somatic mutations were identified in the gene encoding BRCA1-associated protein 1 (*BAP1*) on chromosome 3p21.1 in 26 of 31 (84%) metastatic tumors (Harbour et al, 2010). Thus, loss of *BAP1* may be deleterious in uveal melanoma in terms of metastatic potential especially in case of monosomia 3 with only one susceptible allel.

Although mutations in BRAF are rare in uveal melanoma, wild-type B-Raf seems to play a key role in the proliferation of uveal melanoma by activating ERK. Active phosphorylated ERK has been observed in uveal melanomas in different cellular types in nearly homogeneous intratumoral expression (Weber et al, 2003; Saraiva et al, 2005).

Uveal melanoma cells produce several factors that promote angiogenesis in combination with invasion and metastasis, such as basic fibroblast growth factor (FGF-2) and vascular endothelial growth factor [VEGF] (Ijland et al, 1999).

3.2 Treatment of metastatic uveal melanoma

Prognosis of metastatic uveal melanoma is poor. In retrospective analyses a median survival time after detection of metastases of 5 months (Flaherty at al, 1998) and 7 months (Kath et al, 1993) was reported. For patients receiving no treatment reported survival was 2.0 months compared with 5.2 months for those receiving treatment for metastases (Gragoudas et al, 1991).

Up to now there is no established treatment of metastatic uveal melanoma, partly due to the rarity of the disease and paucity of clinical trials, especially of randomized trials. As consequence patients with metastatic uveal melanomal are often offered individual treatment options. Some therapeutic approaches with locoregional treatment or systemic chemotherapy that have been undertaken and are described in the following passage:

In case of metastatic disease which is confined to the liver in about 85% of patients with uveal melanoma surgical resection led to a median survival of 14 months (Mariani et al, 2009) or 19 months and a 5-year survival rate of 22% in a selected patient population (Adam et al, 2006).

As locoregional treatment option treatment with fotemustine via direct intra-arterial hepatic infusion was investigated and led to a median survival of 15 months (Peters et al, 2006). This was not a randomized trial, but a report on 101 consecutive treated patients. Additional debulking surgery was performed whenever feasible. Improved outcome could have been influenced by patient selection. This fact is reflected by the patient characteristics that revealed an elevated LDH in only 21 of 101 patients, whereas 51 patients showed no elevated LDH, thus indicating probable smaller tumor burden. Further on, intra-arterial infusion possibly does not constitute a treatment option for the majority of patients with disseminated liver metastasis or patients with extrahepatic localisation of metastases. Catheter related complications occurred in 23 % of patients and required treatment discontinuation in 10% (Peters et al, 2006).

A randomized phase III trial comparing intra-arterial hepatic fotemustine administration with intravenous systemic fotemustine and overall survival as primary endpoint is still ongoing (EORTC 18021).

Several approaches with systemic chemotherapy in patients with metastatic uveal melanoma were undertaken, mostly with treosulfan, gemcitabine and fotemustine, but did not produce a significant increase in overall survival.

In a phase II clinical trial of cisplatin, gemcitabine and treosulfan the median overall survival was 7.7 months and median progression free survival (PFS) was 3.0 months (Schmittel et al, 2005).

In a two-cohort phase II clinical trial of gemcitabine plus treosulfan (treosulfan dose in cohort one: 2500 mg/m^2 or 3000 mg/m^2 and in cohort 2 3500 mg/m^2 or 4000 mg/m^2), the median survival time was 6.0 months in cohort 1 versus 9.0 months in cohort 2, respectively (Schmittel et al, 2005).

A randomized phase II trial of gemcitabine plus treosulfan versus treosulfan alone observed PFS of 3 months in the gemcitabine/treosulfan arm and of 2 months in the treosulfan arm. However the study was designed to analyze the rate of objective response in the treatment arms (Schmittel et al, 2006).

Systemic chemotherapy either with sequential fotemustine (either intra-arterial infusion in the hepatic artery or systemic intravenous application), interferon alpha, and interleukin 2 resulted in overall survival of approximately one year (Becker et al., 2002).

In conclusion, overall mortality rate remains high because of the development of metastatic disease, which is highly resistant to systemic therapy (Triozzi et al, 2008; Vahrmeijer et al, 2008). Thus, no systemic chemotherapy is approved for metastatic uveal melanoma.

3.2 Sorafenib

3.2.1 Praeclinical and clinical properties

Sorafenib (Nexavar®) is an oral multi-kinase inhibitor that targets the Raf/MEK/ERK signaling pathway (CRAF, BRAF, V600E BRAF) in the cell and receptor tyrosine kinases (RTKs) such as VEGFR-2, VEGFR-3, and PDGFR-ß involved in tumor cell proliferation and angiogenesis (Wilhelm et al, 2004). *In vitro*, sorafenib inhibited b-Raf and Raf-1 (c-Raf or c-Raf-1), pro-angiogenic vascular endothelial growth factor receptor (VEGFR), and platelet-derived growth factor receptor (PDGFR) (Wilhelm et al, 2002). RAF kinases are serine/threonine kinases, whereas c-KIT, FLT-3, VEGFR-2, VEGFR-3, and PDGFR-ß are receptor tyrosine kinases (Nexavar, SmPC). Sorafenib prevented tumor cell proliferation *in vitro* and tumor growth in human xenograft models, the primary effect of sorafenib was inhibition of tumor growth rather than tumor shrinkage (Wilhelm et al, 2004). Sorafenib inhibited ERK-mediated, uveal melanoma proliferation (Calipel et al, 2006).

The safety and efficacy of sorafenib in the treatment of advanced renal cell carcinoma and in advanced hepatocellular cancer were investigated in clinical studies.

A phase II randomized discontinuation trial evaluated the effects of sorafenib on tumor growth in patients with metastatic renal cell carcinoma. Progression-free survival in patients with renal cell carcinoma was significantly longer in the sorafenib group than in the placebo group.

In a phase III, multi-centre, randomized, double blind, placebo-controlled study in 903 patients with advanced clear cell renal carcinoma and low and intermediate risk progression-free survival was prolonged in patients receiving sorafenib compared to those receiving placebo (Escudier et al, 2006).

In a phase III, international, multi-centre, randomized, double blind, placebo-controlled study in 602 patients with hepatocellular carcinoma median survival and time to progression were nearly 3 months longer for patients treated with sorafenib than for those given placebo (Llovet et al, 2008).

Sorafenib has been approved for the treatment of patients with locally advanced or metastatic renal carcinoma and primary hepatocellular cancer in June 2006 by the EMA (Summary of Product Charateristics [SmPC], Nexavar®).

3.2.2 Safety experience

The following special warnings and precautions for treatment with sorafenib are listed in the Summary of Product Characteristics (SmPD) to Nexavar®.

Dermatological toxicities

Hand-foot skin reaction (palmar-plantar erythrodysaesthesia) and rash represent the most common adverse drug reactions with sorafenib. Rash and hand-foot skin reaction are usually Common Terminology Criteria for Adverse Events (CTCAE) grade 1 and 2 and generally appear during the first six weeks of treatment with sorafenib. Management of dermatological toxicities may include topical therapies for symptomatic relief, temporary treatment interruption and/or dose modification of sorafenib, or in severe or persistent cases, permanent discontinuation of sorafenib and precautions have to be respected (detailed instructions for management of skin toxicity in section 9.2).

Hypertension

An increased incidence of arterial hypertension was observed in sorafenib-treated patients. Hypertension was usually mild to moderate, occurred early in the course of treatment, and was amenable to management with standard antihypertensive therapy. Blood pressure should be monitored regularly and treated, if required, in accordance with standard medical practice.

In cases of severe or persistent hypertension, or hypertensive crisis despite institution of antihypertensive therapy, permanent discontinuation of sorafenib should be considered (detailed instructions for management of hypertension in section 9.3).

Haemorrhage

An increased risk of bleeding may occur following sorafenib administration. If any bleeding event necessitates medical intervention it is recommended that permanent discontinuation of sorafenib should be considered (detailed instructions for management of bleeding ulcer in section 9.1)

Cardiac ischaemia and/or infarction

In randomised, placebo-controlled, double-blind studies the incidence of cardiac ischaemia/infarction events was higher in the sorafenib group compared with the placebo group. Temporary or permanent discontinuation of sorafenib should be considered in patients who develop cardiac ischaemia and/or infarction (detailed instructions for management of non-hematological toxicities in section 9.6).

QT interval prolongation

Nexavar has been shown to prolong the QT/QTc interval, which may lead to an increased risk for ventricular arrhythmias. Sorafenib has to be used with caution in patients who have, or may develop prolongation of QTc, such as patients with a congenital long QT syndrome, patients treated with a high cumulative dose of anthracycline therapy, patients taking certain anti-arrhythmic medicines or other medicinal products that lead to QT prolongation, and those with electrolyte disturbances such as hypokalaemia, hypocalcaemia, or hypomagnesaemia (detailed instructions for management of non-hematological toxicities in section 9.6).

Gastrointestinal perforation

Gastrointestinal perforation is an uncommon event and has been reported in less than 1% of patients taking sorafenib. In some cases this was not associated with apparent intra-abdominal tumor. Sorafenib therapy should be discontinued in case of any gastrointestinal perforation.

Hepatic impairment

Since sorafenib is mainly eliminated via the hepatic route exposure might be increased in patients with severe hepatic impairment.

Warfarin co-administration

Infrequent bleeding events or elevations in the International Normalised Ratio (INR) have been reported in some patients taking warfarin while on sorafenib therapy. Concomitant use of warfarin, phenprocoumon, heparin low-molecular heparinor treatment with ASS > 100 mg daily will not be allowed during study participation (see section 10.0).

Wound healing complications

No formal studies of the effect of sorafenib on wound healing have been conducted. Temporary interruption of sorafenib therapy is recommended for precautionary reasons in patients undergoing major surgical procedures (detailed instructions in case of surgical treatment in section 9.1).

Common adverse events

The most common adverse reactions under treatment with sorafenib were diarrhoea, rash, alopecia and hand-foot syndrome. Other common adverse events occurring in ≥ 10 % of all patients treated were anorexia, fatigue, nausea, vomiting, hypertension, pruritus, dry skin, erythema, fatigue.

In studies with sorafenib increased lipase and amylase were very commonly reported laboratory test abnormalities. CTCAE Grade 3 or 4 lipase and amylase elevations were observed in the sorafenib and the placebo group in more than 1% and less than 11% of the treated patients. Hypophosphataemia was another very common laboratory finding, observed in about 35 % to 45% of sorafenib treated study patients. CTCAE Grade 3 hypophosphataemia (1 – 2 mg/dl) occurred in about 11-13 % of study patients treated with sorafenib. There were no cases of CTCAE Grade 4 hypophosphataemia (< 1 mg/dl) reported. The aetiology of hypophosphataemia associated with sorafenib is not known.

3.3 Rationale for treatment of uveal melanoma with sorafenib

Improved understanding of the molecular pathogenesis of cancers has led to a new generation of therapeutic agents that interfere with a specific pathway critical in tumor development or progression. Although no specific genes have been linked to the pathogenesis of uveal melanoma, which significantly differs from that of cutaneous melanoma, progress has been made in identifying potential targets involved in uveal melanoma apoptosis, proliferation, invasion, metastasis, and angiogenesis. Accordingly, improvement of systemic therapy of metastatic uveal melanoma could be achieved by using molecularly targeted agents that are currently in clinical use as well as agents being tested in clinical trials. Preclinical studies suggest potential benefit of inhibitors of Bcl-2, ubiquitin-proteasome, histone deactylase, mitogen-activated protein kinase and phosphatidylinositol-3-kinase-AKT pathways, and

receptor tyrosine kinases. Modifiers of adhesion molecules, matrix metalloproteinase, and angiogenic factors also have demonstrated potential benefit. (Triozzi et al, 2008).

Thus, sorafenib as oral multi-kinase inhibitor that targets the Raf/MEK/ERK signaling pathway (CRAF, BRAF, V600E BRAF) in the cell and receptor tyrosine kinases (RTKs) such as VEGFR-2, VEGFR-3, and PDGFR-ß involved in tumor cell proliferation and angiogenesis may potentially lead to a benefit for patients with metastatic uveal melanoma in terms of disease control and prolongation of survival.

In a GCP-adapted register trial approved by the ethics committee in Essen 62 patients with metastatic uveal melanoma received treatment with sorafenib on a compassionate use basis. Median overall survival was 10.8 months in patients receiving 200 mg bid sorafenib and 7.1 months in patients receiving 400 mg bid (Scheulen et al, 2011). These treatment results are encouraging for further investigation of treatment with sorafenib in patients with metastatic uveal melanoma in a randomized trial, a potential benefit of this systemic treatment is anticipated.

3.4 Rationale for selection of a randomized discontinuation trial design

The randomized discontinuation trial (RDT) design , first proposed in 1975 (Amery et al, 1975) aims to assess the clinical activity of a drug while minimizing the use of placebo. All patients receive study drug for an initial run-in period, followed by random assignment of potential responders to either the study drug or the placebo (Amery et al,1975; Kopec et al,1993). RDT design provides more homogeneous study treatment groups by selecting patients with a predefined response, and allows the evaluation of a drug's clinical activity with fewer patients and increased statistical power. Thus this study design is especially useful to distinguish anticancer activity of the drug and natural history of the underlying disease (Kopec et al,1993; Jain L et al, 2006; Rosner et al, 2002). As pointed out by Rosner the RDT design is a feasible phase II study design for evaluating possible activity of cytostatic anticancer agents whereas historically anticancer drug efficacy was evaluated as being cytotoxic (Rosner et al, 2002).

As laid down in section 3.2 sorafenib is an oral multi-kinase inhibitor that targets the Raf/MEK/ERK signaling pathway in the cell and receptor tyrosine kinases (RTKs) such as VEGFR-2, VEGFR-3, and PDGFR-ß involved in tumor cell proliferation and angiogenesis. The primary clinical benefit of sorafenib is expected to be disease stabilization rather than tumor shrinkage. As disease stabilization is substantially influenced by the natural disease of a

disease, the RDT design was chosen in several phase II trials evaluating possible activity of sorafenib (such as Ratain et al, 2006; Eisen et al, 2006; Pacey et al, 2009)

Taking into account that uveal melanoma is a rare disease with presumably unidentified prognostic factors the RDT design seems to be useful for objective evaluation of time to progression and overall survival.

The RDT design will ensure that all patients receive treatment with sorafenib for a run-in phase of 8 weeks and will receive further treatment with sorafenib if they experience response (complete response or partial response). Patients who experience tumor progression during this run-in phase will not remain in the study but may be offered alternative treatment. Patients who experience tumor stabilization (stable disease) will be randomized double-blinded to either sorafenib or placebo. Tumor response assessments will be performed every 8 weeks, in case of progression the patient will be unblinded and offered retreatment with sorafenib if he had been randomized to placebo. Thus, if the patient experiences progression, the effective maximum duration of a possible placebo application is confined to eight weeks.

3.5 Risk-benefit assessment of the treatment of patients with metastatic uveal melanoma with sorafenib

Based on the rationale for treatment of patients with metastatic uveal melanoma with sorafenib and own clinical experience in a limited number of patients with metastatic uveal melanoma treated with sorafenib on a compassionate use basis (section 3.3), a potential benefit of this systemic treatment is anticipated.

Major potential side effects of the continuous oral treatment with sorafenib are hand-foot-syndrome, diarrhoea and increase in blood pressure which can effectively be reduced either by dose reduction or cessation of treatment with sorafenib in case of hand-foot-syndrome or diarrhoea or antihypertonic agents in case of increase of blood pressure.

Further on, the administration of sorafenib (oral intake) means fewer hospitalizations and thus constitutes an improvement of quality of life.

Thus, treatment with sorafenib is a potentially effective treatment of patients with metastatic uveal melanoma without serious side effects.

In case of short-term evaluation of inappropriate antitumor efficacy patients have the option for alternative treatment strategies either by intra-arterial liver perfusion with fotemustine or melphalan in case of metastatic disease confined to the liver or salvage chemotherapy.

However it should be noted that these treatments do not represent established and authorized alternative treatment options. The only randomized phase III trial comparing intra-arterial hepatic fotemustine administration with intravenous systemic fotemustine with regard to overall survival as primary endpoint is still ongoing (EORTC 18021), all other experience is based on small phase II trials or individual treatments decisions as described in section 3.1. Any locoregional treatment such as surgery or intra-arterial hepatic infusion with fotemustine or mephalan requires that the patient does not suffer from disseminated metastasis of the liver or extrahepatic localisation of metastases.

Any placement of an intra-arterial catheter and to a lower extent, intravenous catheter is associated with the risks of catheter thrombosis, dislocation, catheter stenosis/obstruction or leakage.

The main serious side effects of melphalan and fotemustine are myelotoxicity with anemia, leucopenia and thrombocytopenia and the risk of developing acute leucemia, gastrointestinal toxicity with nausea, vomiting and diarrhoea, allergic reactions, alopecia, interstitial pneumonia, liver function disorders and renal function disorders.

Considering the poor prognosis of metastatic uveal melanoma and taking into account the lack of an established treatment to treat metastatic uveal melanoma, the risk-benefit relation of the study is assessed as positive. The possible benefit of achieving disease control with sorafenib outweighs the risk of possible side effects of sorafenib. Alternative treatment options are likewise further subject to investigation, have probably more serious side effects and certainly affect the quality of life far more as result of the intraarterial/intravenous infusional application in contrast to oral intake of sorafenib.

4.0 Study objectives and endpoints

4.1 Study objectives

4.1.1 Primary objective

- To determine progression free survival (PFS) of sorafenib versus placebo after random assignment (randomized subset only).

4.1.2 Secondary objectives

- To compare safety and tolerability in randomization phase (randomized subset only)

The following secondary objectives refer to all patients enrolled:

- To determine median overall survival

- To determine disease control rate (DCR)
- To determine overall PFS and time to progression (TTP)
- To determine response rate
- To determine whether tumor markers correlate with clinical benefit and whether tumor markers in run-in phase predict clinical benefit
- To determine PFS and TTP after unblinding and retreatment with sorafenib (only in subjects randomized to placebo and retreated with sorafenib)
- Safety and tolerability

4.2 Study endpoints

4.2.1 Primary endpoint

- PFS under treatment with sorafenib versus treatment with placebo after random assignment to blinded study medication in the randomized subset (tumor assessment according to RECIST version 1.1 criteria)

4.2.2 Secondary endpoints

Safety variables:

- Type, incidence and severity of adverse events in the verum and the placebo group in the randomization phase (after random assignment to blinded study medication in the randomized subset until discontinuation of blinded study medication)
- Type, incidence and severity of adverse events in all patients

Efficacy variables:

- Overall PFS (tumor assessment according to RECIST version 1.1 criteria)
- Overall survival
- Overall response rate and DCR (tumor assessment according to RECIST version 1.1 criteria)
- PFS and TTP after unblinding and retreatment with sorafenib (only in subjects randomized to placebo and retreated with sorafenib; referring to the last tumor assessment before unblinding as “baseline” value)

5.0 Experimental plan

5.1 Overall study design

This is a prospective, multicenter, randomized discontinuation phase II trial (open label in the run-in phase and randomized, double-blind, placebo-controlled in the randomization phase).

Patients suffering from histologically or cytologically confirmed metastatic uveal melanoma will receive open label treatment with 400 mg sorafenib during a run-in phase of 8 weeks.

After the run-in phase tumor response will be determined according to RECIST version 1.1 (Eisenhauer, 2009). Patients with complete response (CR) or partial response (PR) will continue to receive open label sorafenib (400 mg bid or reduced dose level in case of toxicity if required according to the dose modifications schedule in Section 9.0) until disease progression or occurrence of unacceptable toxicity.

Patients with progression (PD) will have to discontinue study treatment.

Patients with stable disease (SD) will be randomized to receive double-blind treatment with either 400 mg bid sorafenib or placebo to sorafenib until progression according to RECIST version 1.1 or unacceptable toxicity in a 1:1 ratio. However, patients, who received a reduced dose level of sorafenib in the run-in phase because of any toxicity as required according to the dose modifications schedule in Section 9.0, will continue to receive this dose level after randomization to sorafenib or placebo (i.e instead of 400 mg bid sorafenib or 400 mg placebo the respective dose level will apply for the dose of sorafenib ***and*** placebo).

However, for patients who experience a progression at any time during the randomization phase, the blind should be broken. Patients who have received placebo during the randomization phase should be offered continuing treatment with sorafenib 400 mg bid orally at the investigator’s discretion. Patients randomized to sorafenib should be taken off study medication. However, continuing treatment with sorafenib may be offered according to the investigator’s discretion.

The patients are planned to be seen after 2 weeks and 8 weeks during the run-in phase and every 8 weeks during the randomization phase/continued open treatment phase for safety assessments. Patients will be contacted additionally via telephone by the investigator after 4 weeks in the run-in phase and in every interval between the regular study visits in the randomization phase/continued treatment phase (after 4 weeks) to gain information about the general health condition and possible adverse events. Tumor response will be evaluated after the run-in phase and every eight weeks during the randomization phase/continued treatment

phase with sorafenib by means of magnetic resonance imaging (MRI) in combination with an additional diffusion weighted imaging (DWI).

Sorafenib and placebo to sorafenib will be taken continuously; however, 4 weeks (28 days) will be counted as one cycle for practical reasons. Maximal treatment duration with sorafenib/placebo to sorafenib will be one year.

After discontinuation of study medication all patients will be followed up in 8-week intervals for at least one year to obtain information about progression and survival status. If progression has been documented earlier, only information about survival status will be requested.

The study design is displayed in the following diagram:

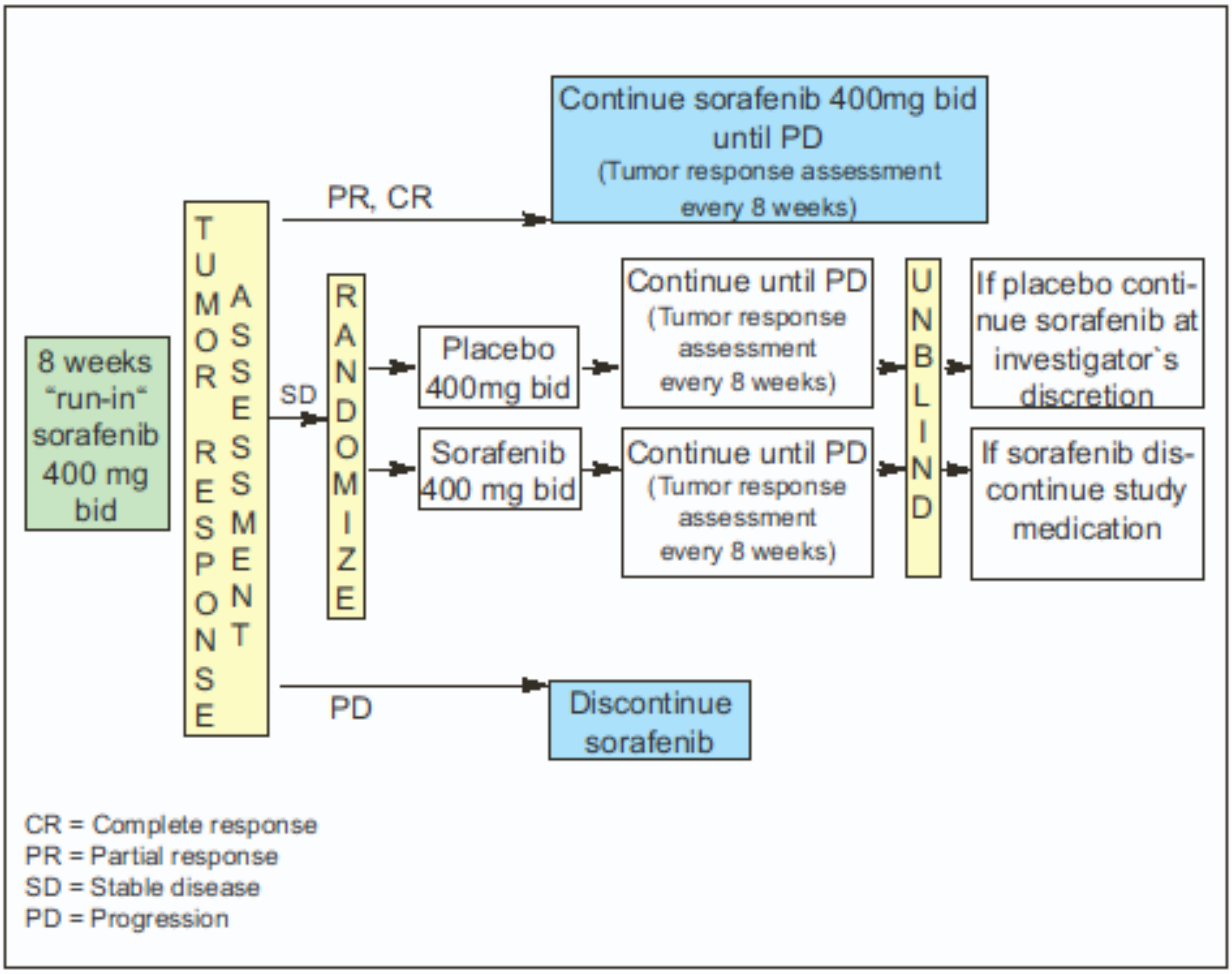

If superiority of sorafenib can be shown due to the results of the interim analysis, all patients still receiving blinded study medication will be unblinded as soon as possible on a cut-off date. Irrespective of their treatment allocation all patients are allowed to receive further open-

label treatment with sorafenib until occurrence of progression. Patients who are receiving open-label sorafenib will receive further treatment with open-label sorafenib until progression. This will also apply to patients in the run-in phase receiving open-label sorafenib who will not be randomized any more. No further patients will be recruited or screened for the study.

5.2 Time schedule

Accrual period:	The accrual period is estimated to last two years.
Study duration:	Study duration is estimated to be 5 years from the first patient enrolled until the end of the study (estimated 3 years duration of recruitment, estimated maximal 1 year treatment with study medication, follow-up in each patient until death or for at least one year after last study drug application). Treatment duration in the individual patient will differ; estimated treatment duration will be several months up to one year.
Begin of the Study:	First patient First visit (FPFV): Date of the written informed consent by the first patient enrolled.
End of the study:	Last Visit Last Patient (LPLV) will be the last follow-up visit of the last patient having received study drug.

5.3 Number of patients and centers

Approximately 200 eligible patients with histologically or cytologically proven metastatic uveal melanoma will enter the 2 months run-in-phase to randomize 78 stable patients. About 10% of all eligible patients will be expected to have CR/PR and continue sorafenib in the non-randomized part of this trial. Approximately 50% of all patients who enter the 2 months run-in-phase will be expected to be progressive and stop sorafenib treatment.

The planned number of randomized patients will be 35 per randomization arm. With an estimated drop-out rate of about 10% the number to be included will be 39 patients per randomization arm (78 patients in total).

Enrollment will be continued until a minimum of 78 patients will have been randomized after the run-in phase.

The estimated number of participating study sites is 3, with all study sites being located in Germany.

5.4 Patient screening and eligibility

5.4.1 Patient recruitment

Recruitment of patients will be performed by the investigator at the local study site. Only after being informed about the study by the investigator and after having signed the patient informed consent form, patients will be screened for eligibility. Screening procedures have to be performed within 7 days prior to first application of study treatment unless otherwise specified. Procedures that are part of routine care are not considered as study-specific procedures, but may be used to determine eligibility. However data from any procedure that is considered as routine care and was performed before obtaining the subject's informed consent should be collected only after the subject's informed consent. Patients will be registered after verification of the eligibility criteria.

Every subject will receive a patient identification code.

Registration will be performed by the CRO (ClinAssess) via Fax:

ClinAssess GmbH
Birkenbergstraße 82
51379 Leverkusen
Phone +49-2171-36336-0
Fax +49-2171-36336-55

5.4.2 Patient randomization

Patients with SD at the tumor response assessment at the end of the run-in phase, i.e. at the study visit Day 56 ± 2 days will be randomized in a 1:1 ratio to the verum (sorafenib) and the matching placebo according to the randomization plan (comprising the "*Randomized population*").

Randomization to the respective treatment arm will be performed by the CRO (ClinAssess) via Fax:

ClinAssess GmbH
Birkenbergstraße 82
51379 Leverkusen
Phone +49-2171-36336-0
Fax +49-2171-36336-55

The patient will be allocated a randomization number. The randomization number is imprinted on the label of the medication boxes. Additionally to the randomization number the patient identification number has to be filled into the space provided for the patient identification number on the label of the study medication by the investigator or his/her assignee before dispensing study medication to the patient.

Envelopes with information about the individual patient's allocation to treatment with verum or placebo will be deposited in a safe, locked place at the respective trial sites. In cases of an emergency the envelopes can be opened to unblind the patient's treatment allocation. If the envelope is opened, date, name and reason for opening must be documented on the envelope and in the case report form (CRF).

5.4.3 Inclusion criteria

Patients must meet all of the following inclusion criteria and none of the exclusion criteria (Section 5.4.4) to be eligible:

1. Signed and dated written informed consent before the start of specific protocol procedures

2. Metastatic uveal melanoma with histological or cytological confirmation of liver metastasis (histological or cytological confirmation in case of only extrahepatic metastasis not required for inclusion)
3. By means of whole body MRI documented disease according to RECIST version 1.1 with at least one unidimensional measurable lesion ≥ 10 mm
4. Eastern Cooperative Oncology Group (ECOG) performance status of 0, 1, or 2
5. Male or female patients ≥ 18 years of age
6. Estimated life-expectancy more than 5 months
7. Hematologic function, as follows:
   - Absolute neutrophil count (ANC) ≥ 1.5 x 10^9/L
   - Platelet count ≥ 100 x 10^9/L
   - Hemoglobin ≥ 9 g/dL
8. Renal function, as follows
   - Creatinine ≤ 1.5 x upper limit of normal (ULN)
9. Hepatic function, as follows
   - Aspartate aminotransferase (AST) ≤ 2.5 x ULN (if liver metastases ≤ 5 x ULN)
   - Alanine aminotransferase (ALT) ≤ 2.5 x ULN (if liver metastases ≤ 5 x ULN)
   - Total bilirubin ≤ 3 mg/dl
   - Alkaline phosphatase ≤ 4.0 x ULN
10. PT-INR/PT < 1.5 x ULN
11. Females of childbearing potential (FCBP) must have a negative pregnancy test within 7 days of the first application of study treatment
    and
    must agree to use effective contraceptive birth control measures (combined oral contraceptives, hormone-releasing intrauterine contraceptive device, hormonal contraceptive implants, hormonal contraceptive injectables) in combination with barrier birth control measures during the course of the trial

or be surgically sterile

A female subject is considered to be of childbearing potential unless she is age ≥ 50 years and naturally amenorrhoeic for ≥ 2 year or unless she is surgically sterile.

12. Males must agree to use barrier birth control measures (condomes) during the course of the trial. In addition males must agree to continue to use these barrier birth control measures for at least 3 months after last administration of study medication.

5.4.4 Exclusion criteria

1. Previous or concurrent tumor other than uveal melanoma with the exception of cervical cancer in situ, adequately treated basal cell carcinoma, superficial bladder tumors (Ta, Tis, and T1) or any curatively treated tumors > 3 years prior to enrollment
2. History of cardiac disease: congestive heart failure ≥ NYHA class 2; active coronary artery disease ([CAD], myocardial infarction more than 6 months prior to study entry is allowed), cardiac arrhythmias requiring antiarrhythmic therapy (only beta blockers or digoxin are permitted)
3. QT/QTc-interval prolongation (QTc> 450 msec) on ECG, known Long QT syndrome or known Long QT syndrome in relatives
4. Known HIV infection
5. Known chronic infection with hepatitis B or C
6. Hypokalemia, hypocalcemia, hypomagnesemia or patients under actual treatment against hypokalemia, hypocalcemia, hypomagnesemia
7. Active infection requiring systemic antibiotic/antiviral/antifungal treatment or any uncontrolled infection > Grade 2 NCI-CTCAE
8. Symptomatic brain or meningeal tumors (unless patient is > 6 months from definitive therapy, had a negative imaging study within 4 weeks of study entry and is clinically stable with respect to the tumor at the time of study enrollment)
9. Patients with seizure disorder requiring medication (such as steroids or antiepileptics)
10. History of organ allograft
11. Patients with evidence or history of bleeding diathesis
12. Thrombotic or embolic events within the last 6 months
13. Serious non-healing wound, ulcer or fracture

14. Uncontrolled arterial hypertension with systolic blood pressure >150 mm Hg and/ or diastolic blood pressure > 90 mg Hg despite optimal treatment, determined twice within one week
15. Pregnant or breast-feeding patients
16. Marked claustrophobia
17. Cardiac pacemaker, cochlea implants or other implanted metal devices, residual metal splinters
18. Known allergy to the used study drug sorafenib or to any of its excipients
19. Known hypersensitivity to gadolinium based contrast agents
20. Subject unwilling or unable to comply with study requirements
21. Substance abuse, medical, psychological or social conditions that may interfere with the patient's participation in the study or evaluation of the study results
22. Participation in any clinical study or treatment with an experimental drug or experimental therapy within 28 days prior to study enrollment or during study participation
23. Patients receiving anticoagulation therapy with warfarin or phenprocoumon
24. Treatment with any of the following therapies or drugs:
    - Any prior palliative chemotherapy, tyrosine kinase inhibitors (TKI´s) or antiangiogenics (prior **adjuvant** treatment with vaccine or immunotherapy is allowed provided there is documentation of disease progression)
    - Any chemotherapy, hormonal therapy, immunotherapy, targeted therapy or experimental or approved proteins/antibodies within four weeks prior to study enrollment or during study participation
    - Radiotherapy or brachytherapy within four weeks prior to study enrollment or during study participation except to eye or bone
    - Hepatic chemoembolization within four weeks prior to study enrollment or during study participation:
    - Major surgery within 4 weeks of study enrollment
    - Autologous bone marrow transplant or stem cell rescue within 4 months of study enrollment

- Use of biologic response modifiers, such as G-CSF, within 3 week of study enrollment. (G-CSF and other hematopoietic growth factors may be used in the management of acute toxicity such as febrile neutropenia when clinically indicated or at the discretion of the investigator; however they may not be substituted for a required dose reduction.)
- Patients receiving newly initiated treatment with erythropoietin or dose adjusted treatment with erythropoietin within 8 weeks of study enrollment or during study participation (Patients receiving permanent erythropoietin treatment are permitted provided no dose adjustment was undertaken within 2 months prior to the study or during the study).
- Intake of any drug that could cause QT-interval prolongation during the four weeks prior to study enrollment and during study treatment
- Any St. John's wort containing remedy
- Prior exposure to study drug
- Any prior therapy with high cumulative dose of anthracyclines

5.5 Study procedures

Refer to the schedule of assessment (section 2) for an outline of procedures required.

5.5.1 Screening procedures

The following screening procedures have to be completed within 7 days before the start of double-blinded skin treatment therapy, unless otherwise specified:

- General medical history including demography, pre-existing conditions and previous medication
- Record disease-related medical history and prior treatment of uveal melanoma
- Vital signs, height and weight; blood pressure has to be recorded at least three times at different times of the day on one single day
- Physical examination
- ECOG performance status
- Hematology: complete blood count ([CBC] including hematocrit, hemoglobin, reticulocytes, leucocytes, neutrophils, platelets)
- Coagulation: PTT, PT-INR

- Serum chemistry: blood glucose, electrolytes (sodium, potassium, chloride, calcium, magnesium), phosphate, creatinine, blood urea, uric acid, ALT/GOT, AST/GPT, gamma-GT, total bilirubin, AP, LDH, CRP, total protein, albumin, amylase, and lipase
- Electrocardiogram (ECG)
- Tumor marker: S100, melanoma inhibitory activity (MIA)
- Informed consent for ***optional translational research investigations in tumor tissue*** specimen (only if tumor tissue of primary tumor or tumor metastasis is available).
  Planned investigations: p-ERK, p-p70S6k (immunohistochemical staining); gene expression of VEGF respeptor2, proteoglycans: decorin and versican (immunohistochemical staining), tumor fingerprinting by multi-epitope ligand cartography (MELC)
- ***Optional translational research:*** tumor marker: sVEGF-R2
- ***Optional translational research:*** GNAQ-mutations in serum
- ***Optional translational research***: proteomics (matrix-assisted laser desorption/ionization mass spectrometry [MALDI-MS]
- ***Optional translational research:*** Circulating tumor cells-identification of biomarker
- ***Optional translational research***: extracellular signal-regulated kinase (ERK) - phosphorylation in blood lymphocytes (blood collection between 11.00 am and 1.00 pm on day 14, exact time has to be recorded).
- Pregnancy testing (only FCBP)
- Urinalysis by dipstick (pH, glucose, protein, erythrocytes, leucocytes, nitrite, casts)
- Whole body MRI in combination with whole body diffusion weighted imaging (DWI). (*If DWI cannot be performed in any trial center for logistical obstacles whole body MRI without DWI)*.
- Tumor assessment using RECIST criteria

- Dynamic contrast enhanced liver ultrasound ([DCE-US]; only if DCE-US will be performed at trial center and only if the patient had hepatic metastases in the whole body MRI examination at screening).
- Review of in- and exclusion criteria
- Issue of sorafenib as study medication (only after review of in- and exclusion criteria and patient registration)

Procedures that are part of routine care are not considered as study-specific procedures, but may be used to determine eligibility. The data from any procedures that are considered as routine care and were performed before obtaining the subject's informed consent should be collected only after the subject's informed consent.

5.5.2 Study visit Day 14 ± 2 days

- Vital signs and weight; blood pressure has to be recorded at least three times at different times of the day, review of results of patient's blood pressure self-measurements (patients have to perform self-measurements of blood pressure at least three times daily during the run-in phase and record the result in a diary)
- Physical examination
- ECOG performance status
- Hematology: CBC including hematocrit, hemoglobin, reticulocytes, leucocytes, neutrophils, platelets
- Coagulation: PTT, PT-INR
- Serum chemistry: blood glucose, electrolytes (sodium, potassium, chloride, calcium, magnesium), phosphate, creatinine, blood urea, uric acid, ALT/GOT, AST/GPT, gamma-GT, total bilirubin, AP, LDH, CRP, total protein, albumin, amylase, and lipase
- Tumor marker: S100, MIA
- ***Optional translational research:*** tumor marker: sVEGF-R2
- ***Optional translational research:*** GNAQ-mutations in serum
- ***Optional translational research***: proteomics (MALDI-MS)
- ***Optional translational research:*** Circulating tumor cells-identification of biomarker

- ***Optional translational research***: sorafenib plasma concentration and ERK - phosphorylation in blood lymphocytes (blood collection between 11.00 am and 1.00 pm on day 14, exact time has to be recorded).
- ECG
- MRI liver with additional DWI sequence (only in patients with measurable liver metastases at the screening MRI-DWI evaluation and in trial centers where DWI is performed)
- DCE-US (only if DCE-US will be performed at trial center and only if the patient had hepatic metastases in the whole body MRI examination at screening.)
- Record adverse events
- Record concomitant medication

5.5.3 Telephone contact Day 28 ± 2 days

The investigator will phone the patient to inquire the patient's health conditions and possible adverse events. The date of telephone contact as well as possible AEs have to be recorded in the CRF.

5.5.4 Study visit after run-in phase

The study visit is scheduled for Day 56 ± 2 days (after 8 weeks)

- Vital signs and weight; blood pressure has to be recorded at least three times at different times of the day, review of results of patient's blood pressure self-measurements (patients have to perform self-measurements of blood pressure at least three times daily during the run-in phase and record the result in a diary)
- Physical examination
- ECOG performance status
- Hematology: CBC including hematocrit, hemoglobin, reticulocytes, leucocytes, neutrophils, platelets
- Coagulation: PTT, PT-INR
- Serum chemistry: blood glucose, electrolytes (sodium, potassium, chloride, calcium, magnesium), phosphate, creatinine, blood urea, uric acid, ALT/GOT, AST/GPT, gamma- GT, total bilirubin, AP, LDH, CRP, total protein, albumin, amylase, and lipase

- ECG
- Tumor marker: S100, MIA
- ***Optional translational research:*** tumor marker: sVEGF-R2
- ***Optional translational research:*** GNAQ-mutations in serum
- ***Optional translational research***: proteomics (MALDI-MS)
- ***Optional translational research:*** polymorphism and gene expression in circulating tumor cells
- ***Optional translational research***: sorafenib plasma concentration and extracellular signal-regulated kinase (ERK) -phosphorylation in blood lymphocytes (blood collection between 11.00 am and 1.00 pm on Day 56, exact time has to be recorded).
- Urinalysis by dipstick (pH, glucose, protein, erythrocytes, leucocytes, nitrite, casts)
- MRI in combination with DWI *(If DWI cannot be performed in any trial center for logistical obstacles, MRI without DWI.)*.

  as ***whole body MRI including whole body DWI*** in ***patients with extrahepatic metastasis*** oder if clinically indicated (e.g suspicion of new extrahepatic metastasis)

  Exception: patients with exclusively extrahepatic metastasis at screening MRI will undergo further whole body MRI **without** DWI.

  or

  as ***MRI of the liver including DWI of the liver*** in ***patients with exclusively hepatic metastasis*** at the screening evaluation and no suspicion of extrahepatic metastasis.
- Tumor assessment using RECIST criteria
- DCE-US of liver (only if DCE-US will be performed at trial center and only if the patient had hepatic metastases in the whole body MRI examination at screening. DCE-US will not be utilized for evaluation of response.)
- Record concomitant medication
- Record adverse events

According to the results of the tumor assessment of this visit, patients with CR or PR will continue to receive open label sorafenib treatment.

Patients with PD will have to discontinue sorafenib and undergo the end of study treatment visit.

Patients with SD will be randomized to blinded study medication (sorafenib or placebo to sorafenib).

Randomization will be performed by ClinAssess (Section 5.4.2).

Patients will be dispensed with the respective study medication.

5.5.5 Study visits during continued open label treatment with sorafenib and during randomization phase

Study visits are scheduled to be performed every 8 weeks and whenever clinically indicated. In case of suspected progression an additional study visit with tumor assessment and determination of tumor markers has to be performed before unblinding.

Additionally patients will be contacted via telephone by the investigator in the interval between the regular study visits (4 weeks ± 2 days after the last regular study visit) to gain information about the patient's general health condition and possible adverse events, starting with Day 84 ± 2 days. The date of the respective telephone contacts as well as possible AEs have to be recorded in the CRF.

- Vital signs and weight
- Physical examination
- ECOG performance status
- Hematology: CBC including hematocrit, hemoglobin, reticulocytes, leucocytes, neutrophils, platelets
- Coagulation: PTT, PT-INR
- Serum chemistry: blood glucose, electrolytes (sodium, potassium, chloride, calcium, magnesium), phosphate, creatinine, blood urea, uric acid, ALT/GOT, AST/GPT, gamma- GT, total bilirubin, AP, LDH, CRP, total protein, albumin, amylase, and lipase
- ECG
- Tumor marker: S100, MIA

- Urinalysis by dipstick (pH, glucose, protein, erythrocytes, leucocytes, nitrite, casts)
- MRI in combination with DWI *(if DWI cannot be performed in any trial center for logistical obstacles, MRI without DWI.)*
  as ***whole body MRI including whole body DWI*** in ***patients with extrahepatic metastasis*** oder if clinically indicated (e.g suspicion of new extrahepatic metastasis)
  Exception: patients with exclusively extrahepatic metastasis at screening MRI will undergo further whole body MRI **without** DWI.
  or
  as ***MRI of the liver including DWI of the liver*** in ***patients with exclusively hepatic metastasis*** at the screening evaluation and no suspicion of extrahepatic metastasis
- Tumor assessment using RECIST criteria
- DCE-US of liver (only if DCE-US will be performed at trial center and only if the patient had hepatic metastases in the whole body MRI examination at screening. DCE-US will not be utilized for evaluation of response.)
- Record concomitant medication
- Record adverse events
- Dispense of study medication (if applicable)

5.5.6 End of study treatment visit

The end of study treatment visit has to be performed within 14 days after the last application of study medication (either open-label sorafenib or blinded study medication).

- Vital signs and weight
- Physical examination
- ECOG performance status
- Hematology: CBC including hematocrit, hemoglobin, reticulocytes, leucocytes, neutrophils, platelets
- Coagulation: PTT, PT-INR

- Serum chemistry: blood glucose, electrolytes (sodium, potassium, chloride, calcium, magnesium), phosphate, creatinine, blood urea, uric acid, ALT/GOT, AST/GPT, gamma- GT, total bilirubin, AP, LDH, CRP, total protein, albumin, amylase, and lipase
- ECG
- Tumor marker: S100, MIA
- Pregnancy testing (only FCBP)
- Urinalysis by dipstick (pH, glucose, protein, erythrocytes, leucocytes, nitrite, casts)
- MRI in combination with DWI *(if DWI cannot be performed in any trial center for logistical obstacles, MRI without DWI.)*
  as ***whole body MRI including whole body DWI*** in ***patients with extrahepatic metastasis*** oder if clinically indicated (e.g suspicion of new extrahepatic metastasis)
  Exception: patients with exclusively extrahepatic metastasis at screening MRI will undergo further whole body MRI **without** DWI.
  or
  MRI of the liver including DWI of the liver in ***patients with exclusively hepatic metastasis*** at the screening evaluation and no suspicion of extrahepatic metastasis
- Tumor assessment using RECIST criteria
- DCE-US of liver (only if DCE-US will be performed at trial center and only if the patient had hepatic metastases in the whole body MRI examination at screening. DCE-US will not be utilized for evaluation of response.)
- Record concomitant medication
- Record adverse events
- Return of unused study medication

However, if study treatment was permanently discontinued because of progression under treatment with sorafenib (as result of unblinding) and assessments of tumor markers as well as MRI including DWI, and contrast enhanced liver ultrasound were performed within 14 days

before the end of study treatment visit, these procedures need not to be repeated and only the other listed procedures will be performed.

5.5.7 Follow-up

Every subject will be followed up for anti-tumor therapy and survival status until death or for at least one year after last study drug application, whichever is sooner. The respective information may be requested from the treating physician via telephone, if the patient gives his consent to this procedure.

5.6 Treatment plan

5.6.1 Run-in phase (8 weeks)

During the run-in phase patients will be administered open label 400 mg sorafenib bid (2 x 200 mg tablets twice daily) orally on a continuous basis for 8 weeks.

Sorafenib will be supplied by the sponsor as 200 mg tablets. The tablets should be swallowed with a glass of water. It is recommended that sorafenib should be administered without food or with a low or moderate fat meal. If the patient intends to have a high-fat meal, sorafenib tablets should be taken at least 1 hour before or 2 hours after the meal.

In case of toxicities that are assessed as at least possibly related to sorafenib, the schedules for dose delay and dose modification as specified in section 9.0 will apply.

5.6.2 Continued open-label treatment

Patients who obtained CR or PR at the tumor response assessment after the run-in phase (Day 56 ± 2 days) will continue to be administered open label sorafenib (400 mg bid [2 x 200 mg tablets twice daily] or reduced dose level in case of toxicity if required according to the dose modifications schedule in Section 9.0) orally on a continuous basis.

In case of toxicities that are assessed as at least possibly related to sorafenib, the schedules for dose delay and dose modification as specified in section 9.0 will apply.

Patients are to continue sorafenib until occurrence of PD, occurrence of unacceptable toxicity, or until any other in section 11 listed criterion for discontinuation of study medication applies.

Sorafenib will be supplied by the sponsor as 200 mg tablets. The tablets should be swallowed with a glass of water. It is recommended that sorafenib should be administered without food or with a low or moderate fat meal. If the patient intends to have a high-fat meal, sorafenib tablets should be taken at least 1 hour before or 2 hours after the meal.

5.6.3 Randomization phase

Patients with SD at the tumor response assessment after the run-in phase (Day 56 ± 2 days) will be randomized to receive blinded study medication (either sorafenib or matching placebo) 400 mg bid (2 x 200 mg tablets twice daily) orally on a continuous basis. However, patients, who received a reduced dose level of sorafenib in the run-in phase because of any toxicity as required according to the dose modifications schedule in Section 9.0, will continue to receive this dose level after randomization to sorafenib or placebo (i.e instead of 400 mg bid sorafenib or 400 mg placebo the respective dose level will apply for the dose of sorafenib ***and*** placebo).

In case of toxicities that are assessed as at least possibly related to study medication, the schedules for dose delay and dose modification as specified in section 9.0 will apply.

Patients are to continue study medication until occurrence of PD, occurrence of unacceptable toxicity, or until any other in section 11 listed criterion for discontinuation of study medication applies.

Sorafenib and matching placebo will be supplied by the sponsor as 200 mg tablets. The tablets should be swallowed with a glass of water. It is recommended that the study medication should be administered without food or with a low or moderate fat meal. If the patient intends to have a high-fat meal, study medication tablets should be taken at least 1 hour before or 2 hours after the meal.

For patients who experience progression (either clinical progression or progression according to RECIST criteria) at any time during the randomization phase, the blind should be broken. Patients having received placebo should be offered continuing treatment with sorafenib 400 mg sorafenib bid (2 x 200 mg tablets twice daily) orally on a continuous basis at the investigator's discretion.

Patients randomized to sorafenib should be taken off study medication. However, continuing treatment with sorafenib may be offered according to the investigator's discretion.

The request to unblind the respective patient's treatment allocation in case of progression during the randomization phase will have to be sent to the CRO and will be answered by the CRO via Fax:

ClinAssess GmbH
Birkenbergstraße 82
51379 Leverkusen
Phone +49-2171-36336-0
Fax +49-2171-36336-55

6.0 Efficacy assessments

6.1 Magnetic resonance imaging

Tumor response will be assessed with MRI using RECIST-criteria (RECIST guidelines version 1.1 [Eisenhauer, 2009]).

MRI will be performed in combination with DWI, however for tumor assessment according to RECIST criteria only the routine MRI evaluation will be utilized. **If DWI cannot be performed in any trial center for logistical obstacles, MRI examinations without DWI will be acceptable.**

DWI is a functional technique in MRI that displays information about the extent of free water motion in tissues. Preclinical and clinical data indicate a number of potential roles of DWI in malignant tumors including monitoring response to therapy (Patterson, 2008). Changes in diffusion often precede changes in lesion size. Moreover, DWI seems to be highly sensitive for liver metastases of melanoma and therefore important for defining the tumor extent at baseline (Kalkmann, 2010).

Whole body MRI in combination with whole body DWI will be performed during screening (within 7 days before start of study treatment with sorafenib) according to the specifications in Appendix V.

Patients who exclusively suffer from liver metastases during the screening evaluation and are not suspected to suffer progression due to incidence of extrahepatic metastases during any time of the study course will receive MRI of the liver in combination with DWI of the liver in the identical sequences during the following MRI examinations, patients with evidence or suspicion of extrahepatic metastasis will undergo further whole body MRI in combination with whole body DWI in the identical sequences. Patients with exclusively extrahepatic metastasis at screening will undergo further whole body MRI without DWI. MRI scans have to include evaluations of all suspected sites of the disease and must be obtained with identical modality and technique to those obtained at baseline.

The routine MR examinations of the liver (T1w, T2w axial pro-contrast, multiphase 3D T1w contrast imaging, T1w post contrast) as part of the MRI for morphological lesion and response assessment will be combined with a DWI sequence (axial EPI, b50, b500 and b1000 including ADC calculation).

Further MRI examinations and assessments of tumor response using RECIST criteria (RECIST guidelines version 1.1) have to be performed at the end of the run-in phase after eight weeks of study treatment with sorafenib prior to randomization or continued sorafenib therapy; every eight weeks during continued sorafenib therapy and during the randomization phase (as well as in addition if clinically indicated such as in case of suspected progression between the eight-week intervals); and at the ‘end to treatment visit’.

All patients with hepatic metastases will receive an additional MRI of the liver in combination with DWI of the liver two weeks after start of study medication sorafenib (Day 14) to evaluate the potential of an early response in DWI, if DWI is performed at the respective trial site.

6.2 Dynamic contrast enhanced liver ultrasound

DCE-US of the liver will not be performed in all trial centers due to methodical or financial limitations. DCE-US examinations of the liver will be performed as additional assessment to evaluate angiogenesis and vascularity of liver lesions and monitor response to study treatment. SonoVue® manufactured by Bracco Imaging Germany GmbH, Konstanz will be used as contrast medium. DCE-US of the liver will be performed only if the patient had hepatic metastases in the whole body MRI examination at screening.

In order to obtain comparable results, the DCE-US examinations have to observe the validated rules.

Assessments will be performed at the same time points and intervals as MRI. However, only the MRI evaluation will be utilized for the assessment of tumor response according to RECIST criteria.

6.3 Tumor markers

S100 and MIA will be determined as established tumor markers for malignant melanoma (Barak et al, 2007) at screening (within 7 days before start of study treatment with sorafenib), on Day 14 ± 2 days and Day 56 ± 2 days during the run-in phase, as well as every eight weeks during continued sorafenib therapy and during the randomization phase (additional assessments in case of suspected progression between the eight-week intervals), and within 14 days after the end of treatment.

MIA (melanoma inhibitory activity) protein, identified as a small 11 kDa protein highly expressed and secreted by malignant melanoma cells, plays an important functional role in melanoma development, progression and tumor cell invasion. Recent data describe a direct

interaction of MIA protein with cell adhesion receptors and extracellular matrix molecules (Schmidt J and Bosserhoff AK, 2009). Besides Osteopontin (OPN) and S-100beta, MIA has recently been established as a valuable serum marker to detect metastatic uveal melanoma (Klingenstein A et al, 2010; Haritoglou I et al, 2009; Barak V et al, 2007; Reiniger IW et al, 2005). Most interestingly, responder patients demonstrate a quick and dramatic drop in MIA serum levels upon short (2weeks) Sorafenib treatment, making MIA a promising tool for patient selection.

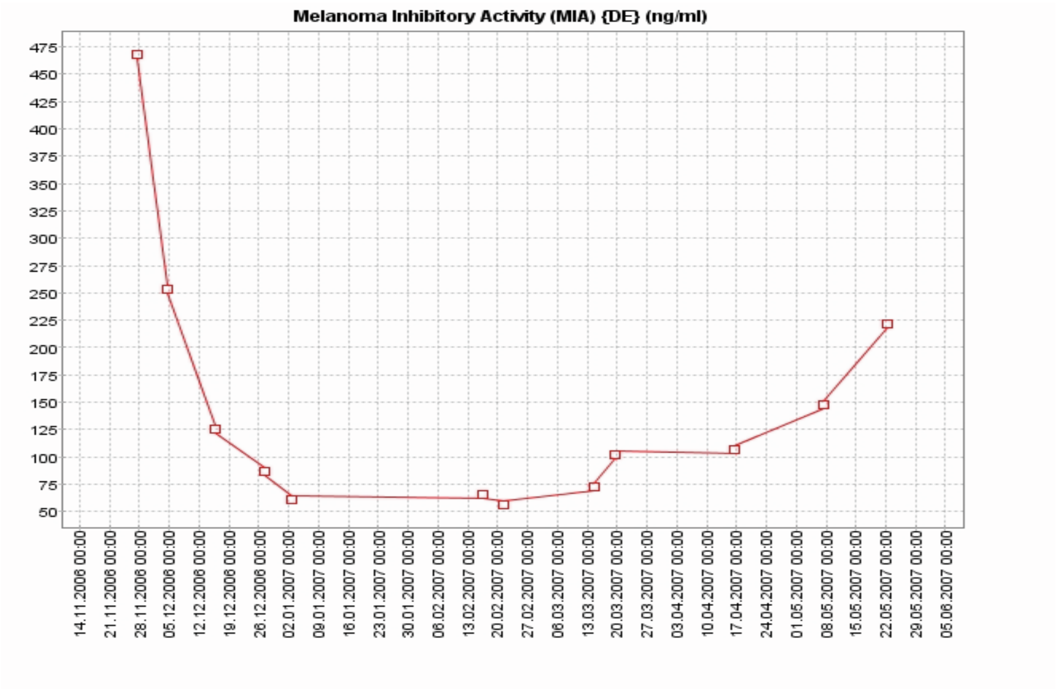

Courtesy of E. Kämpgen, Dept. Dermatology, Erlangen

S100 will be analyzed in the following laboratory, needed are 4 ml blood per visit to obtain serum:

Central laboratory
University Hospital Essen
Hufelandstr. 55
45122 Essen

MIA will be analyzed in the following laboratory, needed are 4 ml blood per visit to obtain serum:

Department of Dermatology
University Hospital Erlangen
Hartmannstr. 14
91052 Erlangen
Head: Uwe Koch, PhD
Responsible scientist: Eckhart Kaempgen, MD

7.0 Translational research

All the translational research analyses are optional. Subjects have to give their explicit informed consent to any translational research procedure. Eligible subjects may participate in the trial without giving consent to translational research procedures.

The translational analyses are described in the following sections.

7.1 Tumor marker VEGF-R2

Blood samples (2.7 ml with EDTA as anticoagulant) for assessment of the tumor marker sVEGF-R2 will be drawn at screening (within 7 days before start of study treatment with sorafenib), and on Day 14 ± 2 days and Day 56 ± 2 days during the run-in phase.

Samples have to be centrifuged for 15 minutes at 1000 x g within 30 minutes of collection and stored at ≤ 20 degree Celsius. Specimens frozen at ≤ 20 degree Celsius have to be shipped for analysis to the following laboratory:

Pharmacology of neoplastic drugs
Department of Medicine (Cancer Research)
of the West German Cancer Center
University Hospital Essen
GBK-Haus, 1 Etage
Hufelandstr. 55
45122 Essen
Responsible scientist: Ralf Axel Hilger, PhD

For determination of sVEGF-R2 the Quantikine® Human Soluble VEGF R2 Immunoassay by R&D Systems will be utilized.

7.2 Pharmacokinetic and ERK-phosphorylation in blood lymphocytes

Sorafenib steady state plasma concentration and ERK -phosphorylation in blood lymphocytes will be determined on Day 14 ± 2 days and Day 56 ± 2 days during the run-in phase. Additonally, ERK-phosphorylation in blood lymphocytes will be determined during screening as baseline value. Because of the circadian rhythm in the regulation of the MAP kinase pathway the blood samples (on both days 2 x 2.7 ml blood with EDTA as anticoagulant) have to be drawn between 11.00 a.m. and 1.00 p.m., the exact date and time of blood collecting must be recorded in the CRF. Pharmacokinetic and pharmacodynamic evaluations will be performed by the following laboratory:

Pharmacology of neoplastic drugs
Department of Medicine (Cancer Research)
of the West German Cancer Center
University Hospital Essen

GBK-Haus, 1 Etage
Hufelandstr. 55
45122 Essen
Responsible scientist: Ralf Axel Hilger, PhD

Further instructions for pharmacokinetic/pharmacodynamic sample collection and handling are summarized in Appendix III.

7.3 Proteomics

Blood samples (2.7 ml blood with EDTA as anticoagulant) for assessment of proteomics as possible tumor marker will be drawn at screening (within 7 days before start of study treatment with sorafenib), and on Day 14 ± 2 days and Day 56 ± 2 days during the run-in phase. Samples have to be centrifuged within 30 minutes of collection and. stored at ≤ 70 degree Celsius. Specimens frozen at ≤ 70 degree Celsius have to be shipped to:

Biodesix Inc.
Heinrich Roder
520 Zhang Street
Suite 213
Broomfield
Colorado 80021
USA
Phone: 001-9708709041
E-mail: hroder@biodesix.com

Proteomics will be measured by MALDI-MS.

7.4 Identification of biomarker in circulating tumor cells

As recently discussed hematogenous metastasis may be an early event in tumor evolution of solid malignancies (Klein CA, 2008; Podsypanina K2008). As for uveal melanoma the development of liver metastasis is a frequent event, hematogenous metastasis seems to be the primary way of tumor spread (Foss AJ et al, 1995, Keilholz U et al, 2004; Schuster R et al, 2007). Up to date studies used tyrosinase and MelanA/MART1 mRNA Expression Analysis to quantify tumor load in peripheral blood and indirectly quantify the increase or decrease of circulating tumor cells (CTC) to estimate the individual risk for development of metastasis and to predict therapeutic response to locoregional or systemic therapy. Non-specific expression analysis from total blood RNA and the potentially small range of identifiable prognostic and predictive markers obtained, seem to be pitfalls of this approach. A new technique was developed to specifically enrich circulating tumor cells and to confirm their existence by immunohistochemistry. After confirmation of tumor cells expression analysis from highly specific cell populations from CTCs was used to then identify and validate new

biomarkers for the assessment of aggressiveness of the tumor and for predicting the response and survival rate of each individual based on the genetic profile.

Sample quality probably contributes to the result of the analysis (Keilholz U et al, 2004; Schuster R et al, 2007). The sensitivity of the methods does not permit any transport, the investigations have to be performed in local laboratories of the respective trial centers

Blood samples will be analyzed in the following laboratories:

Department of Medicine (Cancer Research)
of the West German Cancer Center
University Hospital Essen
Hufelandstr. 55
45122 Essen
Responsible scientist: Claudius Andreas Hoffmann, MD

Charité Tumor Center
Department of Medicine III
Campus Benjamin Franklin
Hindenburgdamm 30
12220 Berlin
Responsible scientist: Ulrich Keilholz, MD, PhD

Department of Dermatology
University Hospital Erlangen
Hartmannstr. 14
91052 Erlangen
Responsible scientist: Eckhart Kaempgen, MD

In the laboratory in Essen circulating tumor cells will be analyzed as follows:

A blood sample (at least 20 ml with sodium citrate as anticoagulant) for investigation of circulating tumor cells will be drawn at screening (within 7 days before start of study treatment with sorafenib), and on Day 14 ± 2 days and Day 56 ± 2 days during the run-in phase.

Mononuclear cells and circulating tumor cells will be enriched from peripheral venous blood using a specifically designed buoyant density gradient centrifugation. Then immuno-magnetic beads with anti-CD15, anti-CD19 and anti-CD45 monoclonal antibodies will be used to negatively select hematopoietic cells (Odashiro DN, 2006). The remaining cell suspension will then be immunohistochemically stained by a trained pathologist after fixation on a glass slide. In this way circulating tumor cells can be identified, microdissected, and RNA respectively DNA can be isolated. Linear amplification techniques will be used to yield higher amount of DNA and RNA for subsequent analysis. Gene/SNP-array and quantitative

real-time polymerase chain reaction (RT-PCR) will be used to examine polymorphisms and gene expression associated to progression-free and overall survival of patients with metastasized uveal melanoma. The aim is to identify markers that are of predictive value for therapeutic response.

Further details to this project can be looked up in Appendix IV.

7.5 Tumor-specific GNAQ-mutations in serum

The aim of this research is to establish a marker for an early phase of metastasis of uveal melanoma. Such a marker would be useful to identify patients with metastasis early in order to offer adequate treatment options. Possible marker will be examined whether they are appropriate to monitor the course of disease under treatment.

Most cutaneous melanomas show an activation of the MAP-kinase pathway via BRAF- and NRAS-mutations (Fecher et al, 2008). Activating mutations in the GNAQ gene that codes the stimulatory alpha G-protein subunit are an alternative way of activation of MAP-kinase . These mutations were found in intradermal melanocytic lesions, eg. nevus of ota and blue nevi. Further on it could be shown that about 50% of uveal melanoma have activating mutations of the GNAQ gene (Onken et al, 2008; Van Raamsdonk, 2009). These mutations are confined to tumor cells and are not found in normal cells of the peripheral blood. Therefore proof of mutated DNA in serum indicates presence of uveal melanoma cells. Blood samples (ca. 4 ml) will be drawn at screening (within 7 days before start of study treatment with sorafenib), on Day 14 ± 2 days and Day 56 ± 2 days during the run-in phase. These blood samples will be used as positive controls in a research project to identify tumor-specific GNAQ mutations by means of DNA sequencing techniques (Genome Sequencer GS Junior Roche). The aim is to identify tumor-specific GNAQ mutations that represent possible biomarker in an early stage of tumor metastasis.

Samples have to be centrifuged within 30 minutes of collection, stored at ≤ 20 degree Celsius and shipped to:

Department of Ophthalmology (Department for posterior segment diseases)
University Hospital Essen
Hufelandstr. 55
45122 Essen
Responsible scientist: Claudia Metz, MD

7.6 Translational research investigations in tumor tissue

If a tumor tissue specimen of the primary tumor or tumor metastases is available before or during study participation, subjects will be asked for consent to perform the following

investigations in the tumor tissue specimen. These investigations will be performed only after all for clinical reasons necessary assessments such as histological confirmation of the tumor diagnosis have been performed in the tissue specimen. Eligible subjects may participate in the trial without giving consent to translational research investigations in the tumor tissue specimen.

The following investigations in the tumor tissue specimen will be performed in the Institute of Pathology, West German Cancer Center, University Hospital Essen, Essen:

- p-ERK and p-p706k to assess the phosphorylation state of these kinases in uveal melanoma tissue (immunohistochemical staining)
- Gene expression of VEGF receptor 2
- Proteoglycans: decorin and versican (immunohistochemical staining)

Sorafenib is a small molecular inhibitor of several tyrosine protein kinases. These pathways include the Raf/Mek/Erk pathway (MAP Kinase pathway) and the VEGF receptor 2 associated pathway.
It could be shown, that the detection of phosphorylated ERK by means of immunohistochemistry is an independent prognostic parameter in human hepatocellular carcinoma (Schmitz KJ et al, 2008). It was also found that phosphorylation of p70S6 kinase predicted overall survival in patients with clear margin-resected hepatocellular carcinoma (Baba HA et al., 2009). This kinase is a key downstream target of mTOR, the mammalian target of rapamycin (mTOR) kinase. Potent inhibitors of mTOR are rapamycin and its derivatives. Besides their immunosuppressive effects, mTOR inhibitors exhibit a potent growth repressive effect against various tumor entities in preclinical and early clinical trials. From these data it is clear that deregulation of particular signaling pathways play an important role in tumor genesis and tumor progression.
The phosphorylation status of p-ERK and p-p706k as well as the gene expression of the VEGF receptor 2 before study treatment will be compared with the course of disease and clinical outcome under treatment with sorafenib to get further information with regard to their importance for treatment with sorafenib in uveal melanoma.
Kim (Kim S et al, 2009) could show that the proteoglycan versican strongly enhanced metastatic growth in Lewis lung carcinoma cell lines by activating toll-like receptors (TLR) 2:TLR6 complexes and inducing TNF-α secretion by myeloid cells.
Tumor tissue specimens for these analyses have to be shipped to:

Institute of Pathology
West German Cancer Center
University Hospital Essen
Hufelandstr. 55
45122 Essen
Responsible scientist: Hideo A. Baba, MD

The following investigations in the tumor tissue specimen will be performed in Toponome Research Facility, Universitätsklinikum Erlangen, Erlangen:

- Tumor fingerprinting by MELC

Further details to this project can be looked up in Appendix VI.

Tumor tissue specimens for these analyses have to be shipped to:

Toponome Research Facility
Universitätsklinikum Erlangen
Hartmannstr. 14
91052 Erlangen
Responsible scientist: Andreas Baur, PhD

8.0 Study drug

8.1 General information

Sorafenib is a multikinase inhibitor which has demonstrated both anti-proliferative and anti-angiogenic properties *in vitro* and *in vivo*.

In vitro sorafenib decreases tumor cell proliferation. Sorafenib inhibits tumor growth of a broad spectrum of human tumor xenografts in athymic mice accompanied by a reduction of tumor angiogenesis. Sorafenib inhibits the activity of targets present in the tumor cell (CRAF, BRAF, V600E BRAF, c-KIT, and FLT-3) and in the tumor vasculature (CRAF, VEGFR-2, VEGFR-3, and PDGFR-β). RAF kinases are serine/threonine kinases, whereas c-KIT, FLT-3, VEGFR-2, VEGFR-3, and PDGFR-β are receptor tyrosine kinases (Nexavar®, SmPC, 2009).

As Nexavar® sorafenib received a marketing authorization in the European Union for the treatment of patients with hepatocellular carcinoma and patients with advanced renal carcinoma who have failed prior interferon-alpha or interleukin-2 based therapy or are considered unsuitable for such therapy in July 2006.

Sorafenib as monotherapy or combination therapy is assessed in a variety of ongoing phase II and phase III trials, among others in the indication breast cancer, thyroid cancer, acute myeloid leukemia, and non-small cell lung cancer.

8.2 Formulation

A 200 mg tablet formulation of sorafenib will be used in this trial. Sorafenib will be processed as tosylate salt of sorafenib.

The chemical name of sorafenib tosylate is 4-(4-{3-[4-Chloro-3-(trifluoromethyl)-phenyl]-ureido}phenoxy)-*N*^{2}-methylpyridine-2-carboxamide-4-methylbenzenesulfonate.

Sorafeib tosylate is a white to yellowish or brownish solid with a molecular formula of $C_{21}H_{16}ClF_3N_4O_3$ x $C_7H_8O_3S$ and a molecular weight of 637.0 g/mole.

Each tablet contains sorafenib tosylate equivalent to 200 mg sorafenib and the following ingredients: croscarmellose sodium, microcrystalline cellulose, hypromellose, sodium lauryl sulfate, magnesium stearate, and the coating consisting of hypromellose, polyethylengylcol, titanium dioxide and ferric oxide red.

Appearance and weight of placebo tablets will be identically to those of sorafenib tablets. However, the placebo formulation will contain only inert tablet ingredients including croscarmellose sodium, microcrystalline cellulose, lactose, magnesium stearate and a coating consisting of hypromellose, polyethylengylcol, titanium dioxide, and ferric oxide red.

8.3 Processing, packaging and labeling

200 mg sorafenib tablets for open label treatment with sorafenib as well as the study medication for the randomization phase (either 200 mg sorafenib or matching placebo) is processed and supplied by Bayer Schering Pharma AG, 13342 Berlin.

Packaging and labeling will be performed by:

University Hospital Dresden
Clinic Pharmacy
Fetscherstr. 74
01307 Dresden
Contact person: Dr. rer. nat. Ina-Maria Klut
Phone: +49 351 458-4625 / -0
Fax: + 49 351 458-6385

Labelling will be in accordance with German GCP-V § 5 and other applicable national regulations in the German Drug Law (AMG) and GCP-V in effect.

The label for open label sorafenib will contain name and contact information (including telephone-no.) of the sponsor, the trial protocol code and EudraCT-No., the agent (i.e.

sorafenib) and dose, batch-no., number of tablets, pharmaceutical form and route of administration, storage conditions, expiry date and instructions about special precautions, medication-no., and a space to enter the patient identification code.

The label for blinded study medication will contain information about name and contact information (including telephone-no.) of the sponsor, the trial protocol code and EudraCT-No., the agent (i.e. sorafenib/placebo to sorafenib) and dose, batch-no., number of tablets, pharmaceutical form and route of administration, storage conditions, expiry date and instructions about special precautions, randomisation-no., and a space to enter the patient identification code.

The list with the randomisation numbers and the information whether a package contains verum or placebo will be stored at a safe, locked place at the CRO as sponsor representative (without access for any personnel involved in the management of the trial) and the Clinic Pharmacy in Dresden.

8.4 Randomization and distribution of study medication

Sorafenib for the run-in phase and continued open label treatment with sorafenib will be distributed to the investigators by Clinic Pharmacy, University Hospital Dresden. The patient identification number corresponding with the patient’s CRF number has to be filled into the space provided for the patient identification number on the label of the study medication by the investigator or his/her assignee before dispensing study medication to the patient.

Blinded study medication during the randomization phase containing either sorafenib or placebo to sorafenib will be distributed to the investigators by Clinic Pharmacy, University Hospital Dresden. Neither the investigator nor the patient will know the treatment allocation until the treatment allocation is unblinded in case of progression.

Randomization will be carried out before start of the trial by compiling a randomization list. Randomization according to the randomization plan will be performed by ClinAssess (see Section 5.4.2) on request. After randomization of a patient according to the randomization plan ClinAssess will inform the investigator about the randomization number, which is imprinted on the label of the medication bottle. The patient identification number has to be filled into the space provided for the patient identification number on the label of the study medication by the investigator or his/her assignee before dispensing study medication to the patient.

For emergency cases the treatment assignment of an individual patient will be documented in a sealed envelope carrying the patient number, which will be sent to the investigator together

with the first study medication shipment of blinded study medication. These envelopes have to be stored in a locked, safe place and may be opened by the treating investigator only in case of an emergency. If the envelope is opened, date and reason for opening must be documented on the envelope. Not opened envelopes must be returned at the end of the trial. The emergency envelopes must not be used for unblinding the patient's treatment allocation in case of progression.

The request to unblind the respective patient's treatment allocation in case of progression will have to be sent to the CRO and will be answered by the CRO via fax (see section 5.6.3).

8.5 Storage of study medication

At the study site, study drugs have to be stored in a locked, safe area under the responsibility of the (hospital) pharmacist, or the investigator, or other personnel allowed storing and dispensing study drug to prevent unauthorized access, according to national regulations.

The study drug should be stored at room temperature equal to or below 25 degree Celsius.

The investigator is responsible to ensure that the investigational drugs are used only in accordance with the protocol and under no circumstances will be supplied to third parties.

If any study drug is lost or damaged, its disposition should be documented in the source documents. Patients will be instructed to return unused tablets to the study site.

The investigator or a pharmacist or other appropriate individual who is assigned by the local principal investigator should maintain records of the inventory at the site, the use for each subject, and the delivery, storage and destruction of study medication or return of the study medication to the sponsor respectively in accordance with the national regulations in effect.

9.0 Dose modifications of study medication

9.1 Dose reduction levels

Doses of study medication (open label sorafenib or blinded study medication during the randomization phase) will be delayed or reduced for clinically significant hematologic and/or other toxicities that are assessed as at least possibly related to study medication. Toxicities will be graded using the NCI CTCAE version 4.0. If a patient experiences several toxicities and there are conflicting recommendations, the recommended dose adjustment that reduces the dose to the lowest level will be used. All dose modifications will be performed in predefined dose levels. The predefined dose levels are as follows:

**Table 1**: **Dose reduction schedule of study medication (sorafenib or placebo to sorafenib)**	
**Dose level**	**Dosage**
Dose level 0:	400 mg (2x200 mg tablets) po bid
Dose level -1	400 mg (2x200 mg tablets) po once daily
Dose level -2	400 mg (2x200 mg tablets) po once every other day

If more than 2 dose reductions are required, the patient should be discontinued from the study. Also, at the discretion of the investigator, the dose may be re-escalated to 400 mg po bid or to 400 mg po once daily after the resolution of the adverse event.

Patients who are treated in the blinded randomization phase should be unblinded by the investigator only in the case of an emergency or in case of progression.

In the case **surgical treatment** is required, treatment with sorafenib/placebo should be discontinued two days prior to the planned surgery and restarted once wound healing is complete, however in case of necessary dose interruption of ≥ 4 weeks study treatment has to be discontinued permanently. If the patient experiences a **bleeding ulcer** during the study, treatment with sorafenib/placebo should be discontinued and restarted once wound healing is complete. However, in case of necessary dose interruption of ≥ 4 weeks, study treatment has to be discontinued permanently.

9.2 Management of treatment-associated skin toxicity

Hand-foot skin reaction (HFSR) also called hand-foot syndrome or palmar-plantar erythrodysesthesia frequently occurred in trials when patients were treated with sorafenib (in 18-19 % of all patients, according to the German SmPC Nexavar®). Subjects experiencing HFSR should have their signs and symptoms graded according to CTCAE version 4.0. Other skin toxicities will also be graded according to CTCAE version 4.0.

**Table 2: Grading for hand-foot skin reaction**	
Grade 1	Minimal skin changes or dermatitis (e.g. erythema, edema, or hyperkeratosis) without pain
Grade 2	Skin changes (e.g. peeling, blisters, bleeding, edema, or hyperkeratosis) with pain; limiting instrumental activities of daily life

Grade 3	Severe skin changes (e.g. peeling, blisters, bleeding, edema, or hyperkeratosis) with pain; limiting basic self-care activities of daily life

Dose modification and dose interruptions of study medication due to hand-foot skin reaction and other skin toxicities are listed in Table 3.

Table 3 illustrates the dose modifications and dose interruptions on occurrence of HFSR and other skin toxicities.

Table 3 Dose modification and delays due to skin toxicities (open-label sorafenib or blinded study medication)

Skin Toxicity Grade	Occurrence	Dose modification/ supportive measures
Grade 1	Any occurrence	Immediate start of supportive skin measures without any dose modification
Grade 2	1st occurrence	Immediate start of supportive measures, decrease study medication 1 dose level for 28 days.<br>If toxicity resolves to grade 0–1 after dose reduction, return to original dose level after 28 days.<br>If toxicity does not resolve to grade 0-1 despite dose reduction, interrupt study medication for a minimum of 7 days until toxicity has resolved to grade 0–1.<br>After dose interruption, resume study medication reduced by one dose level for 28 days.<br>If skin toxicity is maintained at grade 0–1 at reduced dose, increase study medication to original dose level after 28 days.
	2nd or 3rd occurrence	As for first occurrence, but upon resuming study medication treatment, decrease dosage by one dose level permanently.
	4th occurrence	Study medication should be permanently discontinued on investigator’s discretion and according to patient’s preference.
Grade 3:	1st occurrence	Institute supportive measures and interrupt study medication for a minimum of 7 days and until toxicity has resolved to grade 0–1.<br>When resuming treatment after dose interruption, resume study medication reduced

		by one dose level for 28 days. If skin toxicity is maintained at grade 0–1 at reduced dose, increase study medication to original dose level after 28 days.
	2nd occurrence	As for first occurrence, but upon resuming study medication treatment, decrease dose one dose level permanently.
	3rd occurrence	Study medication should be permanently discontinued on investigator’s discretion and according to patient’s preference.

Prophylactic supportive measures

All subjects are advised to observe the following recommendations (Chu, 2008; Lacouture, 2008) to prevent the development of HFSR.

- Soft shoes or padded insoles to avoid pressure points should be preferably used; constricting footwear should be generally avoided.
- In patients with pre-existing hyperkeratosis a pedicure should be obtained before treatment, and after the pedicure a hydrating cream should be applied immediately to prevent possible exacerbation of the hyperkeratosis.
- Cotton gloves and socks should be worn. Tight fitting clothes should be avoided.
- Skin irritations by extremes of temperatures, especially by hot water (shower, bath etc.), should be avoided.
- Skin moisturizers (lanolin based lotions or a hydrating cream containing urea) should be applied.
- Before going outdoors sunscreen should be applied to exposed areas.
- Activities that place undue stress on the hands and feet such as vigorous exercise should be avoided, especially during the first four weeks of treatment.
- Care should be taken to avoid activities with excessive friction on the skin in the activities of daily life.
- Oral pyridoxine (vitamin B6) has been shown to prevent or delay the onset of HFSR in patients treated with 5-FU, docetaxel, and pegylated liposomal doxorubicin, however results of prospective trials to evaluate the therapeutic value in prevention of HFSR in patients treated with sorafenib are not available.

Therapeutic supportive measures

The following additional supportive measure should be taken after development of HFSR (Chu, 2008; Lacouture, 2008):

- Application of skin moisturizing creams to the affected areas, if not yet applied.
- Thick cotton gloves or socks could be worn at night to prevent further injury and to help maintain moisture.
- Application of urea (20-40%) or salicylic acid (6%) containing cream twice daily may be indicated.
- Cooling of the affected areas may be effective for immediate relief.
- For grade 2 HFSR application of a low potency topical steroid-containing cream to erythematous areas twice daily should be considered (such as 0.05% clobetasol ointment).
- For pain control treatment with topical analgesics such as lidocain 2% or systemic pain medication (such as nonsteroidal anti-inflammatory drug)

9.3 Management of treatment-associated hypertension

Hypertension was frequently observed in trials with sorafenib, usually of low or moderate grade and manageable with standard hypertensive treatments (German SmPC Nexavar®). In this study hypertension will be graded using the NCI CTCAE version 4.0.

The patients will be instructed to perform self-measurements of the blood pressure three times daily during the 8 weeks of the run-in phase with open label sorafenib treatment.

The dose modification schedule for treatment emergent hypertension during treatment with study medication should be followed (see Table 4). Patients' blood pressure (BP) measurements will be monitored and appropriate treatment to effectively control hypertension under study medication is strongly recommended.

Hypertension is defined as blood pressure diastolic ≥ 90 mmHg and systolic ≥ 160 mmHg, or a ≥ 20 mmHg increase in diastolic measurement if the measurement was previously within normal limits.

**Table 4: Management of treatment-emergent hypertension**	
**Grade of event (CTCAE version 4.0)**	**Management/next dose**

Grade 1 CTCAE	Increased frequency of BP monitoring
Grade 2 CTCAE without any other symptoms than elevated BP<br>**and**<br>diastolic BP < 110 mm Hg	Initiation of anti-hypertensive therapy. Study medication should be continued at the same dose level.
Grade 2 CTCAE symptomatic/persistent<br>**or**<br>Grade 3 CTCAE<br>**or**<br>diastolic BP ≥ 110 mm Hg	Treatment with anti-hypertensives. Study medication should be temporarily discontinued until symptoms resolve **and** diastolic BP returns to ≤ 100 mmHg under treatment with anti-hypertensives. Patients requiring a delay of > 28 days should permanently discontinue the study treatment. When restarting study medication the dose has to be reduced by 1 dose level, however the original dose lever may be resumed later if BP monitoring over at least 4 weeks shows BP in the normal range under hypertensive treatment.<br>If diastolic BP not controlled to remain under ≤ 100 mmHg under study medication, the dose has to be reduced by another dose level and the BP must be monitored closely. However, patients requiring a reduction by >2 dose levels should discontinue the study treatment permanently.
Grade 4 CTCAE	Discontinue study medication permanently.

The selection of anti-hypertensive medication used in this setting should be performed at the investigator's discretion, considering possible site-specific treatment guidelines. All medication should be recorded in the CRF.

9.4 Management of treatment-associated diarrhea

Diarrhea is a common side effect of sorafenib but is usually of low-to-moderate grade. With appropriate anti-diarrhoeic treatments, it either resolves while sorafenib is continued, or once sorafenib is discontinued.

Appropriate prophylactic and/or therapeutic drugs that have been reported to be effective in the treatment of sorafenib-associated diarrhea are loperamide (4 mg after first loose stool, 2 mg after every other loose stool with a maximum dose of 12 mg/day) or racecadotril (Tiorfan®) (one 100 mg capsule three times daily).

If necessary, study medication should be temporarily or permanently discontinued on investigator's discretion. In patients requiring a delay of > 28 days the study treatment should be discontinued permanently.

9.5 Management of treatment-associated hematological toxicities

Table 5 lists dose modifications and delays for hematological treatment-associated toxicities.

**Table 5: Hematologic criteria for dose delay and dose modification of sorafenib/placebo**	
**Grade**	**Dose delay/dose modification**
Grade 0-2	No dose delay and no dose modification
Grade 3	No dose interruption.<br>The dose has to be decreased by one dose level. However, if more than two reductions of the original dose level are necessary (such as in case of repeated occurrence) the treatment has to be discontinued permanently.
Grade 4	The study medication must be discontinued until the toxicity resolves to ≤ grade 2. If no recovery occurs within a 28-day interruption, treatment will be discontinued permanently.<br>Resuming treatment after interruption the dose has to be decreased by one dose level. However, if more than two reductions of the original dose level would be necessary (such as in case of repeated occurrence), the treatment has to be discontinued permanently.

9.6 Management of treatment-associated non-hematological toxicities

Table 6 lists dose modifications and delays for non-hematological treatment-associated toxicities others than skin toxicity, hypertension, and diarrhea. Dose delays and dose modification due to nausea and vomiting apply only to patients who did not receive antiemetic premedication.

**Table 6: Non-hematologic criteria for dose delay and dose modification of sorafenib/placebo (except skin toxicity, hypertension and diarrhea)**	
**Grade**	**Dose delay/dose modification**
Grade 0-2	No dose delay and no dose modification
Grade 3 ECG QTc interval prolongation	Permanent discontinuation of study medication
Grade 3 (except ECG QTc interval	The study medication must be discontinued until the toxicity resolves to ≤ grade 2. If no recovery occurs within a 28-day interruption, treatment will be discontinued

prolongation)	permanently.<br>Resuming treatment after interruption the dose has to be decreased by one dose level. However, if more than two reductions of the original dose level would be necessary (such as in case of repeated occurrence) the treatment has to be discontinued permanently.
Grade 4	Permanent discontinuation of study medication

10.0 Concomitant medications

All medication which is considered necessary for the patient's welfare, and which is not expected to interfere with the evaluation of the study drug, may be given at the discretion of the investigator. All concomitant medications (including start/stop dates, dose frequency, route of administration and indication) must be recorded in the patient's source documentation, as well as in the appropriate pages of the CRF.

Chronic treatment with erythropoietin is permitted provided no dose adjustment is undertaken within 2 months prior to the study or during the study.

G-CSF and other hematopoietic growth factors may be used in the management of acute toxicity such as febrile neutropenia when clinically indicated or at the discretion of the investigator; however, they may not be substituted for a required dose reduction. However, prophylactic use of G-CSF, GM-CSF is not permitted.

CYP3A4 inducers (e.g. rifampin, St. John's wort, phenytoin, carbamazepine, phenobarbital, and dexamethasone) may increase metabolism of sorafenib and thus decrease sorafenib concentrations. Since decreased sorafenib concentrations may result in decreased sorafenib efficacy, chronic co-administration of CYP3A4 inducers with sorafenib should be avoided to the extent possible. St. John's wort containing medicinal preparations are generally not permitted.

Patients taking narrow therapeutic index medications should be monitored closely. Treatment with phenytoin, quinidine, carbamazepine, phenobarbital, and cyclosporin is not allowed during study participation.

The following medications **are not allowed** during the study participation, unless the patient is in the follow-up phase and has permanently discontinued any study medication:

- Any investigational medicinal product or experimental therapy

- Any antitumor therapy including chemotherapy, targeted therapy with antiangiogenics agents other than sorafenib, tyrosine kinase inhibitors, or experimental or approved proteins/antibodies, hormonal therapy, immunotherapy
- Radiotherapy or brachytherapy except to eye or bone
- Bone marrow transplant or stem cell rescue
- Hepatic chemoembolization
- Prophylactic use of G-CSF, GM-CSF or erythropoietin. However, chronic treatment with erythropoietin is permitted provided no dose adjustment is undertaken within 2 months prior to the study or during the study
- Any drug that could cause QT-interval prolongation
- Neomycin
- Warfarin, phenprocoumon, heparin, low-molecular heparin or treatment with ASS with a dose > 100 mg daily
- St. John's wort containing medicinal preparations

Sorafenib has the ability to inhibit a variety of liver metabolic enzymes in vitro. The clinical impact of this inhibition in humans taking drugs metabolized by these enzymes is unknown. Therefore, all patients who are taking concomitant medications that are known to be metabolized by the liver should be closely observed for side effects of concomitant medications.

In general, patients should be closely monitored for side effects of all concomitant medications regardless of the path of elimination.

11.0 Study discontinuation

11.1 Premature discontinuation of a single patient from study therapy

A patient has to be discontinued from study treatment if any of the following occurs:

- Tumor progression according to the RECIST criteria 1.1 after eight weeks of open run-in phase or during the run-in phase
- Tumor progression according to the RECIST criteria 1.1 after prior tumor response (CR, PR, SD) unless the patient was randomized to receive placebo during the randomization phase
- Occurrence of any unacceptable toxicity of study treatment according to the schedules for dose modification and dose interruption in section 9.0
- Adverse event(s) whether suspected or not suspected to be related to study treatment that, in the judgment of the investigator, may cause severe or permanent harm to the patient or which rule out continuation of study treatment
- Increase of QTc interval to > 500 ms or of > 60 ms over baseline (screening) value in ECG
- Interruption of study medication ≥ 4 weeks
- Suspected pregnancy or inadequate contraception at any time during study participation
- Subject non-compliance that could place the subject at an unacceptable risk in the investigator’s and/or sponsor’s judgment
- Withdrawal of patient’s consent to study participation
- Concomitant participation in another clinical trial with an investigational medicinal product or experimental therapy
- Lost to follow up
- Death

Subjects have the right to withdraw consent at any time and without giving any reasons without prejudice to his or her future medical care by the investigator or other medical health care personnel at the institution. The investigator will discuss with the subject the most appropriate way to withdraw to ensure the subject’s health. The investigator should encourage

the patient to attend the "end of study treatment visit" and be under medical supervision especially if any toxicities or adverse events have occurred that have not resolved.

11.2 Premature discontinuation of a single trial centre

A trial centre will be discontinued from the study if any of the following occurs:

- Major protocol violations at the trial centre that affect subject's safety or feasibility of the study at the trial centre in the judgment of the sponsor
- Unsatisfactory enrollment with respect to quantity or quality at the trial centre
- Changes at the trial centre (personnel, technical facilities) that prevent to carry out the study in accordance with the study protocol
- Falsification of data or records at the respective trial centre
- Withdrawal of favourable opinion by the respective ethics committee (German Drug Law § 42 a)

11.3 Premature discontinuation of the whole trial

Any new safety findings that could constitute an unacceptable safety risk for the subjects will cause discontinuation of the whole trial.

The Sponsor, the German competent authority and the ethics committee have the right to terminate this clinical study at any time for reasonable medical or administrative reasons. Any possible premature discontinuation of the trial would be documented adequately with reasons being stated, and information would have to be issued according to local requirements (competent authorities, regulatory authorities, and ethics committee).

12.0 Biostatistical analysis

12.1 Overview

This is a prospective, multicenter phase II discontinuation trial, open label in the run-in phase and randomized, double-blind, placebo-controlled in the randomization phase. Patients showing stable disease after 8-week run-in treatment with sorafenib will be randomized to either sorafenib 400 mg bid or placebo 2x2 tablets; all other patients will either continue run-in sorafenib or stop study treatment. All patients will be followed up until disease progression or death. The primary objective will be to compare progression free survival of patients receiving sorafenib versus patients receiving placebo in the randomized subset.

The statistical evaluation will be performed at the CRO ClinAssess GmbH, Leverkusen. Details of the analysis will be described in the statistical analysis plan (SAP) to this study. The SAP will be finalized and signed before database closure.

12.2 Patient populations to be analyzed

All patients who receive at least one dose of study medication (sorafenib as run-in medication, open label sorafenib or randomized to sorafenib or placebo) will be included in the *Full analysis population* being identical to the *Safety population*. This patient set will be used for most secondary efficacy parameters and all safety parameters to be evaluated. Data will be summarized for the following treatment groups: “Open sorafenib continued after run-in”, “No study treatment after run-in”, “Blinded sorafenib after run-in”, “Blinded placebo after run-in”, and “All patients treated with sorafenib”.

The *Randomized population* will include only patients being randomized after the 8-week run-in sorafenib treatment. It is the primary population for the primary efficacy parameter. Data will be summarized for the following treatment groups: “Blinded sorafenib after run-in” and “Blinded placebo after run-in”.

As a sensitivity analysis on the primary efficacy parameter, a per-protocol analysis for the *Per-protocol population* including only eligible patients may be performed, if regarded necessary prior to database closure. Criteria for eligibility (e.g. patients who do not fulfil the selection criteria of the protocol, cause a severe protocol violation, or were not treated long enough to assess cancer status after baseline) will be set in the SAP detailing the statistical evaluation.

To evaluate the secondary efficacy parameter ‘PFS and TTP after unblinding and retreatment with sorafenib’ the *Placebo to sorafenib population* will be defined as subset of all patients of

the Randomized population who are randomized to placebo and retreated with open sorafenib follow-up medication after unblinding.

Appropriate subgroups may be defined in the SAP.

12.3 Statistical methodology

In general, the recorded baseline, efficacy and safety data will be presented using standard descriptive methods. For continuous data, distribution parameters (mean, standard deviation, minimum, median, and maximum) will be computed. For categorical data, frequency counts will be given.

If requested, individual patient data listings will be generated for any study parameter.

With regard to response rates, patients in whom the respective response criteria are not met will be evaluated as non-responders.

Time-to-event data (time to progression or death) will be graphically presented according to Kaplan-Meier. Median time to event as well as estimates for the proportion of patients not having reached the event after appropriate times will be presented. The starting point will be the date of randomization (primary objective) or the first dose of run-in study drug (secondary). Patients not having documented the respective event will be censored with the last date at which it is known that the respective event has not been reached.

The analysis will not be done before 69 events within the *Randomized population* are observed. The two treatment groups will be compared using a one-sided log-rank test with an alpha of 0.1. Kaplan-Meier estimates and survival curves will be presented for each treatment group, as well as the hazard ratio with its confidence interval. Goal of the study is to show higher PFS for patients receiving sorafenib than for patients receiving placebo.

Appropriate 2-sided 80% confidence intervals may be calculated for key data.

Missing data will not be replaced. If necessary, incomplete dates will be imputed appropriately.

To prevent patient's unnecessary exposure to placebo an interim analysis will be performed after about half the patients are evaluable with regard to PFS after randomisation.

A group sequential design according to Pocock (Pocock, 1977) with k=2 steps (one interim analysis) will be applied that does not change the overall level of the one-sided alpha of 0.1.

The only parameter of the interim analysis is PFS after randomisation (primary endpoint).

Step 1 of the interim analysis (obligatory)

Step 1 of the interim analysis will include the randomized patients as follows:

- Already unblinded patients: primary endpoint already reached, these patients will be included into the analysis without censoring, if progression was the reason for unblinding.
- Patients still taking blinded study medication: these patients will not be included.
- Patients treated in the run-in phase: these patients are not evaluable with regard to the primary endpoint, as not randomized yet, thus not included in the interim analysis

Results of the interim analysis based on already regularly unblinded patients (according to protocol) are as follows:

- If the test statistic of the log-rank test exceeds the critical value, superiority of sorafenib is shown. No further patients will be enrolled and treatment allocation will be revealed.
- If the test statistic of the log-rank test does not exceed the critical value, the study will continue.

The applicable critical values result from the Pocock-design (Pocock, 1977).

Explanation: On the cut-off date 31-Jul-2013 19 already unblinded patients have been treated with placebo and 12 with sorafenib during the randomized phase. Under the presumption that patients have been temporally equally randomized 1:1, the conclusion can be drawn from this distribution that PFS in patient allocated to sorafenib is longer than in patients allocated to placebo. Due to the exclusion of not yet unblinded patients from the analysis (by the majority allocated to sorafenib) the log-rank test yields a worse result than it would if all randomized patients would be included into the analysis (as censored ones if applicable). If the long-rank test will have a statistically significant result under these conditions, it would all the more result significant if all randomized patients would have been included into the analysis. Thus it is justified to include only those patients who have been already unblinded until the cut-off date of the interim analysis with the justified risk to underestimate the true p-value.

Step 2 of the interim analysis (optional)

If the necessary statistical significance for termination of the study is not reached by the first step as described above, the interim analysis will be optionally extended to include all randomized patients in a second step.

However, as the further study participation of the still blinded patients must not be jeopardized, allocation to the treatment group and consecutive analysis will be performed by an Independent Data Monitoring Committee (IDMC). It is regarded as sufficient that the IDMC consists of only one external independent statistician who has to deal with the data only this one time. By means of the IDMC the further course of the study will not be jeopardized by the interim analysis. The IDMC will only notify whether superiority of sorafenib has been shown or not (i.e. the study has to be continued).

If superiority of sorafenib can be shown due to the results of the interim analysis, all patients still receiving blinded study medication will be unblinded as soon as possible on a cut-off date. Irrespective of their treatment allocation all patients are allowed to receive further open-label treatment with sorafenib until occurrence of progression. Patients who are receiving open-label sorafenib will receive further treatment with open-label sorafenib until progression. This will also apply to patients in the run-in phase receiving open-label sorafenib who will not be randomized any more. No further patients will be recruited or screened for the study.

If superiority of sorafenib cannot be shown due to the results of the interim analysis, the study will continue as planned until the adapted estimated sample size is reached. The treatment allocation of all patients still receiving blinded study medication is untouched and treatment will continue without any change. In case the study continues the sample size has to be adapted on the basis of the interim analysis according to Hartung (Hartung, 2006).

At the final analysis all parameters of the CRF will be analyzed. All randomized patients will be analyzed with their effective duration of PFS without censoring if progression was the reason for unblinding. Censoring (as date of unblinding) will only be applied, if there was another reason than progression for unblinding.

12.4 Safety evaluation

Safety data will be monitored on an ongoing basis. Reporting of data will occur as described in this protocol.

Safety data to be analyzed include exposure to study drug, type, incidence and severity of adverse events, and laboratory parameters.

The severity of toxicities will be graded according to CTCAE version 4.0 whenever possible.

12.5 Sample size and power considerations

The sample size for this study is determined for the primary efficacy parameter of this study, PFS after date of randomization. Therefore, the number of all patients to be randomized (Randomized population) must be estimated.

It is assumed that the median PFS for patients receiving placebo is 2 months and that improvement by 75% to median PFS of 3.5 months in the sorafenib arm is clinically significant. A total of 69 events are required to detect a difference in median PFS of 2 vs 3.5 months (one-sided, α=0.1, 85% power). Assuming a recruitment period of 36 months and a follow-up for at least 12 months, a total sample size of 2×35=70 patients is required. To account for 10% drop-outs, 2×39=78 patients must be randomized at a minimum.

13.0 Safety data collection, recording and reporting

13.1 Definition adverse event

An adverse event (AE) is (ICH Guideline E6 for Good Clinical Practice [CHMP/CH/135/95], 1.2) “any untoward medical occurrence in a patient or clinical investigation subject administered a pharmaceutical product and which does not necessarily have a causal relationship with this treatment.

An adverse event (AE) can therefore be any unfavorable and unintended sign (including an abnormal laboratory finding), symptom, or disease temporally associated with the use of the medicinal (investigational) product, whether or not related to the medicinal (investigational) product.”

13.2 Definition adverse drug reaction

Adverse drug reactions or adverse reactions (ARs) are all untoward and unintended responses to a medicinal product related to any dose administered.

All adverse events judged by either the reporting investigator or the sponsor as having a reasonable causal relationship to a medicinal product qualify as adverse reactions. The expression reasonable causal relationship means to convey in general that there is evidence or argument to suggest a causal relationship, i.e. a causal relationship between the medicinal product and the adverse event is at least a reasonable possibility, the relationship cannot be ruled out.

A serious AR (SAR) is an AR that meets the definition of serious (provided below).

13.3 Definition serious adverse event

A serious adverse event (SAE) is defined as an adverse event that

- is fatal
- is life threatening (places the subject at immediate risk of death at the time of the event)
- requires in-patient hospitalization or prolongation of existing in-patient's hospitalization
- results in persistent or significant disability/incapacity
- is a congenital anomaly/birth defect

Medical judgment should be exercised in deciding whether an adverse event/reaction is serious in other situations, constituting a significant medical hazard. Important adverse events/reactions that are not immediately life-threatening or do not result in death or hospitalization but may jeopardize the subject or require intervention to prevent one of the other outcomes should also be considered serious.

A hospitalization meeting the regulatory definition for "serious" is any inpatient hospital admission that includes a minimum of an overnight stay in a health care facility. Any adverse event that does not meet one of the definitions of serious (eg. emergency room visit, outpatient surgery, or required urgent investigation) may be considered by the investigator to meet the "other significant medical hazard" criterion for classification as a serious adverse event. Examples include allergic bronchospasm, convulsions, and blood dyscrasias.

Hospitalization for the performing of protocol-required procedures or administration of study treatment is not classified as an SAE, however any AE that occurs during this hospitalization and meets any of the above mentioned criteria of seriousness needs to be reported as SAE.

Further on, AEs occurring during this hospitalization need to be reported according to the reporting procedures for AEs (see section 13.5 and section 13.6).

Progression of the underlying tumor disease (uveal melanoma) and symptoms caused by progression of the underlying tumor disease need not to be reported as SAE in this protocol, unless progression or symptoms of progression are assessed as causally related to any study drug.

13.4 Definition suspected unexpected serious adverse reaction

A suspected unexpected serious adverse reaction (SUSAR) is any SAE assessed as at least possibly related to any study drug either by the investigator or the sponsor and assessed as unexpected or assessed as unexpected with regard to outcome or severity of the event.

13.5 Reporting procedures for all adverse events

The investigator is responsible for ensuring that all adverse events observed by the investigator or reported by subjects are properly captured in the subjects' medical records and the CRF.

Signs, symptoms or medical conditions/diseases that are already present before a subject receives study drug treatment need not to be documented as AEs, but only if they worsen during study duration.

The investigator is responsible for reviewing laboratory test results and determining whether an abnormal value in an individual study subject represents a change from values before the study. In general, abnormal laboratory findings without clinical significance (based on the investigator's judgment) should not be recorded as adverse events; however, laboratory value changes requiring therapy or adjustment in prior therapy are considered adverse events.

For the purpose of AE documentation in the CRF, the National Cancer Institute Common Toxicity Criteria for Adverse Events (NCI-CTCAE), version 4.0 must be used. A copy of the NCI-CTCAE version 4.0 can be downloaded from the following website: http://ctep.cancer.gov/protocolDevelopment/electronic_applications/ctc.htm.

All trial site personnel involved in the study must have access to a copy of the CTCAE version 4.0.

The following adverse event attributes have to be documented:

- Adverse event diagnosis or syndrome(s), preferably as term, if known, otherwise, if not known signs or symptoms
- Date of onset of AE
- Date of resolution of AE
- Severity (CTCAE Grade) or if not specified in CTCAE as follows:
  - Grade I: mild (awareness of sign, symptom, or event, usually transient, requiring no special treatment and generally not interfering with usual daily activities)
  - Grade II: moderate (discomfort that causes interference with usual activities; usually ameliorated by basic therapeutic maneuvers)

  - Grade III: severe (incapacitating with inability to do usual activities or significantly affects clinical status and warrants intervention. Hospitalization may or may not be required)
  - Grade IV: life-threatening (immediate risk of death; requires hospitalization and clinical intervention)
  - Grad V: death
- Seriousness (In case an AE fulfils criteria of SAE, SAE reporting is required)
- Assessment of relatedness to study treatment
- Measures taken with regard to study medication

Medically significant adverse events considered possibly related to the investigational product by the investigator or the sponsor will be followed until resolved or considered as resolved with sequelae.

It will be left to the investigator's clinical judgment to determine whether an AE is related and of sufficient severity to require the subject's removal from treatment. A subject may also voluntarily withdraw from treatment due to what he or she perceives as an intolerable AE. If either of these situations arises, the subject should be strongly encouraged to undergo an end-of-study treatment assessment and be under medical supervision until symptoms cease or the condition becomes stable.

13.6 Reporting procedures serious adverse events

SAEs of individual patients will be collected and recorded from the first day of application of sorafenib until 30 days after the last application of sorafenib or placebo.

At least the following serious adverse event attributes should be reported:

- Serious adverse event diagnosis or syndrome(s), preferably as CTCAE term, if known, otherwise, if not known signs or symptoms
- Description of serious adverse event
- Date of onset of SAE
- Outcome of SAE
- Date of resolution of SAE/ ongoing
- Severity (CTCAE grade) of SAE
- Criterion/Criteria of seriousness
- Dose, duration and last application of study drug before onset of event

- Assessment of causal relationship to study treatment
- Information about medically important concomitant medication or concurrent illnesses
- Measures taken with regard to study drug

SAEs have to be reported by the investigator of the respective trial site within 24 hours after recognition or receiving knowledge of the SAE by fax to ClinAssess (see contact information below) being contracted with this task by the sponsor to the following address. If missing information about a SAE cannot be collected within 24 hours, the SAE should be reported within 24 hours and the missing information sent later as a follow-up report as soon as possible.

ClinAssess GmbH
Birkenbergstr. 82
51379 Leverkusen
Germany
Tel.: +49 (0) 2171 36 336 -0
Fax: +49 (0) 2171 36 336 -55

ClinAssess will immediately convey this information to the coordinating investigator as the sponsor's (Universitätsklinikum Essen, Hufelandstr. 55, 45122 Essen) authorized representative, who will medically review all SAEs.

All serious adverse events will be forwarded to the Bayer Vital GmbH local safety officer by the CRO as well:

Bayer Vital GmbH
Bayer Schering Pharma Medizin
Arzneimittelsicherheit
Gebäude K 56
51368 Leverkusen
Tel.: +49 (0) 214 3051 340
Fax: +49 (0) 214 3051 341

It is possible that Bayer HealthCare may request follow-up information from the sponsor:

If an ongoing SAE has resolved or changed in any condition or if there is new information about the event, a follow-up SAE form has to be sent by the investigator and will be distributed as described above.

13.7 Sponsor's reporting responsibilities

Every SAE will be assessed by the sponsor's authorized representative with regard to

- Relationship to study treatment (sorafenib or placebo)
- Expectedness
- Changes of the benefit-risk relation of the study

Expectedness of any SAR will be assessed by means of the latest German Summary of Product Characteristics for Nexavar® as source data.

Further on, the sponsor's authorized representative will decide, whether unblinding of the respective patient's treatment allocation (either sorafenib or placebo) is necessary, when the subject received blinded study medication during the randomization phase.

Every SAE, being assessed by either the investigator or the sponsor as at least possibly related to study drug und assessed as being either unexpected or unexpected with regard to outcome or severity of the event will be reported as SUSAR to the competent authorities, responsible ethics committee and investigators of the trial in line with the national regulations in effect (German drug law (AMG) and GCP-V).

Fatal or life-threatening SUSARs must be reported within 7 days, all others need to be reported within 15 days. Also all events which can change the benefit-risk ratio of the study drug have to be reported within 15 days in the same way as SUSARs.

All SUSARs related or possibly related to sorafenib and their follow-up reports will be reported to Bayer HealthCare at the same time as submission to the competent authority. A copy of the respective SUSAR report will be faxed to Bayer HealthCare at the same time of such submission.

Once a year throughout the clinical trial or on demand, the sponsor will provide the competent authority and the responsible ethics committee with the annual safety report in accordance with national regulations (GCP-V).

13.8 Pregnancies

A female patient must be instructed to immediately inform the investigator if she becomes pregnant during the study. The study treatment must be stopped immediately and the patient must be withdrawn from the study. Pregnancies after the completion of the last treatment cycle must also be reported to the investigator. The investigator should counsel the patient,

discuss the risks of continuing the pregnancy, and possible effects on the fetus. Monitoring of the patient should continue until conclusion of the pregnancy.

14.0 Data handling and quality control

14.1 Data recording and reporting

Data will be recorded and reported until the last subject will have completed the trial.

14.2 Data collection

Data will be entered in a central database by assigning a unique subject number. No identification of subjects will be possible.

For quality management, data will be verified by double entry into the central database. Data corrections will be handled similar to new data entry. The audit trail of database ensures that changes in data can be followed by time point and identification of data manager.

14.3 Study monitoring

Periodic monitoring of the trial will be performed on-site in the trial centres, i.e. in terms of visits by Clinical research associates (CRAs) to ensure the safety of the trial participants, the trial itself as well as the correctness of the collected data. Preferentially the CRA will check:

- Adherence to eligibility criteria
- Availability of the subject's informed consent
- Adherence to treatment according to the trial protocol
- Completeness of the trial documents in the trial centre
- Safety parameters

After being monitored the collected data will be transferred into the central database.

15.0 Regulatory considerations

15.1 Subject insurance

According to § 40 para. 1 No. 8 and para. 3 AMG the sponsor of the study will obtain insurance coverage for eventually occurring damage caused by the treatment or any actions taken according to the treatment plan.

15.2 Approval by the competent authorities and ethics committee

The protocol for this study has been designed in accordance with the general ethical principles outlined in the Declaration of Helsinki and the ICH-GCP guidelines.

The Sponsor will be responsible to obtain authorization for the clinical trial by the competent authority and a favourable opinion by the relevant ethics committees prior to the initiation of the study in accordance with the German Drug Law, GCP-V and other national and European regulations.

Any amendments to the protocol after receipt of authorization/favourable opinion for the clinical trial by the competent authorities and the ethics committee must get approval by the competent authorities and the relevant ethics committee as well in accordance with the German Drug Law, GCP-V and other national and European regulations.

Any advertisements used to recruit subjects for the study must be reviewed and approved by the ethics committee prior to use.

15.3 Informed consent

The investigator must obtain written informed consent of a subject prior to any study related procedures including the documentation of results of clinical routine procedures for study purposes as set forth in the GCP-ICH guidelines and German Drug Law (AMG). Documentation that informed consent occurred prior to the subject's entry into the study and the informed consent process should be recorded in the subject's source documents. The original consent form, signed and dated by the subject and by the person consenting the subject prior to the subject's entry into the study, must be maintained in the investigator's study files.

16.0 Subject confidentiality and data protection

The Sponsor affirms the subject's right to protection against invasion of privacy.

All records identifying the patients will be kept confidential and, to the extent permitted by the applicable laws and/or regulations, will not be made publicly available.

The investigator must assure that the patient's anonymity will be maintained and that the identities are protected from unauthorized parties. The investigator should maintain documents not for submission to the sponsor e.g. subjects written consent forms, in strict confidence. On CRF's and other documents patients should not be identified by their names or birth dates. All clinical and scientific data are collected under a patient-identification code.

All data transfer with the study centres will be made without any exception via the patient-code. All participating study centres are obliged to keep a strictly confidential patient identification list at a safe locked place.

Persons, who are authorized by the sponsor or regulatory authorities (e.g. CRA's, auditors or representatives of regulatory authorities) may be permitted to patient-related data medical records relevant to the study for review or inspections respectively in accordance with local laws and the subject's statement in the informed consent.

All study related documents have to be kept in a safe, locked place for a minimum of 10 years, patient identification lists for a minimum of 15 years in accordance with international and national regulations.

17.0 References

Adam R, Chiche L, Aloia T, Elias D, Salmon R, Rivoire M, et al. Hepatic resection for noncolorectal nonendocrine liver metastases: analysis of 1,452 patients and development of a prognostic model. Ann Surg 2006; 244:524-35

Amery W, Dony J: A clinical trial design avoiding undue placebo treatment. J Clin Pharmacol 1975; 15 (10):674-9

Bayer Schering Pharma AG. Nexavar® Summary of product characteristics, 2009

Baba HA; Wohlschlaeger J, Hilgard P, Sotiropoulos GC, Takeda A, Beckebaum S, Schmitz KJ: Phosphorylation of p70S6 kinase predicts overall survival in patients with clear margin-resected hepatocellular carcinoma. Liver Int. 2009 Mar; 29 (3):399-405

Barak V, Frenkel S, Kalickman I, Maniotis AJ, Folberg R, Pe'er J. Serum markers to detect metastatic uveal melanoma. Anticancer Res. 2007; 27:1897-1900

Becker JC, Terheyden P, Kämpgen E, Wagner S, Neumann C, Schadendorf D: Treatment of disseminated ocular melanoma with sequential fotemustine, interferon alpha, and interleukin 2. Br J Cancer 2002, 87:840-5

Calipel A, Mouriaux F, Glotin AL, Malecaze F, Faussat AM, Mascarelli F. Extracellular signal-regulated kinase-dependent proliferation is mediated through the protein kinase A/B-Raf pathway in human uveal melanoma cells. J Biol Chem 2006; 281:9238-50

Chu D, Lacouture ME, Fillos T, Wu S. Risk of hand-foot skin reaction with sorafenib. A systematic review and meta-analysis. Acta Oncologica 2008; 47:176-86

Eisen T, Ahmad T, Flaherty JT, Gore M, Kaye S, MaraisR, Gibbens I, Hackett S et al: Sorafenib in advanced melanoma: a Phase II randomised discontinuation trial analysis. Br J Cancer 2006; 95(5):581-6

Eisenhauer EA, Therasse P, Bogaerts J, Schwartz LH, Sargent D, Ford R et al. New response criteria in solid tumours: Revised RECIST guideline (version 1.1). Eur J Cancer. 2009 Jan;45(2):228-47

Escudier B, Eisen T, Stadler WM, Szczylik C, Oudard S, Siebels M et al for the TARGET Study Group. Sorafenib in advanced clear-cell renal-cell carcinoma. New Engl J Med 2006; 356**:**125-34

Fecher, L.A., Amaravadi, R. and Schuchter, L.M. Effectively targeting BRAF in melanoma: a formidable challenge. Pigment Cell Melanoma Res 2008; 21**:** 410-411

Flaherty LE, Unger JM, Liu PY, Mertens WC, Sondak VK. Metastatic melanoma from intraocular primary tumors: the Southwest Oncology Group experience in phase II advanced melanoma clinical trials. Am J Clin Oncol 1998; 21(6):568-72

Foss AJ, Guille MJ, Occleston NL, et al. The detection of melanoma cells in peripheral blood by reverse transcription-polymerase chain reaction. Br J Cancer , 1995; 72:155-9

Gragoudas ES, Egan KM, Seddon JM, Glynn RJ, Walsh SM, Finn SM, Munzenrider JE, Spar MD. Survival of patients with metastases from uveal melanoma. Ophthalmology 1991;98(3):383-9

Harbour JW, Onken MD, Roberson EDO, Duan S, Cao L, Worley LA, Council ML, Matatall KA, Helms C, Bowcock AM. Frequent mutation of *BAP1* in metastasizing uveal melanomas. Science 2010; 330:1410-3

Haritoglou I, Wolf A, Maier T, Haritoglou C, Hein R, Schaller UC. Osteopontin and 'melanoma inhibitory activity': comparison of two serological tumor markers in metastatic uveal melanoma patients. Ophthalmologica. 2009; 223(4):239-43

Hartung J: Flexible designs by adaptive plans of generalized Pocock- and O'Brien-Fleming-type and by self-designing clinical trials. Biom J. 2006; 48:521-36

Ijland SA, Jager MJ, Heijdra BM, Westphal JR, Peek R. Expression of angiogenic and immunosuppressive factors by uveal melanoma cell lines. Melanoma Res 1999;9:445–50

Jain L, Venitz J, Figg W: Randomized discontinuation trial of sorafenib (BAY 43-9006). Cancer Biol Ther 2006;5(10):1270-72

Kalkmann J, Richly H, Scheulen ME, Forsting M, Stattaus J. Diffusion-weighted imaging for liver lesion detection in patients with metastatic uveal melanoma – how to detect pseudoprogression under antiangiogenetic therapy? Oral presentation at ECR 2010;Eur Radiol 20:nn

Kath R, Hayungs J, Bornfeld N, Sauerwein W, Höffken K, Seeber S: Prognosis and treatment of disseminated uveal melanoma. Cancer 1993;72(7):2219-23

Kim S, Takahashi H, Lin WW, Descargues P, Grivennikov S, Kim Y, Luo JL, Karin M: Carcinoma produced factors activate myeloid cells via TLR2 to stimulate metastasis. Nature 2009; 457 (7225)

Keilholz U, Goldin-Lang P, Bechrakis NE, Max N, Letsch A, Schmittel A, Scheibenbogen C, Heufelder A, Eggermont A, Thiel E. Quantitative detection of circulating tumor cells in cutaneous and ocular melanoma and quality assessment by real-time reverse transcriptase-polymerase chain reaction. Clin Cancer Res 2004; 10:1605-12

Klein CA: Cancer. The metastasis cascade. Science 2008; 321:1785-7

Klingenstein A, Haug AR, Nentwich MM, Messmer EM, Schaller UC. Hepatic metastases in CUP (cancer of unknown primary) and painful amaurosis. Ophthalmologe. 2010 Oct;107(10):947-50.

Kopec J, Abrahamowicz M, Esdaile JM. Randomized discontinuation trials: utility and efficiency. 1993; 46(9):959-71

Lacouture ME, Wu S, Robert C, Atkins MB, Kong HH, Guitart J, et al. Evolving strategies for the management of hand-foot skin reaction associated with the multitargeted kinase inhibitors sorafenib and sunitinib. The Oncologist 2008; 13: 1001-1011

Llovet JM, Ricci S, Mazzaferro V, Hilgard P, Gane E, Blanc JF et al for the SHARP Investigators Study Group. Sorafenib in advanced hepatocellular carcinoma. New Engl J Med 2008; 359**:**378-90

Mariani P, Piperno-Neumann S, Servois V, Berry MG, Dorval T, Plancher C, et al. Surgical management of liver metastases from uveal melanoma: 16 years' experience at the Institut Curie. Eur J Surg Oncol 2009; 35:1192-7

Odashiro DN, Odashiro AN, Pereira PR, Godeiro K, Antecka E, Di Cesare S, Burnier Jr MN: Expression of EpCAM in uveal melanoma. Cancer Cell Int 2006; 6:26

Oken MM, Creech RH, Tormey DC, Horton J, Davis TE, McFadden ET et al. Toxicity and response criteria of the Eastern Cooperative Oncology Group. Am J Clin Oncol 1982;5:649-55

Pacey S, Ratain MJ, Flaherty KT, Kaye SB, Cupit L, Rowinsky EK, Xia C, O'Dwyer PJ, Judson IR. Efficacy and safety of sorafenib in a subset of patients with advanced soft tissue sarcoma from a Phase II randomized discontinuation trial. Invest New Drugs. 2009 Dec 18. [Epub ahead of print]

Patterson DM, Padhani AR, Collins DJ. Technology Insight: water diffusion MRI – a potential new biomarker of response to cancer therapy. Nat Clin Pract Oncol 2008; 5:220-33

Peters S, Voelter V, Zografos L, Pampallona S, Popescu R, Gillet M, et al. (2006) Intra-arterial hepatic fotemustine for the treatment of liver metastases from uveal melanoma: experience in 101 patients. Ann Oncol 2006; 17:578-83

Pocock SJ: Group sequential methods in the design and analysis of clinical trials, Biometrika 1977; 64:191-9

Podsypanina K, Du YC, Jechlinger M, et al: Seeding and propagation of untransformed mouse mammary cells in the lung. Science , 2008; 321:1841-4

Prescher G, Bornfeld N, Hirche H, Horsthemke B, Jöckel KH, Becher R. Prognostic implications of monosomy 3 in uveal melanoma. Lancet 1996; 347:1222-5

Ratain MJ, Eisen T, Stadler WM, Flaherty KT, Kaye SB, Rosner GL, Gore M, Desai AA et al. Phase II placebo-controlled randomized discontinuation trial of sorafenib in patients with metastatic renal cell carcinoma. J Clin Oncol. 2006; 24(16):2505-12. Epub 2006 Apr 24.

Reiniger IW, Schaller UC, Haritoglou C, Hein R, Bosserhoff AK, Kampik A, Mueller AJ. Melanoma inhibitory activity" (MIA): a promising serological tumour marker in metastatic uveal melanoma. Graefes Arch Clin Exp Ophthalmol. 2005 Nov;243(11):1161-6

Rosner GL, Stadler W, Ratain MJ: Randomized discontinuation design: Application to cytostatic antineoplastic agents. J Clin Oncol 2002; 20(22):4478-84

Saraiva VS, Caissie AL, Segal L, Edelstein C, Burnier MN. Immunohistochemical expression of phospho-Akt in uveal melanoma. Melanoma Res 2005; 15:245–50

Scheulen ME, Nokay B, Richtly H, Hoffmann AC, Kalkmann J, Stattaus J, Bornfeld N, Schuler MH, Hense J: Register trial of sorafenib for patients with metastatic uveal melanoma. Admitted as abstract #83211 for ASCO 2011

Schmidt J, Bosserhoff AK. Processing of MIA protein during melanoma cell migration. Int J Cancer 2009 Oct 1;125(7):1587-94.

Schmittel A, Scheulen ME, Bechrakis NE, Strumberg D, Baumgart J, Bornfeld N, Foerster MH, Thiel E, Keilholz U. Phase II trial of cisplatin, gemcitabine and treosulfan in patients with metastatic uveal melanoma. Melanoma Res 2005; 15(3):205-207

Schmittel A, Schuster R, Bechrakis NE, Siehl JM, Foerster MH, Thiel E, Keilholz U. A two-cohort phase II clinical trial of gemcitabine plus treosulfan in patients with metastatic uveal melanoma. Melanoma Res 2005; 15(5):447-51

Schmittel A, Schmidt-Hieber M, Martus P, Bechrakis NE, Schuster R, Siehl JMet al: A randomized phase II trial of gemcitabine plus treosulfan versus treosulfan alone in patients with metastatic uveal melanoma. Ann Oncol 2006; 17:1826-9

Schmitz KJ, Wohlschlaeger J, Lang H, Sotiropoulos GC, Malago M, et al. Activation of ERK and AKT signalling pathway predicts poor prognosis in hepatocellular carcinoma and ERK activation in cancer tissue is associated with hepatitis C virus infection. J Hepatol. 2008 Jan; 48(1):83-90

Schubert W., Bonnekoh B., Pommer A. J. et al. Analyzing proteome topology and function by automated multidimensional fluorescence microscopy. Nature Biotechnology 2006; 24:1270-8. Accompanied by News and Views.

Schuster R, Bechrakis NE, Stroux A, Busse A, Schmittel A, Scheibenbogen C, Thiel E, Foerster MH, Keilholz U. Circulating tumor cells as prognostic factor for distant metastases and survival in patients with primary uveal melanoma. Clin Cancer Res 2007; 13:1171-8

Triozzi PL, Eng C, Singh AD. Targeted therapy for uveal melanoma. Cancer Treat Rev 2008, 34**:**247-58

Vahrmeijer AL, van de Velde CJH, Hartgrink HH, Töllenaar RAEM. Treatment of melanoma metastases confined to the liver and future perspectives. Dig Surg 2008; 25:467-72

Wilhelm S, Chien DS. BAY 43-9006: preclinical data. Cur Pharmaceut Design 2002; **8:**2255-7

Van Raamsdonk CD, Bezrookove V, Green G, Bauer J, Gaugler L, O'Brien JM et al. Frequent somatic mutations of GNAQ in uveal melanoma and blue naevi. Nature 2009, 457: 599-602

Weber A, Hengge UR, Urbanik D, et al. Absence of mutations of the BRAF gene and constitutive activation of extracellularregulated kinase in malignant melanomas of the uvea. Lab Invest 2003; 83:1771–6

Wilhelm SM, Carter C, Tang L, Wilkie D, McNabola A, Rong H et al. BAY 43-9006 exhibits broad spectrum oral anti-tumor activity and targets the Raf/MEK/ERK pathway and receptor tyrosine kinases involved in tumor progression and angiogenesis. Cancer Res 2004; 64**:**7099-109

18.0 Appendices

Appendix I: ECOG Performance Status

ECOG Performance Status (Oken, 1982)

SCORE	DESCRIPTION
**0**	Fully active, able to carry on all pre-disease performance without restriction.
**1**	Restricted in physically strenuous activity but ambulatory and able to carry out work of a light or sedentary nature, e.g., light house work, office work.
**2**	Ambulatory and capable of all self-care but unable to carry out any work activities. Up and about more than 50% of waking hours.
**3**	Capable of only limited self-care, confined to bed or chair more than 50% of waking hours.
**4**	Completely disabled. Cannot carry on any self-care. Totally confined to bed or chair.
**5**	Dead.

Appendix II: New York Heart Association Functional Classification

Class	Description
I	Patients with cardiac disease but without resulting limitation of physical activity. Ordinary physical activity does not cause undue fatigue, palpitation, dyspnea, or anginal pain.
II	Patients with cardiac disease resulting in slight limitation of physical activity. They are comfortable at rest. Ordinary physical activity results in fatigue, palpitations, dyspnea, or anginal pain.
III	Patients with cardiac disease resulting in marked limitation of physical activity. They are comfortable at rest. Less than ordinary activity causes fatigue, palpitations, dyspnea, or anginal pain.
IV	Patients with cardiac disease resulting in inability to carry on any physical activity without discomfort. Symptoms of heart failure or the anginal syndrome may be present even at rest. If any physical activity is undertaken, discomfort is increased.

Appendix III: Pharmacokinetic and pharmacodynamic sample collection and handling

Materials and Labeling

Blood must be collected in specific blood collection tubes: Use two 2.7 ml EDTA S-Monovettes (Sarstedt AG & Co., Nümbrecht, Germany 2.7 ml K3E, No./REF 05.1167). Resulting plasma samples must be stored in polypropylene storage tubes. No tubes with separation gel should be used.

Use of alternative materials will not result in a protocol amendment if pre-approved by the Bioanalysis Scientist.

Labels should be applied to the sample tubes as follows:

- Apply labels to the sample tube so that they do not overlap and obscure any information. If possible expose an area between 2 ends of the label to allow viewing of the contents of the tube.
- Do not alter the orientation of the label on the sample tube.
- Apply labels to all tubes in the same manner.

Preparation of PK/PD Samples

- Collect two tubes of 2.7 ml blood using the EDTA S-Monovettes.
- Record the exact date and time of sampling in the CRF.
- Gently invert the tubes 8 to 10 times to afford mixing, before processing.
- Samples must be processed within 45 minutes. Therefore, put the samples on ice.
- Centrifuge one sample immediately (2500 x g, 4°C, 15 min) to yield at least 1.0 ml of plasma.
- Transfer the generated EDTA-plasma into cryo-tubes labeled with study subject, and time point of blood drawing. Transfer all separated plasma immediately with a clean, disposable polyethylene pipette (use 1 new pipette per sample) into two pre-labeled storage tubes (1.8 ml cryo.s tubes, greiner bio-one). One tube should contain at least 500 µl of plasma.
- The second tube has to be assigned to the “FACS”-unit.

- Store plasma samples and the processed EDTA S-Monovettes in an upright position in a freezer, at approximately -78°C or lower.
- The time between blood collection and freezing the plasma will not exceed 45 min.
- Questions regarding handling the pharmacokinetic/pharmacodynamic specimens should be addressed to Dr. Ralf Hilger.
- Alternative procedures will not result in a protocol amendment if approved by the Bioanalytical Scientist.

Appendix IV: Identification of biomarkers in circulating tumor cells

Background: As recently discussed hematogenous metastasis may be an early event in tumor evolution of solid malignancies[1,2]. As for uveal melanoma the development of liver metastasis is a frequent event, hematogenous metastasis seems to be the primary way of tumor spread[3-5]. Up to date studies used tyrosinase and MelanA/MART1 mRNA Expression Analysis to quantify tumor load in peripheral blood and indirectly quantify the increase or decrease of circulating tumor cells to estimate the individual risk for development of metastasis and to predict therapeutic response to locoregional or systemic therapy. Non-specific expression analysis from total blood RNA and the potentially small range of identifiable prognostic and predictive markers obtained, seem to be pitfalls of this approach. We developed a technique to specifically enrich circulating tumor cells and to confirm their existence by immunohistochemistry. After confirmation of tumor cells we use Expression Analysis from highly specific cell populations from circulating tumor cells to then identify and validate new biomarkers for the assessment of aggressiveness of the tumor and for predicting the response and survival rate of each individual based on the genetic profile.

Method: To this aim mononuclear cells and circulating tumor cells will be enriched from peripheral venous blood using a specifically designed buoyant density gradient centrifugation. Then immuno-magnetic beads with anti-CD15, anti-CD19 and anti-CD45 monoclonal antibodies will be used to negatively select hematopoietic cells[6]. The remaining cell suspension will then be immunohistochemically stained by a trained pathologist after fixation on a glass slide. In this way circulating tumor cells can be identified, microdissected and RNA respectively DNA can be isolated. Linear Amplification techniques will be used to yield higher amount of DNA and RNA for subsequent Analysis. Gene/SNP-Array and quantitative real-time reverse transcriptase-polymerase chain reaction (RT-PCR) will be used to examine polymorphisms and gene expression associated to progression-free and overall survival of patients with metastasized uveal melanoma. The patient group treated with sorafenib will be used to identify genes that are correlated with response to this therapy.

20 ml of whole blood will be drawn from each patient on day 1 and 14 of the 8 week run-in Phase. After 8 weeks of therapy another 20 ml will be drawn. These 3 time-points will then be used to identify markers that are of predictive value for therapeutic response.

1. Klein CA: Cancer. The metastasis cascade. Science 321:1785-7, 2008

2. Podsypanina K, Du YC, Jechlinger M, et al: Seeding and propagation of untransformed mouse mammary cells in the lung. Science 321:1841-4, 2008
3. Foss AJ, Guille MJ, Occleston NL, et al: The detection of melanoma cells in peripheral blood by reverse transcription-polymerase chain reaction. Br J Cancer 72:155-9, 1995
4. Schuster R, Bechrakis NE, Stroux A, et al: Circulating tumor cells as prognostic factor for distant metastases and survival in patients with primary uveal melanoma. Clin Cancer Res 13:1171-8, 2007
5. Keilholz U, Goldin-Lang P, Bechrakis NE, et al: Quantitative detection of circulating tumor cells in cutaneous and ocular melanoma and quality assessment by real-time reverse transcriptase-polymerase chain reaction. Clin Cancer Res 10:1605-12, 2004
6. Odashiro DN, Odashiro AN, Pereira PR, et al: Expression of EpCAM in uveal melanoma. Cancer Cell Int. 2006 Nov 24;6:26.

Appendix V: Specifications for whole body MRI in combination with whole body DWI

Examination: whole body MRI in combination with whole body DWI

Device: 1.5 T Siemens Avanto

Preparation: indwelling venous catheter

Contrast medium: Gadovist 0.1mmol/kg as single dose => e.g. 7ml in a person with 70 kg body weight

Sequences:

Whole body:

1. Neck to groin: T2 Trufi coronal (7 mm, 20% Gap)
2. Neck to groin: EP-DWI axial (4 mm, 3 b-values: 50, 500, 1000 mm/s2)
3. Neck to groin: T1 fl2 in/opp phase axial (7 mm, 20% Gap)
4. Neck to groin: T2 HASTE axial (7 mm)
5. Liver: dynamic vibe axial (3,5 mm):
6. Neck to groin: T1 fl 2d with fat saturation axial (7 mm, 20% Gap)

Technical parameters:

Sequence	Fatsuppr.	TR	TE	ST	Gap	Base Res	Averages	B value (s/mm^2)	PAT
T2 HASTE ax.	Fat sat.	1000	123	7	20%	256	0		
EP-DWI ax.	SPAIR	11400	76	4	0%	192	4	50, 500, 1000	2

Patients who exclusively suffer from liver metastases during the staging evaluation (screening) and are not suspected to suffer progression due to incidence of extrahepatic metastases during any time of the study course will receive MRI of the liver including DWI of the liver in the identical sequences during the following MRI examinations, patients with evidence of extrahepatic metastasis at staging (screening) or suspicion of extrahepatic metastasis during the study will undergo further whole body MRI including whole body DWI in the identical sequences. Scans have to include evaluations of all suspected sites of the disease and must be obtained with identical modality and technique to those obtained at baseline.

Appendix VI: Tumor fingerprinting by MELC

Background: The differential diagnosis of pigmented lesions is still very challenging due to the lack of reliable biomarkers. Recent insights into the enormous diversity of cancer on the genomic level and astonishing potential of cancer cells to change their genomic makeup and escape have greatly dampened early expectations for gene markers. As a consequence, researchers are turning back to proteins in tissue and blood of cancer patients in search for new biomarkers. So far the simultaneous staining of more than three antigens in a tissue sample was difficult due to technical reasons. The limited number of distinguishable colour signals, different intensities of individual signals and the resulting false-positive co localization effects prohibited a combinatorial analysis of multiple antigens in a given cell or tissue sample. The development of the MELC technique closed this technical gap and allows the simultaneous staining of one sample with numerous antibodies (1). Using established sets of antibodies in a combinatorial manner, the MELC-procedure provides a highly specific antigen fingerprint for each tumour tissue sample, which may serve as a predictor for the treatment response in “targeted therapy” using novel cancer drugs like Sorafenib (Nexavar®) alone or in combination with other drugs.

Method:

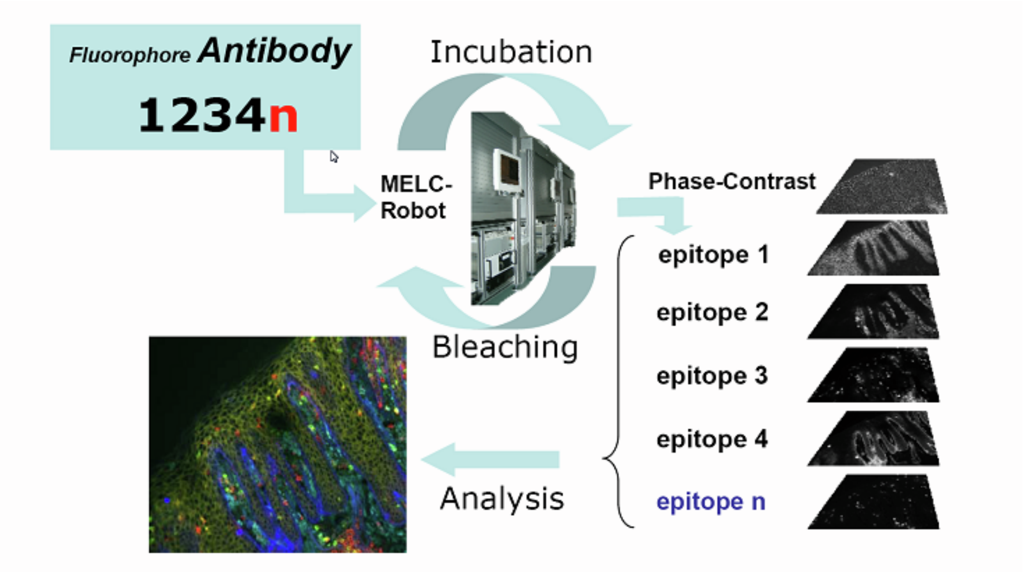

The MELC technology starts with a fixed and mounted tissue or cell sample and subjects it to fully automated cycles of fluorescent staining, imaging and photobleaching. Each cycle can apply an antibody specific for a different protein so that the result is a set of images of the

distributions of many different proteins for the same field. The digitalisation of the obtained data/images allows a proper spatial and topographical localization of each antigen as well as semi-quantification of positive signals. Furthermore, the MELC technique is automated and standardized and hence suitable to process a large amount of samples in a rather short time period.

Goal:

Using sets of antibodies that recognize primary and metastatic uveal melanoma the Toponome Research facility will characterize tumor tissue from patients successfully/non-successfully treated with Sorafenib to establish fingerprints predicting treatment response.

1 Schubert W., Bonnekoh B., Pommer A. J. et al., (2006). Analyzing proteome topology and function by automated multidimensional fluorescence microscopy. Nature Biotechnology. 24, 1270-8. Accompanied by News and Views.
